# Progressive structural and functional change in horses: a conceptual framework for systemic equine (patho-)physiology

**DOI:** 10.3389/fvets.2026.1767386

**Published:** 2026-04-07

**Authors:** Maren Diehl, Katharina Bader

**Affiliations:** 1 Independent Researcher, Erfweiler, Germany; 2 Independent Researcher, Kernen im Remstal, Germany

**Keywords:** equine biomechanics, functional anatomy, hoof morphology, locomotion, lumbosacral joint, musculoskeletal disorders, rehabilitation, systemic pathology

## Abstract

A wide range of locomotor, postural, and behavioral pathophysiology in horses—often grouped under terms such as Topline Syndrome, Myofascial Dysfunction, or Poor Posture Syndrome—lack a coherent systemic explanation. These presentations share reduced performance, stability, and resilience, yet are commonly managed through isolated symptom-focused interventions. To address this gap, we propose progressive structural and functional change (PSF) as a systemic framework describing progressive reorganization processes within the equine body that link fragmented pathological domains into a coherent pattern of structural and functional (dys-)regulation. PSF comprises two trajectories: progressive structural and functional loss (PSF^−^), driven by a maladaptive self-amplifying reorganization of motion patterns, and progressive structural and functional gain (PSF^+^), a self-stabilizing pattern of physiological recovery and improving functional organization. We identify functional inversion as central mechanism driving PSF^−^. It denotes a systemic reversal of physiological force directions and load-transfer roles, characterized by a persistently open lumbosacral joint, a shift in stance-phase timing, and reciprocal remodeling of fore- and hind hoof morphology. These interdependent changes are interpreted as creating conditions consistent with a broad spectrum of secondary symptoms and pathologies. This Hypothesis and Theory article presents a concept-driven framework derived from applied field observations in horse training and rehabilitation, rather than from institutionally based academic research in equine science. It is based on long-term exploratory field observations and includes ten retrospectively documented case studies of horses followed over several years. The heterogeneous cases (varying breed, age, training background) serve as illustrative examples of recurring functional patterns rather than as controlled sample. Our observations suggest that restoring physiological lumbosacral function, facilitating horse-initiated horizontal tension toward the bit, and managing hoof morphology can support the system shift toward PSF^+^, while unresolved local pathologies limit systemic reorganization. The PSF framework integrates unconnected clinical findings into a conceptually defined systemic process, identifies measurable screening targets, and may support practitioners in prioritizing rehabilitation strategies. It generates falsifiable predictions that can be examined at two levels—through low-threshold, practice-based observational criteria and through advanced biomechanical measurement approaches—thereby enabling both practice-based application by horse owners and scientific validation. The framework aims to complement welfare-oriented veterinary prevention, diagnostics, and rehabilitation.

## 1 Introduction

Across languages and disciplines, equine performance decline, reduced stability, and premature tissue wear are described by trainers, therapists, and horse owners using imprecise or inconsistent terminology. Although these systemic conditions seem to be widespread, their suspected link to altered structural organization and systemic coordination remains insufficiently defined according to our findings (bibliographic search strategy see Supplementary material). As a result, diagnosis and treatment frequently target isolated symptoms within fragmented pathological domains rather than investigating their underlying causes.

Epidemiological data underline the clinical relevance of musculoskeletal dysfunction in horses. Tendon and ligament injuries account for approximately 43–54% of all musculoskeletal injuries in equine athletes, as reviewed by Ehrle et al. (1). Clinical investigations further demonstrate a strong association between lameness and back problems (2), and a high prevalence of back pain in sport horses (3). In addition, recent survey-based research reports a high prevalence of behavioral issues and poor performance in ridden sport horses diagnosed with primary back pain (4). Together, these findings indicate that load-related and axial musculoskeletal disorders are highly prevalent, and we conclude that they are frequently interconnected rather than being isolated clinical entities.

Despite these documented associations, an integrative systemic framework linking structural organization, locomotor coordination, and progressive pathology in the ridden horse has not yet been explicitly formulated. Although biomechanical models of equine locomotion such as described by Back & Clayton (5) or Clayton & Hobbs (6), biotensegral models of structural organization as for example published by Levin (7) or Scarr et al. (8, 9), and coordination-dynamics models of movement organization as reported by Kelso (10) have addressed specific aspects of load transfer and structural coupling, these perspectives have largely developed in parallel. A unifying framework specifically addressing progressive systemic change in the ridden horse remains to be articulated.

This situation is addressed herein by proposing a precise conceptual framework that bridges theory and practice and offers scientifically consistent vocabulary for veterinarians, researchers, practitioners, and horse owners. Our hypotheses and theoretical framework support an integrative understanding of equine movement, structure, and self-organization by defining the systemic relationships underlying diverse pathological patterns of the equine musculoskeletal system. They provide a common foundation for describing and interpreting functional anatomy and strengthen scientific discourse, research, and clinical practice.

We introduce the term Progressive Structural and Functional Change (PSF; suggested translations in Appendix) to describe the underlying systemic process that affects the equine musculoskeletal system. PSF comprises two trajectories: Progressive Structural and Functional Loss (PSF^−^), characterized by progressive maladaptive reorganization of movement coordination and load distribution; and Progressive Structural and Functional Gain (PSF^+^), which emerges when maladaptive cycles are reversed and physiological function is re-established, initiating a self-stabilizing process of continuous functional improvement. The proposed terminology does not replace established diagnoses or terms (see Supplementary material) but integrates them into a broader systemic framework.

In addition to the published associations described above, our observations from applied equine contexts suggest further systemic regularities. Ten horses were selected for retrospectively documented and analyzed case studies (Supplementary material), representing a heterogeneous group (Table 1).

**Table 1:** Sample characteristics of the retrospectively documented case study horses (January 2020–December 2025).

Parameter	Value
Total sample size	10
Median age^a^	12.5 years
Age range^a^	7–17 years
Sex distribution	80% geldings, 20% mares
Breed distribution	40% Warmblood, 20% German Riding Pony, 40% Other (Thoroughbred, Arabian, Murgese, Quarter Horse)
Discipline background^b^	40% English Riding – Leisure, 20% English Riding – Competitive, 10% Steeplechase, 10% Endurance, 10% TREC, 10% Western Reining
Geographic context	German speaking region (90% Germany, 10% Switzerland)

Inclusion criteria were completeness of documentation and consistent implementation of the PSF^+^/FIT framework by the respective corresponding owners.

^a^Age refers to the horse's age at the end of the documentation period (2025).

^b^Discipline background corresponds to primary training discipline prior to implementation of the PSF^+^ framework.

The cases showed consistent physiological and pathological movement patterns, as well as key control nodes, which are hypothesized to influence whether the system evolves toward functional decline or adaptive functional reorganization.

The framework may explain the frequent co-occurrence of seemingly unrelated symptoms (see Supplementary material) and the sometimes limited, short-term effectiveness of isolated local interventions.

This article is conceived as a Hypothesis and Theory contribution rather than as a systematic review or controlled empirical study. The proposed PSF framework is derived from exploratory field observations in equine training and rehabilitation settings, and from a question-driven synthesis of relevant movement organization concepts. The included case material serves to exemplify recurring functional patterns and does not represent a statistically controlled or population-based sample. Accordingly, the purpose of this manuscript is to articulate a coherent systemic hypothesis and to define its conceptual structure, thereby enabling future controlled validation and operationalization.

By combining theoretical modeling with empirical observation, the PSF framework generates testable hypotheses for future research and provides a coherent foundation for clinical and therapeutic applications. The documented case studies and developments therein emphasize the need for systematic investigation and interdisciplinary validation of the proposed framework and its associated hypotheses.

## 2 From practice to theory: development of a conceptual framework for systemic equine (patho-)physiology

### 2.1 Terminological background

In publications of the English-speaking veterinary and equine fields, various descriptive terms have been used to characterize the broad spectrum of locomotor dysfunctions and performance limitations frequently observed in modern horses. The most common include Topline Syndrome, Poor Posture Syndrome, Myofascial Dysfunction, and Thoracolumbar Dysfunction.

Although widespread, these terms remain primarily descriptive and lack a coherent systemic framework (bibliographic search strategy see Supplementary material). Topline Syndrome or Topline Dysfunction, which probably originated within the equine feed and training industries, focuses on the visible contour of the back and neck rather than on functional integrity or load-bearing capacity. According to our knowledge, the term appears predominantly in commercial and non-peer-reviewed contexts. In scientific literature, Volesky names Topline Syndrome in 2020 (11), whereas Ursini explicitly proposes Topline Dysfunction in 2024 as a newer descriptive term (12). This indicates that the overall concept of issues in the topline has not yet gained recognition as a standard veterinary diagnosis. Poor Posture Syndrome (13), adapted from human physiotherapy, refers to postural appearance without addressing causal mechanisms. Myofascial Dysfunction (14), derived from human medicine, describes localized soft-tissue impairment assumed to be reversible through manual therapy, yet in equine contexts it oversimplifies the complex systemic interactions at play. Thoracolumbar Dysfunction (15) further restricts the issue to a single anatomical region, neglecting the integrated organization of equine biomechanics.

In German-speaking contexts, various terms have been used to describe the same phenomenon, most prominently “Trageerschöpfung” (carrying fatigue) (16) and “Trageschwäche” (carrying weakness) (17). The former incorrectly attributes the observed dysfunctions to fatigue, while the latter introduces semantic vagueness that may pathologize horses that are just untrained but functionally normal. Both terms, likewise, their previously named English counterparts such as Topline Syndrome or Myofascial Dysfunction, remain descriptive labels that do not articulate the systemic reorganization processes underlying the observed phenomena.

The absence of integrative terminology complicates diagnostic classification and limits the ability to relate seemingly unrelated findings within a coherent explanatory structure. Within current veterinary practice, clinical attention is necessarily directed toward the identification and treatment of local structural lesions, which may leave underlying functional organization of the locomotor system insufficiently addressed.

### 2.2 System-theoretical foundations for the PSF framework

Within systems theory, biological organisms are understood as open, hierarchically organized systems in which global functional states emerge from the interaction of multiple interconnected subsystems rather than from isolated local mechanisms (18). In this context, “systemic” refers to properties of the organization of the whole rather than to single anatomical structures.

Dynamic systems theory conceptualizes coordinated movement as a process of self-organization in which stable patterns (attractor states) arise from component interactions under specific constraints (10). Transitions between such patterns may occur when control parameters cross critical thresholds, resulting in qualitative reorganizations of coordination dynamics.

In analogy to this concept, the present framework uses the term control node to describe anatomically localized but system-relevant parameters whose configuration may influence global organizational states. The specific application of this concept within the PSF framework is outlined in the following section.

### 2.3 Progressive structural and functional change: definition and conceptual framework

Rather than being attributable to single external triggers, the systemic origins of conditions previously described under terms such as Topline Syndrome, Poor Posture Syndrome, or Myofascial Dysfunction appear to lie in widespread structural alterations and changes in functional organization within the equine body. We assume these manifestations to be closely associated with reduced motor coordination and proprioceptive control, collectively impairing the horse's ability to maintain adaptive movement under load.

Our field observations have revealed recurring combinations of movement and postural patterns that serve as central components of a broader systemic process. Within the proposed framework of PSF, these observations can be understood as expressions of two possible trajectories: PSF^−^ and PSF^+^, which denote a self-amplifying pattern of structural and functional loss and a self-amplifying pattern of physiological recovery and improving functional organization, respectively.

Within the proposed framework, these trajectories are interpreted as extending beyond explanations based solely on fatigue, isolated muscular dysfunction, or superficial conformational features, but rather reflecting a systemic process affecting the musculoskeletal and myofascial components of the locomotor system. This process is hypothesized to involve altered load distribution, modified integration of external forces, and changes in functional coordination.

Potentially relevant modulating factors include training approaches, activity levels, management and environmental conditions, hoof conformation and trimming practices, equipment fit, and the horse's overall health status and physiological condition. Depending on the way these factors interact with the horse's functional organization, they may modulate the system toward PSF^−^ by fostering a negative cycle of maladaptive load distribution and reduced locomotor efficiency. Under more favorable conditions, the same factors can support a systemic shift toward PSF^+^ and promote a positive cycle by enabling improved coordination, functional integration of external forces, and physiological resilience.

Framing these phenomena within the PSF concept allows previously isolated findings—such as recurrent lameness, postural asymmetries, or unexplained behavioral issues (see Supplementary material)—to be interpreted as expressions of a shared systemic mechanism rather than unrelated local disorders. This provides a conceptual foundation for integrating clinical observations, identifying meaningful control nodes as described in the following (2.4.2), and prioritizing interventions that support systemic self-organization.

The PSF framework evolved from field observations and is intended to function as a heuristic model designed to organize existing observations into a coherent systemic structure and to generate testable hypotheses. It is not intended as a closed explanatory theory, but as an organizational framework that structures recurring observations and enables their empirical examination.

### 2.4 Field observations and the mechanism of functional inversion

#### 2.4.1 Field observations

Across the documented cases and field observations, horses exhibiting recurring external features such as a steeply tilted pelvis, lordosis, retracted forelimbs with narrow, high-heeled fore hooves, camped-under hindlimbs with underrun heels, and reduced epaxial musculature consistently showed characteristic movement restrictions and a prevalence of load-related pathologies. These recurring associations formed the observational basis from which functional markers of the PSF^−^ trajectory were derived, which will be presented in section 2.6. This presentation contrasts with the classical description of a riding horse, which is characterized by a harmonious topline, vertical cannon bones, and well-proportioned, balanced hooves.

The underlying material for the hypotheses and theoretical framework presented in this paper were collected from structured questionnaires completed by participants in equine movement and functional anatomy programs (not published, collected by Maren Diehl, 2020–2025). While this material is not derived from a controlled or population-based study and does not provide statistical evidence, the recurrence of similar findings across independent contexts shows patterns compatible with the proposed PSF framework. Longitudinal field observations collected by the authors over several decades indicate that the above-named external features that are characteristic of PSF^−^ tend to progress over time and are accompanied by a typical set of physical and behavioral signs (Supplementary material). Conditions commonly reported in association with these patterns include musculoskeletal, visceral and neurological findings such as osteoarthritis, tendinopathies, and gait abnormalities such as stumbling and phase shifts. In many cases, these physical findings are accompanied by behavioral and dysfunctional manifestations, including shying, head shaking and lameness without any findings, which were interpreted as a reduction in functional awareness and coordinated movement control of the horse. These patterns have been observed across breeds, ages, sexes, training levels, riding styles, disciplines and management systems, suggesting that the phenomenon is systemic in nature rather than breed-, management-, or discipline-specific.

In attempts to alleviate these conditions, horse owners frequently invest substantial time and financial resources in countermeasures, including physiotherapy, osteopathy, chiropractic, diet, changes in training methods or equipment, and specialized farriery. However, in many cases these interventions were reported to produce only temporary improvement, and prior to the program participation with Maren Diehl, several of the horses selected for the case studies had been classified as non-rideable or treatment-resistant (see Supplementary material).

#### 2.4.2 Development of PSF^−^: the mechanism of functional inversion

Field observations suggest a recurring pattern that may underlie the development of what we define as PSF^−^, which we conceptualize as being associated with a process herein termed as functional inversion. Within the proposed framework, this mechanism is interpreted as a systemic reversal of physiological roles within the locomotor system. It is hypothesized that, prior to visible conformational changes or secondary pathologies, functional inversion alters the direction and transmission of forces throughout the body. In this interpretation, structures typically contributing to stabilization become destabilizing; movements normally associated with efficiency become mechanically less economical; and external forces that could be integrated efficiently instead require compensatory resistance.

Unlike local compensation or regional maladaptation, functional inversion affects the entire locomotor pattern, transforming adaptive dynamics into self-reinforcing maladaptive cycles. This process is hypothesized to manifest in reduced mechanical efficiency and increased stress on the horse's body.

In practical terms, the process can be conceptualized as follows:

- Within the proposed framework, the horse develops a steep pelvic orientation, which is hypothesized to be causally linked to training methods, pain, or environmental constraints.
- This posture shifts hindlimb function toward a predominantly braking role, which may increase caudally directed tensile loading of the spine.
- The forehand responds to these altered load directions with comparatively greater cranially directed propulsive effort, a pattern interpreted as being associated with a lowered thorax.
- The limbs are displaced further beneath the body; the fore hooves show increased toe loading, whereas the hind hooves experience comparatively increased heel loading.
- With continued repetition of such load distribution patterns, hoof morphology gradually adapts toward relatively steeper fore hooves and flatter hind hooves.
- In conclusion, within this framework, functional inversion is associated with structural remodeling of tissues that are not primarily adapted to sustain such load directions.

The process thus is conceptualized as self-reinforcing: the longer altered force patterns persist, the more the horse's morphology shifts toward what is defined here as a maladaptive state.

Owing to the interconnectivity of the elements described above, the framework further proposes that different initiating factors converge on a similar systemic pattern, although this assumption requires empirical validation.

**Figure 1:**
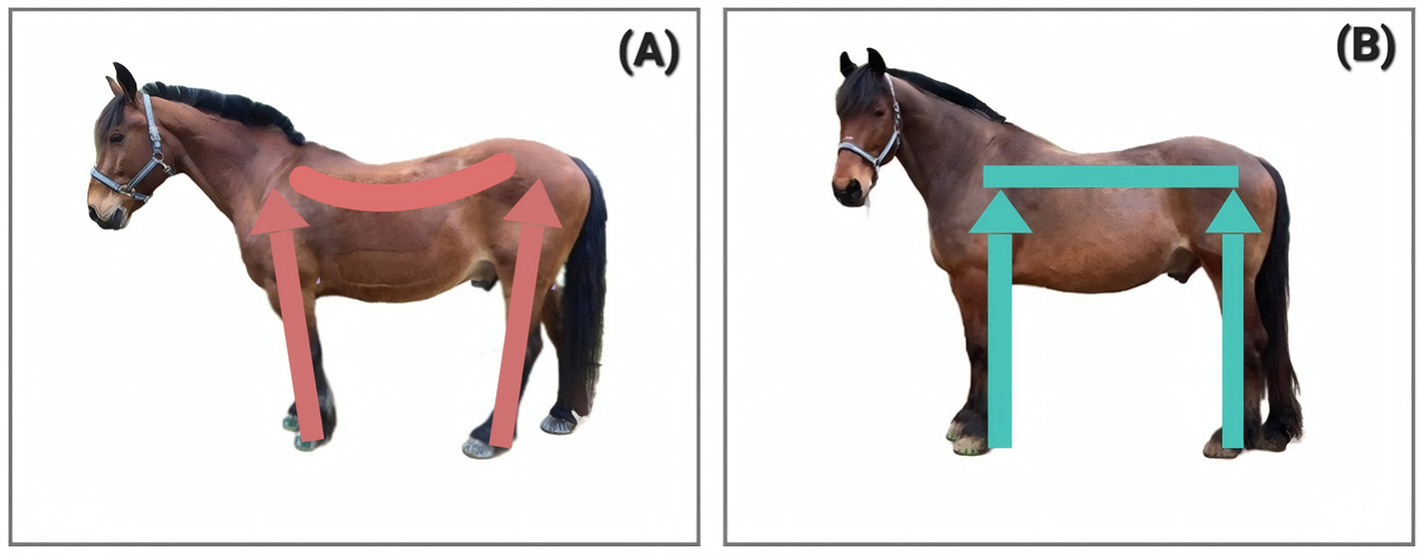
A horse in PSF^−^ **(A)** and the same horse one year later and in PSF^+^ **(B)**. This 19/20-year-old gelding was trained with riding and driving solely by the owner (No case study for this specific horse available, these pictures serve to visualize the processes).

A functional reversion from PSF^−^ toward a physiological, self-stabilizing cycle of PSF^+^ as displayed in Figure 1 requires the removal of the initiating cause of unphysiological pelvic steepness and support for structural reorganization through appropriate hoof management and targeted functional training. Our empirical observations suggest that the lumbosacral joint (LSJ) and the hooves act as primary control nodes within this system, while horizontal forward tension toward the bit serves as a functional catalyst for re-establishing coordinated, physiological movement.

##### 2.4.2.1 The LSJ

The LSJ plays a pivotal role in coordinating axial motion and hindquarter engagement during locomotion. Given its articulation between the lumbar spine and the sacrum, it functions as a hinge-like regulator of pelvic orientation and thereby governs the direction and quality of force transfer through the body (19).

In many traditional training systems, a steep or upright pelvic orientation is considered beneficial for collection and carrying capacity. However, if this posture is created without adaptive flexion of the lumbar spine, it requires an open LSJ, increasing the angle between the spinous processes of the last lumbar vertebra (L6) and the sacrum (S1). The larger this angle becomes, the steeper the pelvis appears and the more the hindlimbs are positioned beneath the body, both standing and in motion.

In horses presenting with a persistently open LSJ during the stance phase, reduced hindlimb flexion and mid-stance engagement have been observed across disciplines and training systems. The hip, stifle, and hock move into increased extension while the fetlock takes disproportionate load, shifting the hindlimbs toward a predominantly braking rather than propulsive function. This altered load-transfer strategy is proposed to be associated with secondary changes in limb kinematics and axial posture, including elevation of the croup relative to the withers and a progressively lowered thorax, both features associated with increased risk of lameness and back pain (20).

Within the present structural-physical framework, the LSJ represents a potential point of axial discontinuity. In a persistently open configuration, force transmission through the axial system may be altered (more detailed explanation see 2.4.2.4). Mechanically, an open LSJ compromises the efficiency of force transfer between the hindquarters and the trunk (19). The horse's spine undergoes cycles of flexion and extension during locomotion (6) and failure of the LSJ to achieve functional closure during load transfer is expected to increase tensile loading within the axial system. In this context, the balance between compression and tension may become altered, potentially resulting in reduced force distribution efficiency and contributing to what is defined herein as PSF^−^. This condition, conceptualized in this framework as functional inversion, is interpreted from the perspective of biotensegral system organization, which conceptualizes biological structures as hierarchically organized tension–compression systems (7, 8). Within the present framework, such a shift is interpreted as compromising structural coherence across the locomotor chain and increasing mechanical demands, thereby potentially predisposing the system to secondary pathology.

**Figure 2:**
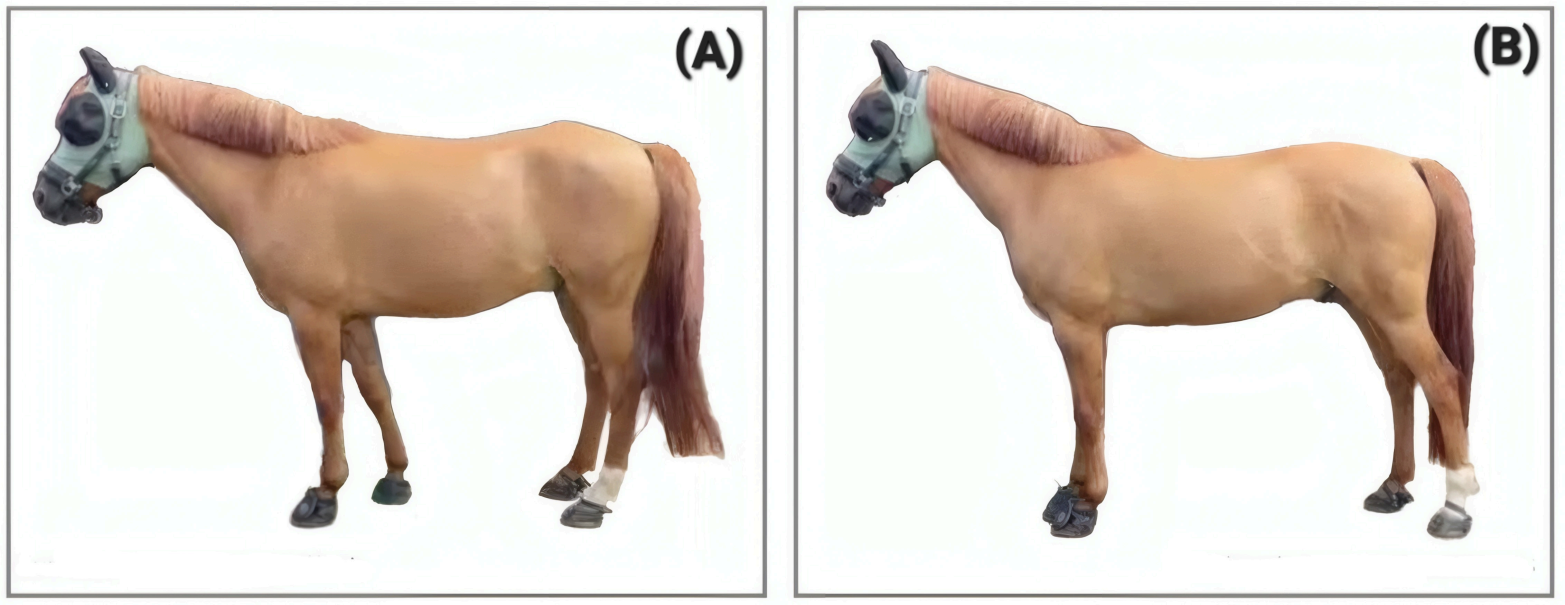
11-year-old Gelding “Case 1” at the very beginning of PSF^+^, both pictures taken within minutes from each other. **(A)** shows the horse's self-chosen posture; **(B)** shows the posture after the LSJ has been closed via manual manipulation of trigger-points on the croup. Case study 1 is documented in the Supplementary material.

Accordingly, inversion of force directions is proposed as a central mechanism within PSF. The LSJ as a control node can influence not only the spatial relationship between pelvis and spine (Figure 2), but also aspects of temporal coordination, including the timing of force peaks, stance duration, and stride symmetry. Consequently, in this context, the LSJ therefore functions both as an early indicator for a beginning PSF^−^ trajectory, and a potential therapeutic gateway for facilitating a transition toward PSF^+^ movement organization.

##### 2.4.2.2 Phase shifts

Functional inversion, resulting from altered force directions within the equine body, is proposed to produce a phase shift in locomotor coordination, which manifests in timing anomalies between the stance and swing phases of the fore- and hindlimbs. If so, the first half of the stance phase of the forehand becomes shortened while the second half is prolonged; in the hindquarters, the opposite is to be expected, with an extended initial and a shortened terminal stance phase.

**Figure 3:**
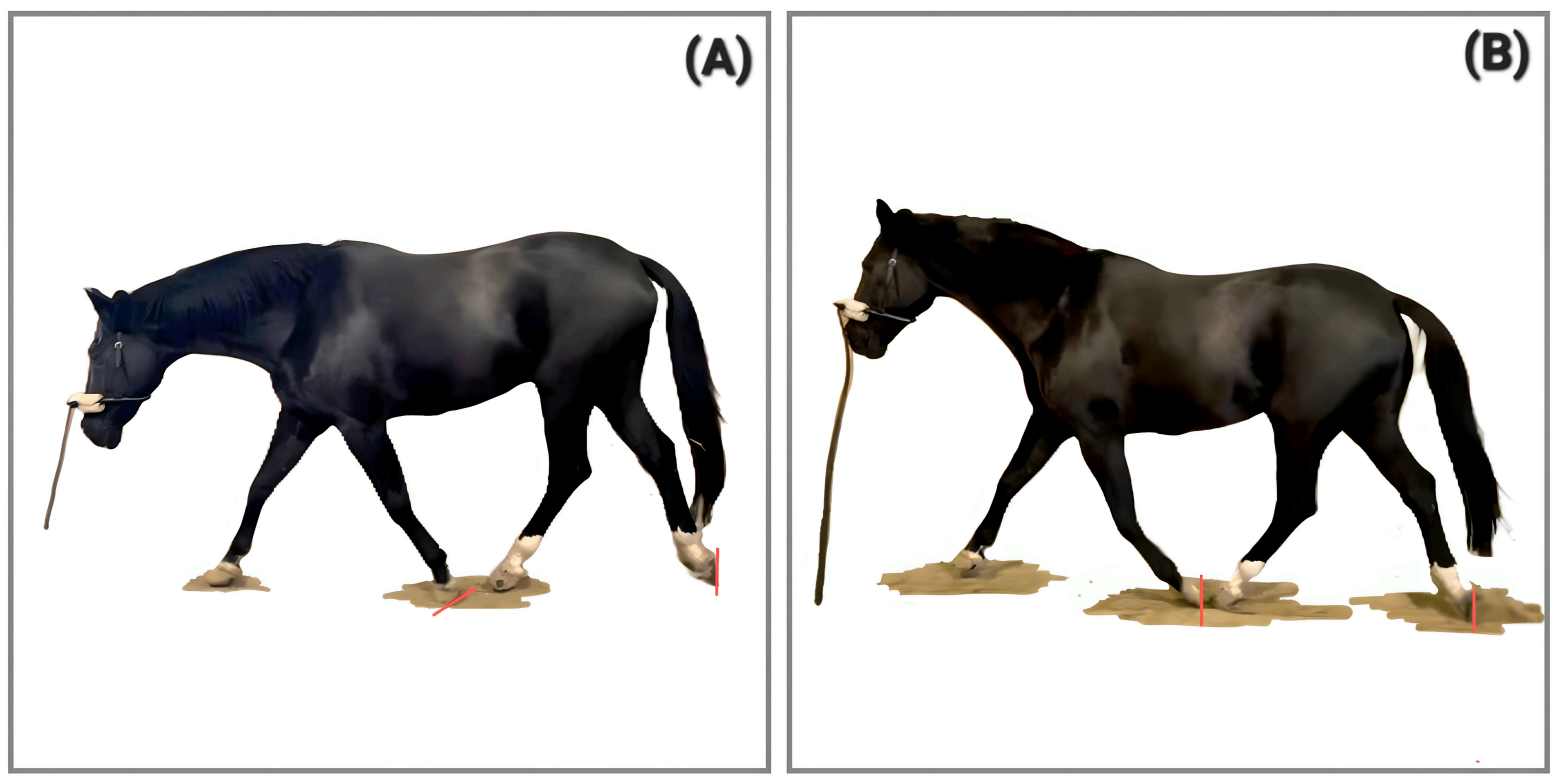
Phase Shift presented by a 19-year-old gelding (no case study, pictures for illustration). **(A)** shows the lagging left forelimb in forward and down orientation, **(B)** shows the synchronized diagonals some minutes later in the same training session on the first day of his PSF^+^ journey.

**Figure 4:**
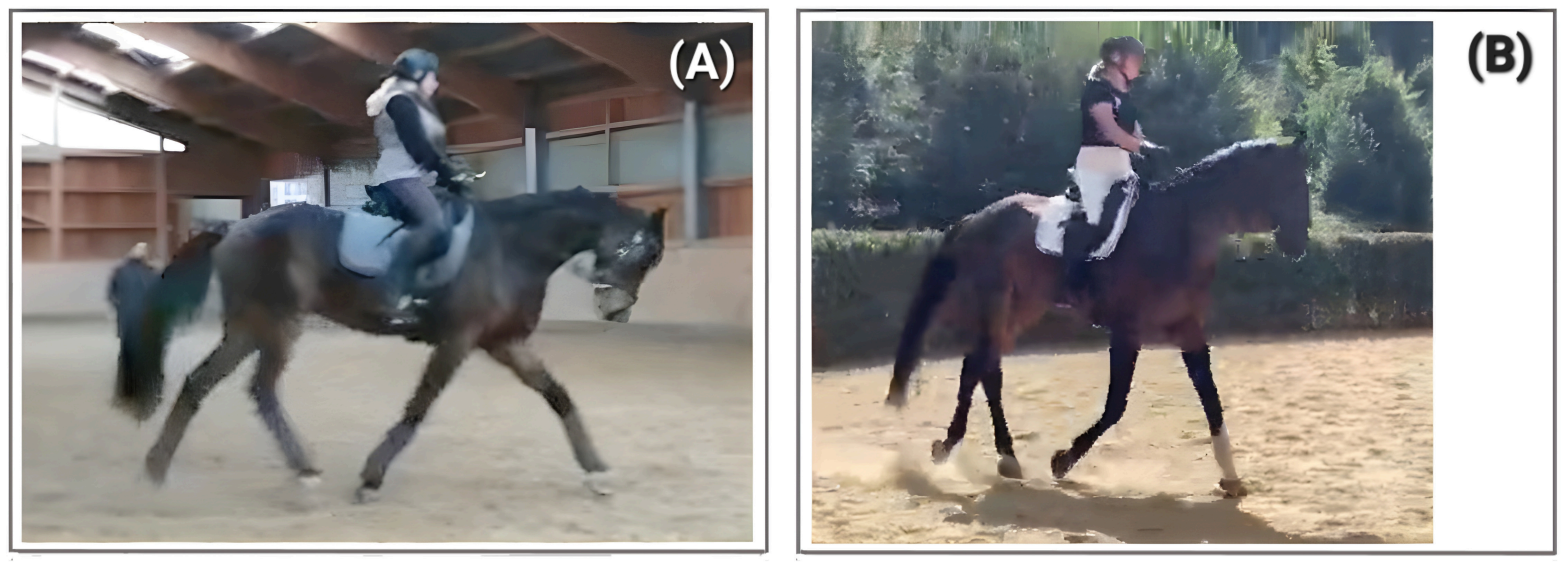
Phase shift presented by “Case 2” at 4 **(A)** and 7 **(B)** years of age. In **(A)** the right front hoof is still on the ground while the hind hoof has already been lifted. In **(B)** the right fore and left hind move simultaneously and the phase shift has vanished, which is attributed to the change in training (picture taken two years after the introduction of horizontal tension toward the bit). Case 2 is documented in the Supplementary material.

The shift is most evident in trot at the end of the stance phase (Figures 3, 4), when the forelimb remains grounded and under load after the diagonal hindlimb has already left the ground. In many such cases, the suspension phase becomes markedly reduced. This pattern is widely observable across riding disciplines and levels of training.

In biomechanical literature, larger hindlimb dissociation (hindlimb contacting the ground before the diagonal forelimb) has been described as being associated with specific advanced dressage moves, as well as with increased speed in trot, where it is interpreted as contributing to trunk pitch control and redistribution of mass (21). Within the present framework, we focus on the timing of hooves leaving the ground and if a temporal dissociation occurs together with prolonged forelimb stance, reduced suspension, and diminished hindlimb flexion, it is interpreted not as a sign of increased carrying capacity, but as part of a functionally inverted load-transfer strategy characteristic of PSF^−^.

##### 2.4.2.3 Hooves

The hooves can be understood both as a physical manifestation of the horse's movement patterns and a reflection of the principles guiding hoof care. Multiple studies have shown that the orientation of the distal phalanx (P3) is correlated with external characteristics of the hoof capsule (22, 23). Furthermore, hoof trimming has been shown to have an immediate effect on the conformation of the appendicular skeleton of horses (24), and hoof conformation plays a crucial role in limb biomechanics and can consequently prevent or predispose to injury (25).

**Figure 5:**
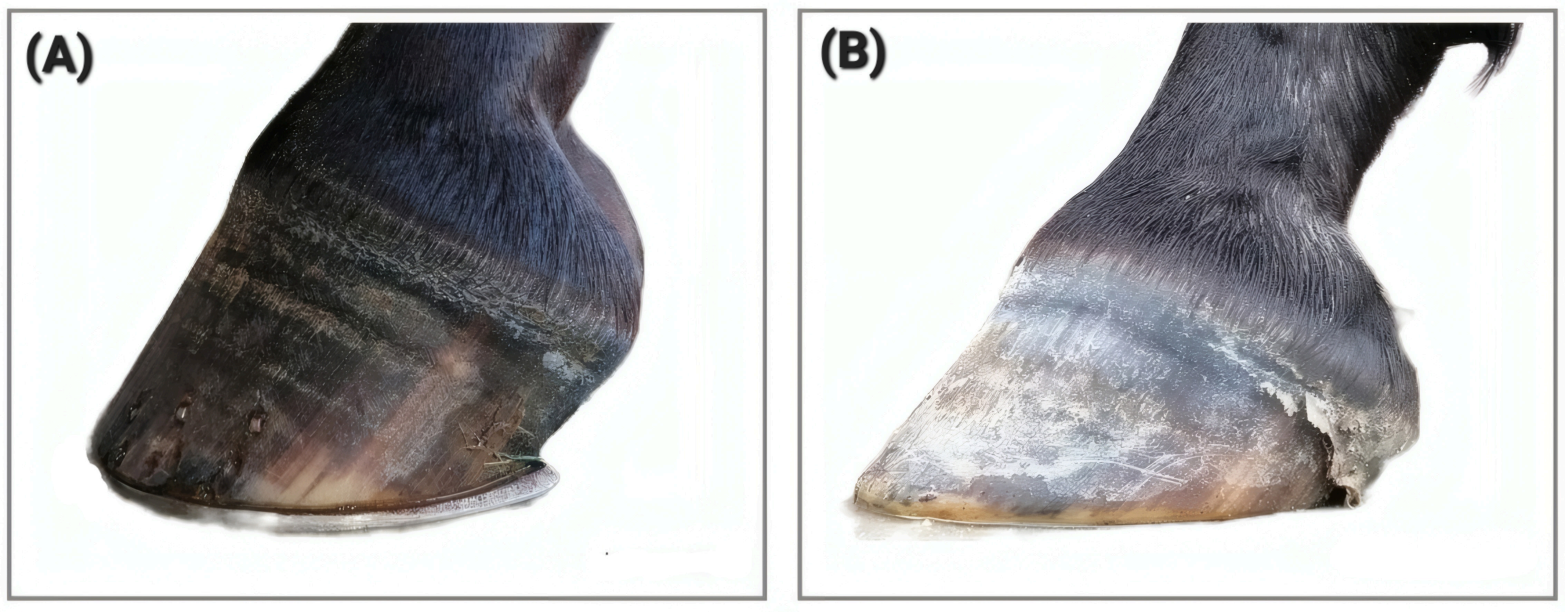
Left front hoof of “Case 7”: **(A)** shows the hoof in January 2025, **(B)** in September 2025. The lower hoof in **(B)** matches the new physiological motion pattern according to PSF^+^, although the hoof is not yet in its final shape. Case 7 is documented in the Supplementary material.

In agreement with the correlations found in the studies cited above (although we come to a different conclusion concerning desirable hoof morphology), we see progressive remodeling during PSF^−^: In most cases, the dorsal hoof wall angle of the fore hooves gradually increases, while that of the hind hooves decreases, accompanied by inverse changes in coronary band angles (Figure 5). Steeper fore hooves—characterized by higher dorsal hoof wall angle and lower coronary band angles—are associated with a steeper dorsal aspect of P3 and an increasing palmar angle relative to the ground, whereas the hind hooves tend to develop decreasing plantar angles. These reciprocal changes reflect a systemic reorganization of mechanical relationships within the locomotor apparatus. Rather than representing a mere consequence of imbalance, they constitute both a contributing factor and the morphological imprint of the proposed underlying systemic dysregulation. The altered hoof geometry reinforces the functionally inverted movement pattern typical of PSF^−^, as each step further consolidates the non-physiological load distribution between forehand and hindquarters.

In horses presenting with a persistently open LSJ, such remodeling seems to be particularly pronounced according to our observations. A progressive steepening of the fore hooves and flattening of the hind hooves have been identified as risk factors for lameness (25), particularly in association with hock and proximal suspensory ligament disorders (26). Consequently, we conclude that hoof management may influence not only the progression or potential reversal of PSF^−^, but also the configuration of the hoof-ground interface. This interface is central to restoring functional integrity within the system.

##### 2.4.2.4 Active horizontal tension toward the bit – the pull

**Figure 6:**
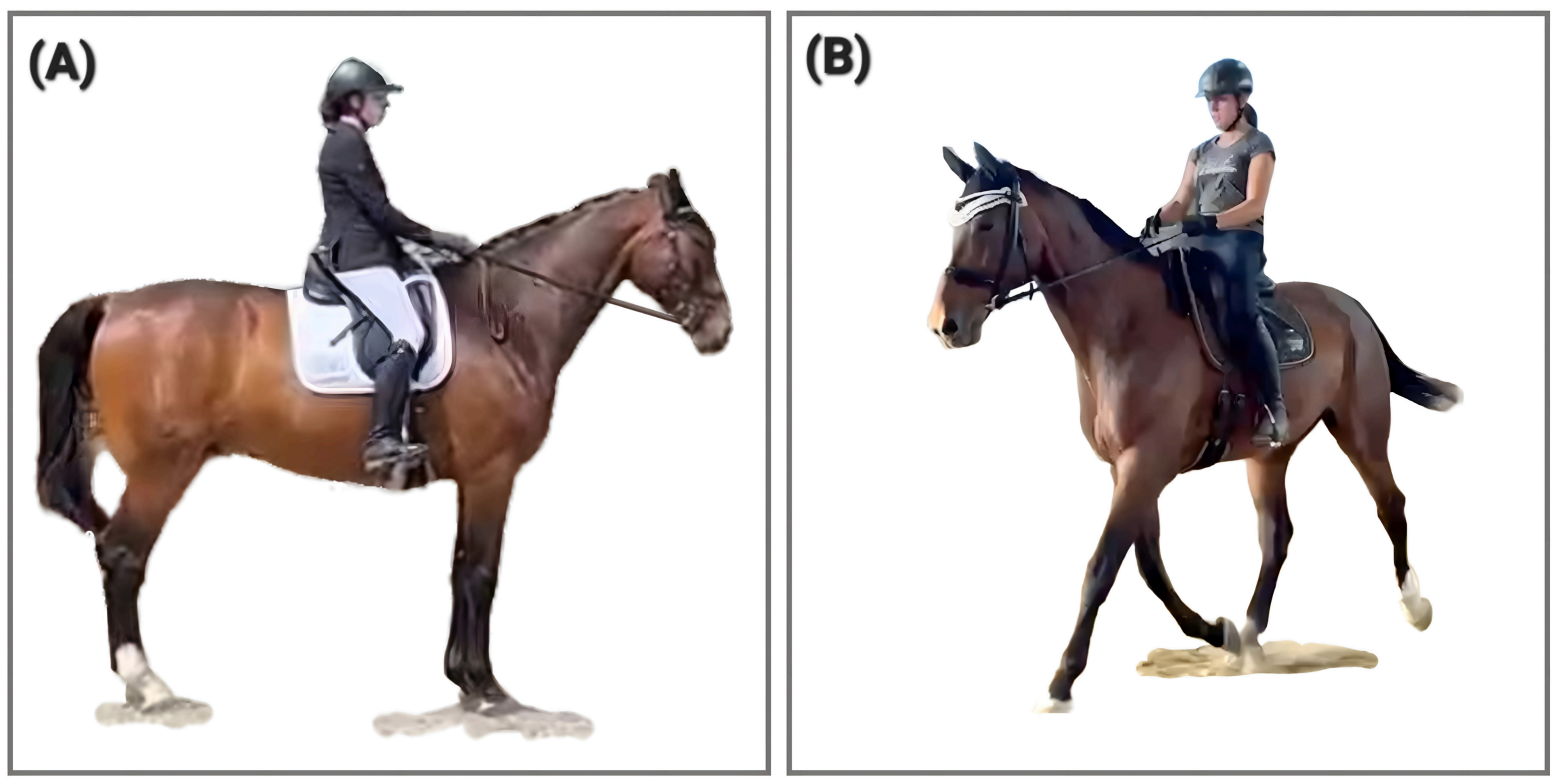
“Case 3” in summer 2025 at 13 years of age showing an active horizontal tension toward the bit. In both panels, horse and rider are showing a relaxed, yet active tension and connection. Note the overall smooth muscles of the horse, the poll being the highest point, and the relaxed forearms and hands of the rider. Case 3 is documented in the Supplementary material.

The core training objective for the horses in the documented cases was the establishment of active horizontal tension toward the bit. This is an intentional, forward-directed pull initiated by the horse itself rather than imposed by the rider (Figure 6). This action generates a continuous tensile pathway between the horse's tongue and the rider's body, promoting a functional, self-organized redistribution of forces through the axial and appendicular systems.

In most cases, a more positive effect was observed with a bar or snaffle bit compared to bitless bridles. The authors attribute this to the functional role of the tongue: As a strong muscle and an integral part of the myofascial system, the tongue is mechanically connected to the deep myofascial chains representing the core structures of the horse's body (27).

The active horizontal tension toward the bit can be summarized in a simple instruction: allow the horse to pull. The horse is expected to actively move forward into the bit and to generate the forward-directed force itself, rather than being positioned or held there by the rider.

This action may be compared to a person carrying a heavy backpack who hooks the thumbs into the shoulder straps and actively presses forward in the direction of movement. By doing so, the load no longer passively hangs but becomes dynamically stabilized against the body. The contact pressure increases, yet the load becomes more stable and easier to carry because it is actively organized rather than passively borne.

Transferred to the horse–rider system, active forward engagement into the bit is hypothesized to organize the body along its longitudinal axis as a continuous functional unit during movement and transitions. This corresponds to what will later be defined as impact stability.

**Figure 7:**
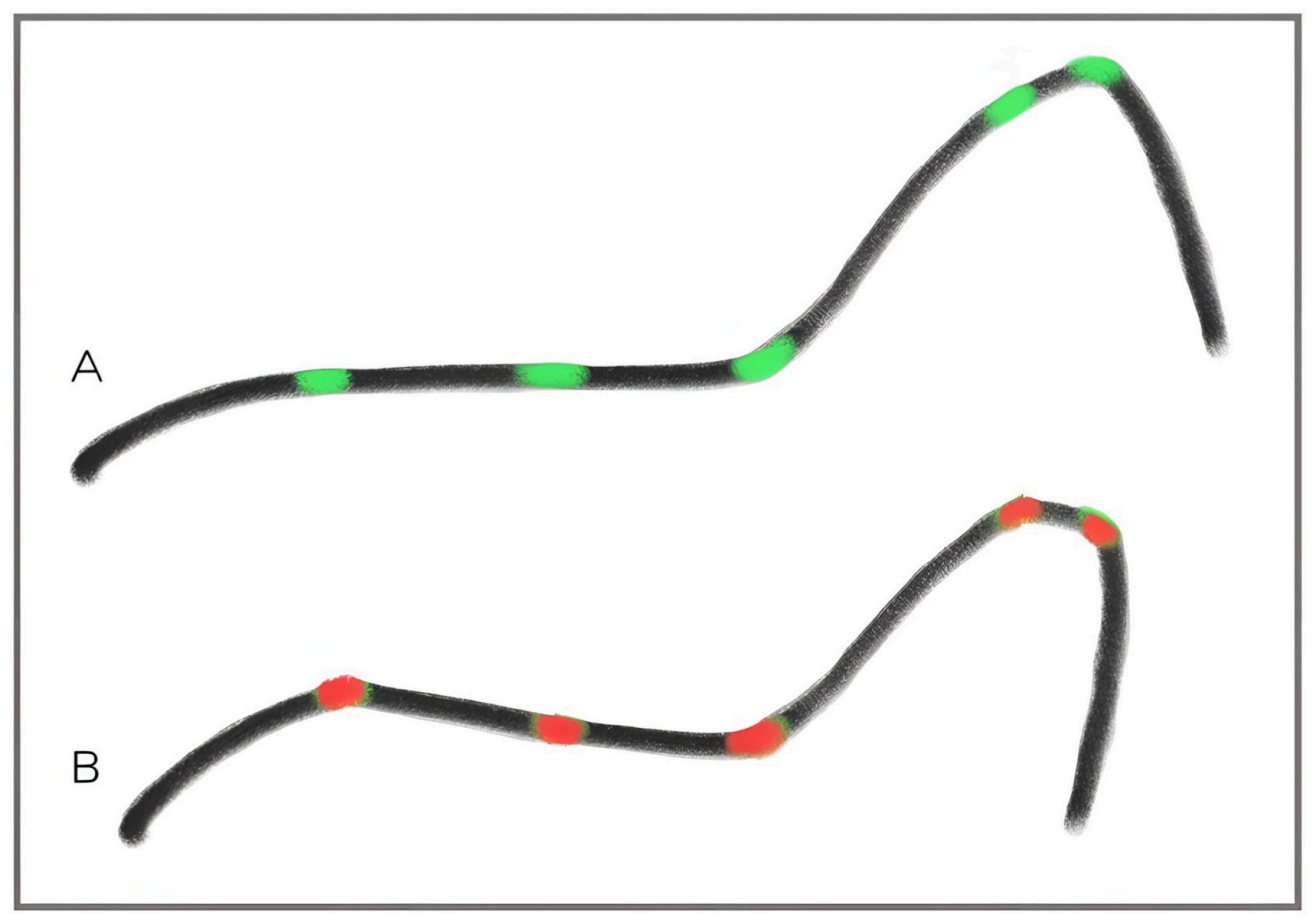
A schematic illustration of the “steel-rod-like” axial coherence in PSF^+^ **(A)** compared to segmental discontinuity in PSF^−^ **(B)**. (Colored dots from left to right: lumbosacral junction, anticlinal vertebra, cervicothoracic junction, C3, poll). The rods serve as a heuristic visualization of global load-transfer organization and perceived axial continuity, not as a biomechanical simulation or mechanical model.

We employ the biotensegral model of structural organization (9) to describe this horse-initiated pull as a task-dependent functional linkage of kinematic chains within the horse's body. This functional linkage is interpreted from our end as what has previously been described in the literature as a preferable riding sensation: Udo Bürger and Otto Zietzschmann (28) wrote that the desired stability in the horse felt like a steel rod extending from the bit to the hip joints, bent at the poll and at the cervicothoracic junction (Figure 7). Through this “rod”, rein action was assumed to be transmitted through an already stable structure to influence the joints of the hindquarters. The present framework focuses on how such a “steel-rod-like” functional quality can be established. We conclude that axial stability must emerge through the horse's active forward engagement in order to use the body in the by Bürger & Zietzschmann described manner, before any meaningful transmission of rein influence can occur.

### 2.5 Functional and interactive training

In the documented cases presented in this paper, the transition from PSF^−^ to PSF^+^ was explored using a simple interaction-based training approach referred to here as Functional and Interactive Training (FIT). The term was introduced retrospectively to provide conceptual clarity and to label the intervention that had evolved through practical efforts to restore systemic organization. The principles of FIT can be applied to any riding style or training method. Note that prior to any interaction, training clearance by a veterinarian should be obtained.

FIT is based on the premise that structural and functional restoration cannot be achieved through isolated therapeutic corrections alone, but emerges through coordinated, self-organized reintegration of movement across the whole system. Within the PSF framework, the body's inherent biotensegral organization is understood as capable of distributing forces efficiently, generating dynamic stability, and supporting self-organized movement patterns when appropriately engaged.

A systemic functional manifestation of this process is conceptualized as impact stability. In this context, impact stability refers to the transient capacity of the neuro-myofascial system to generate momentarily high stability under load, enabling the efficient absorption and redistribution of short, intense force impulses, particularly ground reaction forces (GRF). Applied to the PSF framework, impact stability is considered as a central factor in facilitating PSF^+^.

**Figure 8:**
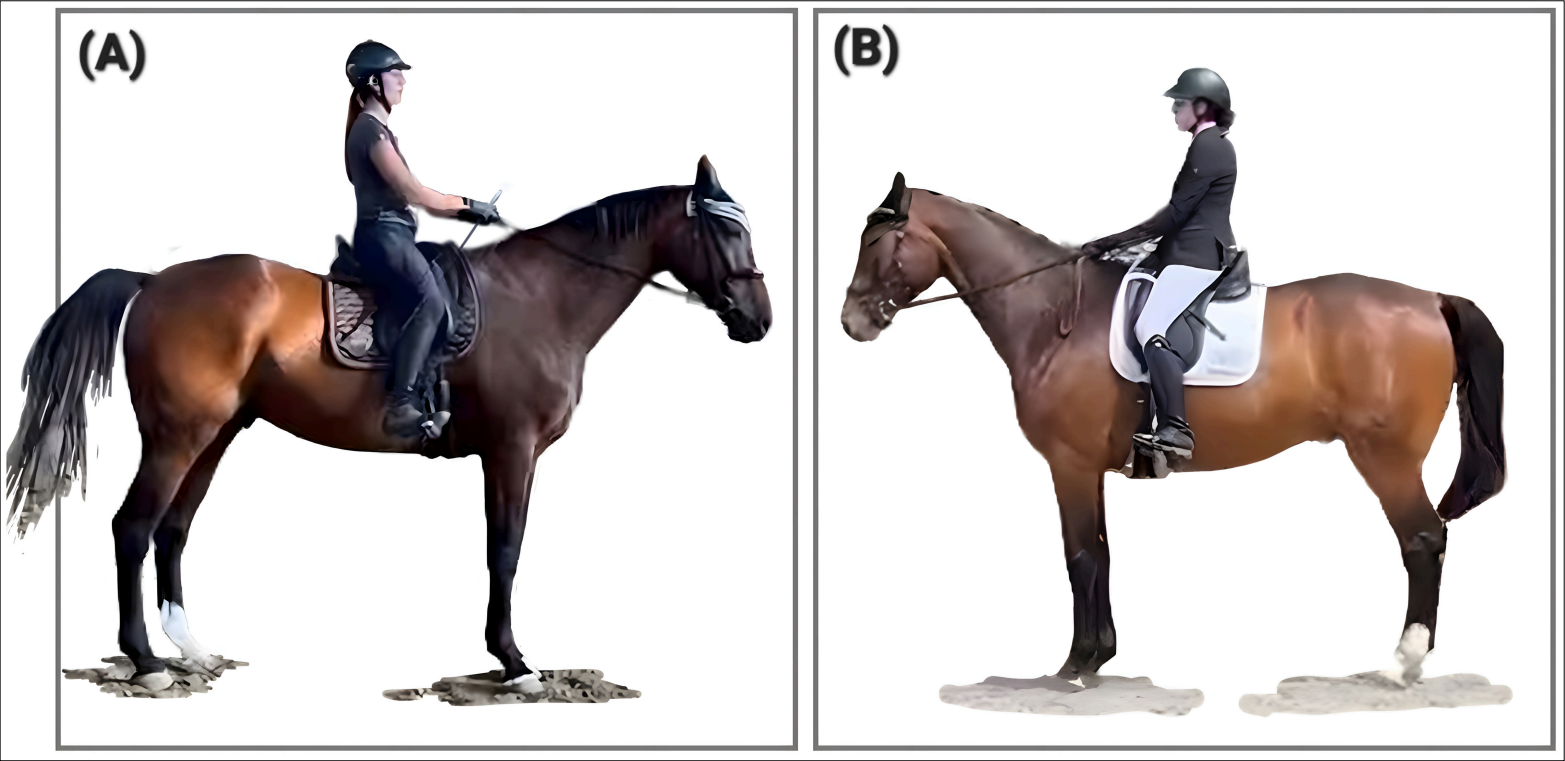
“Case 3” at 13 **(A)** and 12-years **(B)** of age exemplifying the impact of interaction with the environment in FIT: the same horse in **(A, B)** a comparable situation in two different environments. Each panel shows “Case 3” at the end of the “halt”, with forward energy in deceleration. **(A)** displays “Case 3” at the end of a relaxed training session in Summer 2025, **(B)** shows “Case 3” with overall higher agitation and alertness in a competition in spring 2024. Case 3 is documented in the Supplementary material.

FIT aims to promote impact stability by emphasizing the dynamic interaction between posture, load distribution, proprioception, and the use of external forces—such as GRF and gravity—as organizing stimuli for systemic re-stabilization. Within this framework, the interactive component of FIT describes how horse and rider may develop into a functional unit across varying environmental and situational demands (Figure 8), in alignment with the systemic principles of PSF. Although observable outcomes may vary depending on the developmental stage of each partner, the approach seeks to support a coordinated development of the horse–rider dyad.

The FIT approach integrates the previously described focal points of this paper:

- The LSJ as a structural and functional regulator of load transfer.
- Phase shifts within the stance phase in trot as indicators of functional inversion.
- Hoof care as a determinant of mechanical interface and proprioceptive feedback.
- The horse-initiated active horizontal tension toward the bit as a proposed biotensegral link between horse and rider within the shared tension network.

By combining these elements, FIT is intended to facilitate the re-establishment of physiological coordination patterns and supports the system's capacity for self-organization. In our interpretation, the process functions as an applied mechanism that can transform maladaptive feedback loops into constructive ones, thereby enabling a transition from PSF^−^ to PSF^+^.

In the presented case studies, FIT constituted the methodological context within which functional changes were observed. Horses which previously showed signs of PSF^−^ swiftly regained stability, symmetry, and postural self-organization after the initiation of FIT without additional therapeutic interventions reported during the documented period. Within the limits of these observations, FIT therefore serves as the operational bridge between the theoretical concept of functional inversion and its observable reversal in practice.

In the ten cases selected to illustrate the proposed framework, functional reversion opened a new pathway of sustainable positive development for horse owners, who themselves became the primary agents of change. As the horse's movement patterns reorganized, the need for therapeutic intervention decreased, while the quality of life for both horse and human improved.

**Figure 9:**
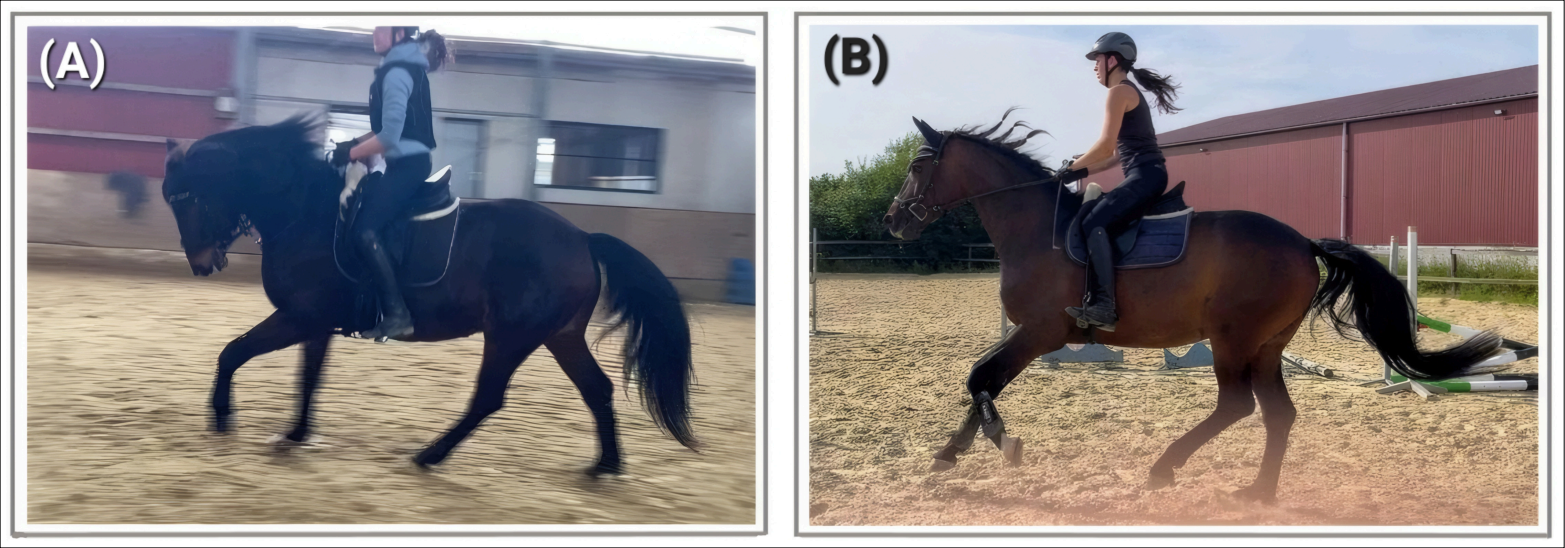
A gelding who tended to overreactions and shying in PSF^−^ at 8 years of age **(A)**, has shed his negative behaviors and transitions to PSF^+^ after the training had changed at 10 years of age **(B)**. The displayed horse (no case study) shows similar progression in PSF^+^ as described for Case Study 3 (Supplementary material).

**Figure 10:**
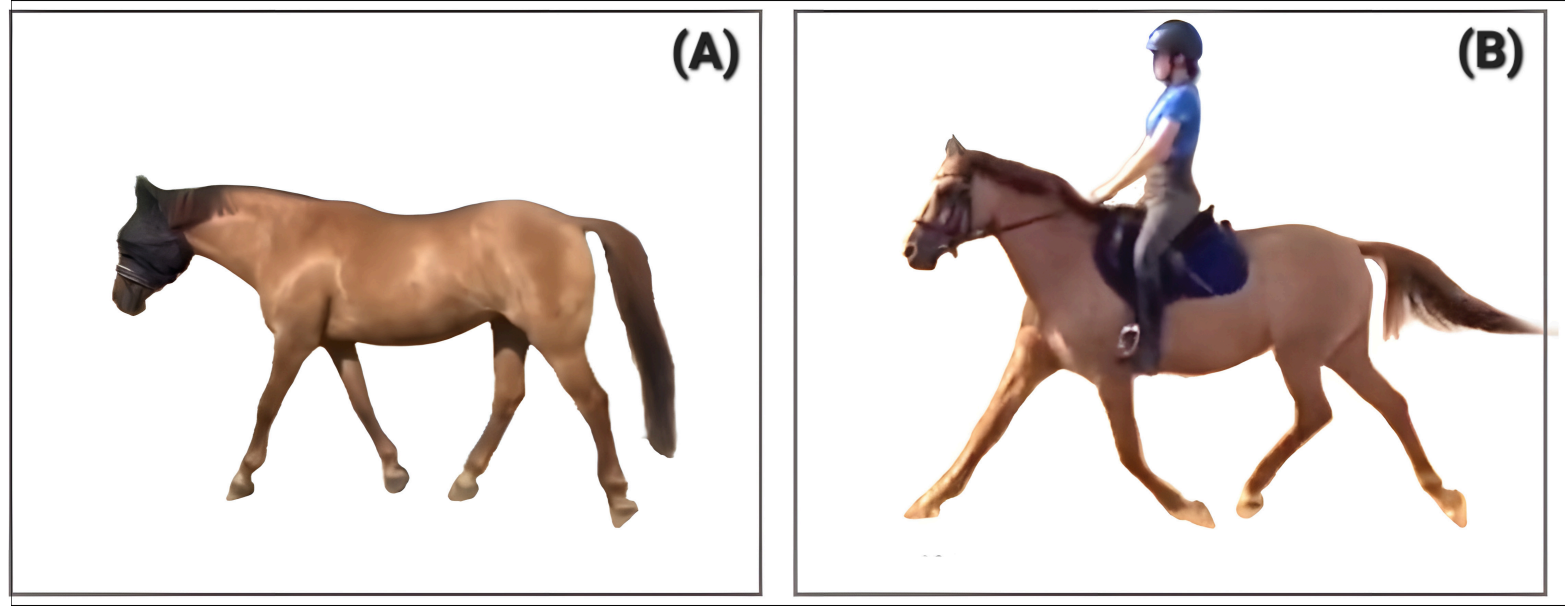
“Case 5” at 9 and 11 years of age showing the return to a functional state: **(A)** shows the mare in PSF^−^ at her low point in 2023, **(B)** in PSF^+^ in 2025. Note the change in the self-chosen range and speed of the horse from **(A)** to **(B)**. Case 5 is documented in the Supplementary material.

Across cases, the most frequently observed and reported changes included:

- Restoration of regular and rhythmically consistent stride patterns.
- The poll becoming the highest point of the horse's topline in movement (Figure 9).
- The facial profile remains in front of the vertical in movement (Figure 9).
- The horse's musculature gets smooth, even, and unobtrusive overall.
- Reduced spookiness and explosive behavior.
- Increased calmness combined with improved responsiveness (Figure 10).
- Reduced appearance or complete resolution of pre-existing asymmetries.

These changes are documented in detail in the selected case studies presented in the Supplementary material. Comparable developments have also been reported by horse owners participating in the online programs of Maren Diehl, based on structured questionnaires and non-standardized owner provided photo and video documentation. Although this material does not represent a statistically controlled sample and was not yet numerically analyzed, the recurrence of similar developments across the documented cases forms the primary basis for the proposed pattern within the applied context.

The practical results described above indicate that PSF^−^ may represent a reversible process that can be transformed into PSF^+^ through targeted intervention. This transformation may, depending on the individual clinical context, involve conventional veterinary treatment. However, the cases presented here suggest that restoration of functional integrity centrally depends on a systemic approach addressing the horse's capacity for physiological self-organization.

### 2.6 Operationalization of PSF state markers

To enhance falsifiability and practical verifiability of the PSF framework, a limited set of visually assessable markers was defined that can be evaluated using standard smartphone video (≈30 fps). The focus was on reproducibility and accessibility rather than maximal technical precision, allowing independent replication without laboratory equipment. For the present purpose—identifying clearly observable timing offsets and postural patterns—higher frame rates may improve temporal resolution but are not required for the qualitative detection of the described phenomena.

In the documented case studies, these markers were assessed qualitatively based on owner-provided photo and video material. Quantitative thresholds were formulated subsequently and are presented as preliminary reference values intended for future empirical validation.

Table 2 summarizes the proposed static markers associated with a PSF^−^ and PSF^+^ trajectory, respectively. Lateral assessment of the horse's conformation is intended to serve as an initial screening target. In our observations, these markers rarely occurred in isolation but were frequently observed in combination.

**Table 2:** PSF static markers assessable via lateral photography.

PSF static marker	Marker characteristics in PSF^+^	Marker characteristics in PSF^−^
Cannon bones	Minimum of three mostly vertical cannon bones with each less than 2° divergence from the vertical	Two or more cannon bones diverting at least 2° from the vertical
LSJ	Neutral	Open
Heave line (ventrolateral abdominal groove)	Not visible	Visible
Relative height of withers and croup	Withers at the same height as the croup or higher	Croup higher than the withers
Hooves	Toe angle of fore lower than hind	Toe angle of fore higher than hind

Assessment protocol: the horse stands freely on firm, level ground. A full-body lateral photo is required, ideally including 2 m of surrounding space in each direction to minimize lens-induced distortions. Minimum camera distance: 4 m, recommended camera height: 1.20 m. This assessment enables an initial classification of postural organization under static conditions.

Table 3 summarizes the proposed dynamic markers used for movement-related classification within the PSF framework. These criteria focus on observable locomotor patterns during trot and are intended to serve as proxy indicators of systemic load organization as expressed in dynamic coordination.

**Table 3:** PSF dynamic markers assessable via lateral video recording.

PSF dynamic marker	Marker characteristics in PSF^+^	Marker characteristics in PSF^−^
Phase Dissociation - Synchrony of diagonal toes leaving the ground in trot	Diagonal toes leave the ground within the same video frame	Temporal dissociation of diagonal toes leaving the ground (>1 frame difference)
Poll as anatomical reference point	Poll is the highest anatomical point	Poll is not the highest anatomical point
Facial profile relative to vertical reference	Facial profile in or in front of the vertical	Facial profile behind the vertical
Vertical croup position with respect to stance-phase timing	Lowest vertical croup position at mid-stance	Lowest vertical croup position at end-stance
Fetlock	Vertical recoil begins at mid-stance	Vertical recoil at end-stance

A lateral video capturing the entire horse is required, ideally including approximately 2 m of surrounding space in each direction to minimize lens-induced distortion. The recording should include 2–4 consecutive trot strides. Minimum camera distance: 4 m. Recommended camera height: 1.20 m. Minimum frame rate: 30 fps (60 fps may improve temporal resolution). Optional vertical and horizontal reference lines may assist visual assessment. Recommended analysis thresholds: markers for PSF^+^ should be present in >95% of frames, markers for PSF^−^ in >5%.

For the purpose of conceptual classification within this framework, the presence of at least two static and two dynamic markers characteristic for PSF^−^ is proposed as indicative of a PSF^−^ state. For classifying as PSF^+^, all of the named functional static and dynamic markers characteristic for a PSF^+^ state are supposed to be present. A transitional phase from PSF^−^ toward PSF^+^ or the inverse process may be identified by longitudinal tracking. In the studied cases, individual PSF^−^ markers resolved within weeks to months, whereas hoof-related markers required longer time frames, depending on hoof management and individual growth rate.

The temporal frequency of repeated screening depends on the individual clinical and training context. Longitudinal tracking of PSF state markers may support both confirmation of classification and monitoring of functional change.

Where acute lameness, neurological deficits, or recent orthopedic interventions are present, classification should be interpreted with caution, as such factors may confound movement-based assessment.

## 3 Discussion

### 3.1 Conceptual differences between models of structural organization

The approach presented here is founded on the premise that living organisms do not move through muscle-driven levers but function according to an integrative, adaptive system of tensile and compressive forces. This structural organization principle called biotensegrity was first transferred from architecture to biology by Levin (7) and later expanded by Scarr (8) and others.

Classical lever-based models provide a simplified mechanical representation that may not capture the full complexity of motion in living systems. They assume fixed pressure points and linear force application that necessarily create joint compression. Those assumptions conflict with current research on the non-linear behavior of biological tissues (29–31) and with findings demonstrating the constancy of joint-space width under load in humans (32).

In contrast, biotensegral organization describes the body as a hierarchically nested network of tension and compression elements. Forces are not transmitted through rigid levers but through dynamic tension patterns that allow self-organization and impact stability, even under changing loads or during unexpected perturbations.

Trajectories of PSF^+^ and PSF^−^ can be interpreted coherently within a biotensegral framework. Attempts to account for these patterns within a strictly lever-based model have led to conceptual inconsistencies that warrant separate, more detailed examination beyond the scope of the present paper and will be addressed separately in future publications.

As a theoretical analogy rather than an empirical demonstration, this shift in perspective—from deficit-oriented interpretation toward systemic capacities of the equine body—can be framed within the concept of self-organization. In this context, self-organization refers to the capacity of living systems to functionally integrate externally acting forces within their internal structural organization. This interpretation is conceptually consistent with multi-scale biological findings: at the cellular level (33), in embryonic morphogenesis (34), within the fascial network (35, 36), and in whole-organism biotensegrity models (9, 37).

### 3.2 The pull – differentiation between horse-initiated horizontal forward tension toward the bit in contrast to rider-initiated force on the bit

Across the documented cases, the restoration of coordinated movement patterns was consistently linked to the establishment of horizontal tension toward the bit. Although this mechanical contact might appear to represent an external influence, its effect arises primarily from the horse's active engagement rather than passive response.

In this context, the bit can be understood not as an external tool acting upon the horse, but as a functional interface through which the animal organizes its relationship with external forces as well as with its rider. When this tension is maintained at an appropriate intensity, it provides a consistent directional reference that enables the system to distribute load symmetrically and stabilize posture without excessive muscular effort. The transition from PSF^−^ to PSF^+^ thus appears to depend not only on local adaptation within the limbs or spine, but on the re-emergence of systemic coherence, potentially facilitated by controlled interaction with mechanical boundaries.

The mechanism described also explains why effective rehabilitation and subsequent performance improvement can occur even when the rider's technical skills are limited. The horse-initiated horizontal tension toward the bit provides structural guidance that supports self-organization at the systemic level, allowing the horse to modulate force transmission efficiently and develop sustainable locomotor patterns. In this sense, the bit functions less as a tool of control and more as a structural interface that couples the horse and rider into a shared biotensegral system.

It takes two to pull. Rein tension is inherently relational and does not, in itself, reveal whether force is initiated by the horse or imposed by the rider. From an equine welfare perspective, the decisive question is whether the rein connection emerges from the horse's active engagement and remains functionally integrated, or whether rider-induced pressure disrupts structural and functional organization. Distinguishing between these conditions therefore requires interpretation based on observable postural and movement markers (see section 2.6), rather than relying solely on isolated force measurements.

Several observable criteria allow this distinction to be evaluated:

In animal- and specifically horse welfare research, a range of body language markers and behavioral modifications indicating pain and stress (38–40) are known. Furthermore, measurable screening targets to assess pain in horses, such as for example the Horse Grimace Scale (41) or the Ridden Horse Pain Ethogram (42) provide tools for objective assessment. An investigation of such behavioral- and body language expressions of the horse can help to examine whether the current interaction is experienced as comfortable or stressful by the horse. The absence of stress and pain markers may be interpreted as a minimal welfare criterion, whereas signs of positive engagement require contextual interpretation beyond the mere absence of stress markers. Future research may investigate whether anticipatory behaviors and voluntary engagement during saddling and mounting could serve as indicators of positive valence in ridden contexts.

The PSF dynamic functional markers listed in Table 3 provide measurable reference points for indirectly evaluating the nature of rein interaction. Rider-induced pressure aimed at shaping posture or forcing submission would be expected to compromise PSF^+^ characteristic markers immediately, whereas horse-initiated engagement is associated with their preservation.

Furthermore, the proposed distinction between horse-initiated engagement and rider-induced pressure can also be examined using objective biomechanical measurement techniques, such as saddle pressure analysis. Previous saddle pressure studies have shown that, in sound horses ridden by competent riders, pressure profiles tend to be more evenly distributed across the contact surface (43). Lameness and saddle slip—which may themselves be interrelated—have been reported to alter these pressure distributions (44). Increased saddle pressure has been associated with reduced spinal range of motion and diminished hindlimb protraction (45). Localized peak pressures have been linked to back discomfort (46) and a more caudal displacement of the center of pressure has been reported to increase discomfort in the horse (47).

Within the present framework, the assumed difference in structural organization between horse-initiated tension toward the bit and rider-induced rein traction is expected to generate distinct pressure patterns at the saddle interface. Under controlled comparative conditions using saddle pressure measurement systems, active, self-generated forward engagement by the horse toward the bit is hypothesized to produce a relatively uniform pressure profile across the stride cycle, characterized by lower amplitude variation and reduced peak formation. In contrast, externally imposed rein traction is expected to increase peak pressures, pressure amplitude, and tangential saddle motion, resulting in a less stable pressure distribution.

### 3.3 FIT as practical implementation of PSF

We understand Functional and Interactive Training (FIT), a retrospectively introduced label used to describe the training principles applied in the documented cases, as the practical implementation of this systemic concept rather than being a proprietary method. FIT is based on coordinated, efficient self-organized motion and on the continuous interaction between the body and external forces rather than on isolated therapeutic correction. The approach integrates the key parameters addressed in this study: the regulatory role of the LSJ, phase shifts during the stance phase, the influence of hoof geometry, and the horse-initiated horizontal tension toward the bit as a tensegral connection between horse and rider. Although the observable outcomes may vary depending on the developmental stage of horse and rider and the situational demands, the process was consistently observed to advance toward greater postural stability, load balance, and systemic coherence.

In this context, FIT performs as the operational bridge between theoretical understanding and functional reversal, translating the mechanisms of self-organization into practical outcomes that can be observed and replicated across diverse cases (see case studies in the Supplementary material).

These findings indicate that, once veterinary clearance for training has been obtained, training based on systemic interaction rather than mechanical correction can achieve measurable functional improvements within a short period. As soon as the horse exercises in constructive coordination with external forces, impact stability, movement efficiency, and motor competence visibly increase, while many clinical abnormalities lose significance.

In contrast, owner reports support the view that training and rehabilitation approaches focused primarily on correcting posture, strengthening isolated muscle groups, or shaping specific movement patterns did not consistently yield sustainable improvements, which aligns with existing theoretical and empirical frameworks in human sports science (48).

### 3.4 Contextualization of the PSF framework

**Figure 11:**
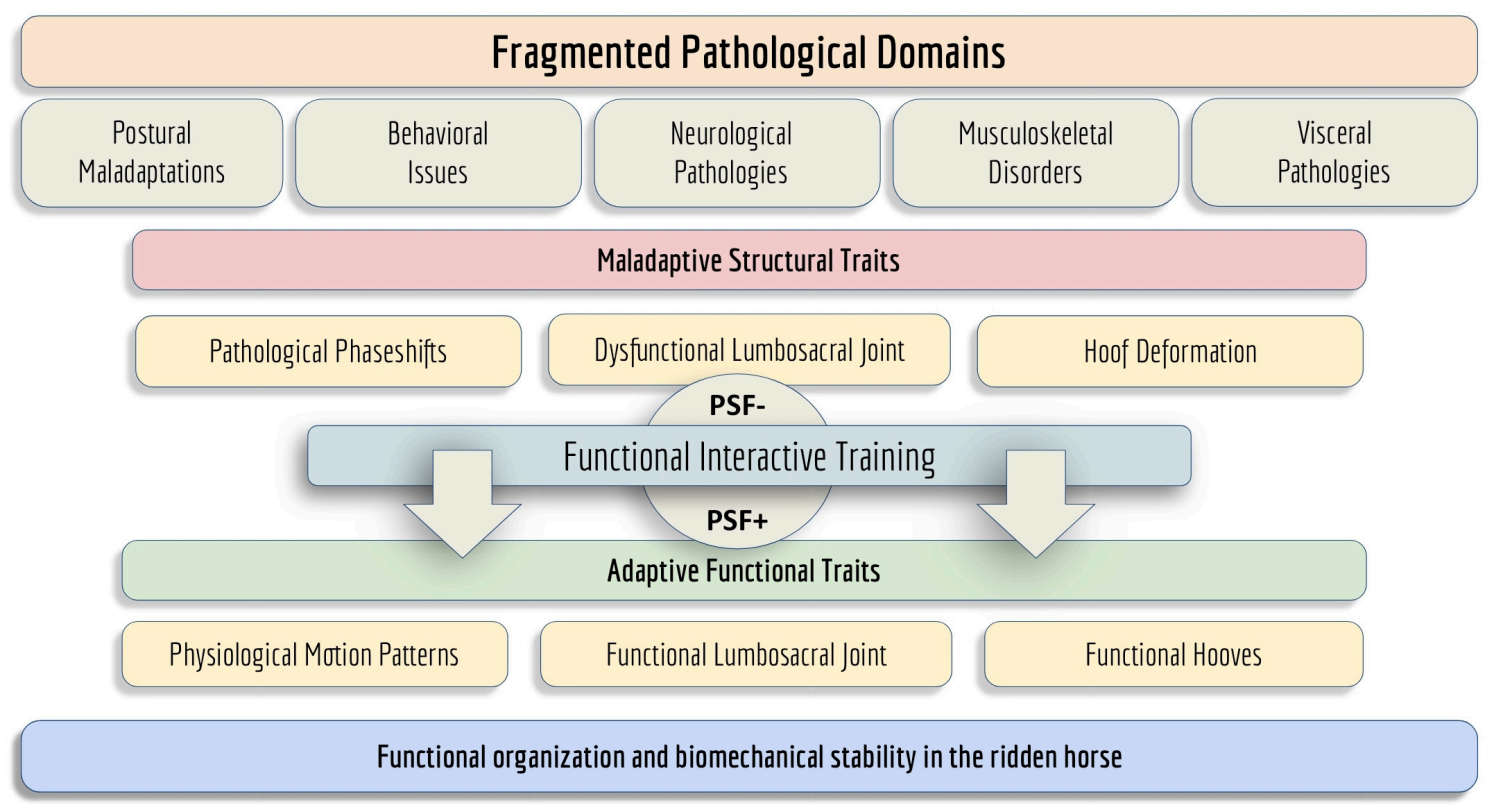
Schematic representation of the PSF framework. The upper section illustrates the fragmented pathological domains and maladaptive structural traits commonly observed in horses affected by PSF^−^. The central transition zone represents the dynamic shift from PSF^−^ to PSF^+^ (functional inversion and -reversion). The lower section visualizes the adaptive functional traits and coordinated motion patterns associated with PSF^+^, culminating in the restoration of functional organization and biomechanical stability in the ridden horse.

PSF provides a systemic framework linking dispersed findings and diffuse pathologies, ranging from back pain and declining performance to lameness without clear clinical cause, and translates them into coherent preventive and therapeutic concepts (Figure 11). Furthermore, it opens new avenues for interdisciplinary collaboration among veterinary science, movement research, and practical training. Treatment of individual symptoms and pathologies might be required, before functional reversion can enable PSF^+^. The PSF framework does not replace classical veterinary diagnostics and treatment; it rather integrates and complements them.

Various studies have reported associations between conformation traits and lameness as reviewed by Dyson et al. (20). Similarly, the correlation between conformation and health status (49) and performance (50, 51) have been studied. Furthermore, a broad body of literature exists on the relationship between back pain and muscular atrophy, as reviewed by Clayton et al. (52), or lameness and muscular atrophy as recently published by Sullivan et al. (53). The relationship between hoof conformation and the risk of lameness has been discussed earlier in this manuscript (22, 25, 26, 54, 55). In general, the literature reviewed here focuses on isolated structures or single parameters, and demonstrates local associations. Within the PSF framework, however, these findings can be interpreted as components of a broader systemic process. Morphological traits should not be understood as static characteristics but as expressions of dynamic postural and functional patterns (28) that may, over time, converge into a progressive loss of structural integrity and functional organization. We agree with Osborn et al. (56) who suggest the reversibility of local lesions when underlying systemic issues are resolved.

The existing biomechanical literature is extensive yet appears fragmented, involving inconsistent terminology and methodological heterogeneity. Egenvall et al. (57) pointed out that the result of scoping reviews strongly depends on the applied search strategy and that including generally accepted definitions alongside a precise, yet not overly narrow description of the topic may lead to more comprehensive results. Accordingly, our hypotheses and theory seek to promote a consistent vocabulary for describing functional (patho-)physiology in horses.

To our knowledge, no comprehensive studies currently exist yet on the functional relevance and systemic interplay of the conformation traits described in the results section 2.4.1 and in the Supplementary material (search strategies see Supplementary material). The hypotheses presented herein are derived from field observations and retrospectively documented case studies. While statistical significance cannot be inferred due to sample size, the consistency of similar findings across different contexts suggests the existence of an underlying systemic pattern.

Empirical validation of the PSF framework therefore requires systematic experimental investigations using objective measurement techniques capable of capturing movement patterns, load distributions and temporal coordination under standardized conditions. The following section outlines potential directions for quantitative validation and hypothesis testing based on measurable biomechanical parameters.

### 3.5 Future research questions and measurement protocols

#### 3.5.1 Existing measurement modalities in equine locomotion research

A broad range of objective measurement techniques is currently available in equine locomotion research (58–60). These methods have been used to quantify kinematic, kinetic, temporal, and thermographic parameters under controlled conditions and provide a methodological foundation for future hypothesis-driven investigations.

Sensor-based systems, particularly inertial measurement units (IMUs), are widely applied to assess upper body movement symmetry, stride timing, and asymmetry thresholds relevant to lameness detection (61, 62). Optical motion capture systems have been used to record detailed kinematic data of limbs, trunk, head, neck, and pelvis (63–66). Force plate measurements provide high-resolution data on vertical GRF curve morphology, peak magnitude, peak timing, and temporal distribution across the stance phase. These systems also permit evaluation of limb symmetry and load distribution patterns. Combined motion capture and force plate setups enable synchronized assessment of kinematics and GRF, allowing detailed analysis of stance phase timing, peak force characteristics, and interlimb coordination (21).

Several studies have demonstrated that biomechanical parameters may vary between horses and across measurement sessions, emphasizing the importance of repeated measurements and controlled conditions (61, 62, 65). Hardeman et al. reported that between-measurement variability in range-of-motion parameters may exceed differences observed between symptomatic and asymptomatic horses or before and after intervention (64), highlighting the need for careful interpretation of longitudinal data. Pfau et al. further demonstrated that upper body movement symmetry can vary depending on surface conditions, straight-line vs. circular movement, and direction (62).

Infrared thermography (IRT) has been reviewed as a non-invasive method to detect sick animals (67) and could be employed in our context for detecting regional thermal asymmetries associated with altered load distribution or pathology. Standardized thermographic assessment protocols allow quantification of interlimb temperature differences and localized hotspots under controlled environmental conditions.

Saddle pressure measurement systems have been employed to evaluate pressure distribution patterns during ridden exercise, including associations with lameness, saddle slip, and altered spinal range of motion (43–47, 68). These technologies provide quantitative insight into dynamic load transfer between horse and rider.

Collectively, these established measurement modalities offer objective tools for analyzing locomotor coordination, load distribution, asymmetry, and variability. Their methodological robustness and prior validation in equine research provide a foundation for future investigations addressing systemic movement organization.

#### 3.5.2 Verification of static and dynamic PSF markers

##### 3.5.2.1 LSJ function – tuber coxae trajectory

Research Question 1: Are there measurable differences in kinematic parameters of tubera coxarum motion between PSF^−^ and PSF^+^ states?

Based on the proposed geometric differences between a functionally closed LSJ and a persistently open configuration, the movement trajectories of the tubera coxarum relative to each other are expected to differ.

- In PSF^+^, pelvic motion is hypothesized to be characterized predominantly by vertically oriented oscillation of the tubera coxarum with minimal alternating anteroposterior displacement between left and right sides, reflecting distributed axial rotation along a continuous spinal axis.
- In PSF^−^, a functional discontinuity at the lumbosacral junction is expected to result in increased alternating anteroposterior displacement of the tubera coxarum, indicating localized pelvic rotation rather than distributed axial motion.

These predictions could be tested using motion capture systems or IMUs. Future validation could also employ skin-mounted markers placed over L6 and S1 spinous processes to quantify dynamic interspinous distance changes.

##### 3.5.2.2 GRF profile

Research Question 2: Are there measurable differences in vertical GRF curve morphology, peak timing, and tangential relative motion during stance phase between PSF^−^ and PSF^+^ states?

The vertical GRF curve during the stance phase in trot is expected to differ between PSF^+^ and PSF^−^ states.

- In PSF^+^, a single, clearly defined peak approximately around mid-stance is hypothesized under steady movement conditions.
- In PSF^−^, a flatter curve with either a double peak or a temporally shifted peak may be observed.

Tangential relative motion during stance phase is expected to be lower in PSF^+^ compared to PSF^−^.

High-resolution force plates with synchronized kinematic capture would allow assessment of GRF curve morphology and timing.

##### 3.5.2.3 Limb symmetry

Research Question 3: Are there measurable differences in limb symmetry in vertical GRF between PSF^−^ and PSF^+^ states?

PSF^−^ is hypothesized to show greater asymmetry between left and right forelimbs and/or hindlimbs in force plate recordings. This asymmetry may involve differences in:

- Peak magnitude,
- Peak timing,
- Temporal distribution across the stance phase.

High-resolution force plate measurements with synchronized kinematic capture would allow assessment of GRF curve morphology and timing.

##### 3.5.2.4 Stance-to-suspension ratio

Research Question 4: Are stance-to-suspension ratios, measured via force plates or inertial sensors under standardized speed conditions, different between PSF^−^ and PSF^+^ states?

Using force plates or body-mounted sensors (e.g., IMUs), the temporal relationship between stance and suspension phases can be quantified. PSF^+^ is hypothesized to exhibit a relatively increased suspension phase compared to PSF^−^ under comparable speed conditions.

##### 3.5.2.5 IRT

Research Question 5: Can standardized IRT recordings detect quantifiable changes in regional thermal symmetry and load-related hotspots that differentiate PSF^−^ from PSF^+^ states and track functional reversion over time?

IRT may serve as a complementary, non-invasive method to detect changes in regional load distribution associated with functional reorganization. Standardized longitudinal recordings could quantify interlimb thermal asymmetries as well as regional hotspots and hypothermic areas in the lumbosacral and distal limb regions.

Within the PSF framework, PSF^−^ horses are hypothesized to exhibit greater thermal asymmetry, localized hyperthermia in overloaded structures and relative hypothermia in under-recruited regions compared to PSF^+^ horses. A normalization of these thermal patterns over time may serve as a physiological correlate of improved systemic load integration.

##### 3.5.2.6 Longitudinal perspective

Research Question 6: Can inter-day variability in locomotor parameters function as a longitudinal predictor of maladaptive load organization and future lameness within the PSF framework?

We hypothesize that PSF^+^ horses will demonstrate significantly lower inter-day variance in stride length, step length, and phase timing than PSF^−^ horses, allowing variability metrics themselves to function as discriminatory markers of systemic organization.

The primary value of these investigations lies in examining long-term associations between movement patterns and the later development of lameness. If the predicted relationships are confirmed, the findings could inform preventive training strategies and contribute to earlier detection of maladaptive load organization.

## 4 Conclusion

The PSF framework conceptualizes progressive structural and functional change in horses as a systemic process with two possible trajectories: Progressive Structural and Functional Loss (PSF^−^) and Progressive Structural and Functional Gain (PSF^+^). Central to this model is the mechanism of functional inversion, describing a systemic reversal of physiological force integration and load-transfer organization.

Rather than replacing established diagnoses, PSF integrates dispersed clinical findings into a higher-order systemic classification. By identifying functional control nodes—particularly the LSJ, hoof morphology, and horse-initiated horizontal tension toward the bit—the framework links structural configuration, locomotor coordination, and rehabilitation strategies within a coherent explanatory structure.

Importantly, PSF generates falsifiable predictions. The proposed static and dynamic markers provide low-threshold screening criteria, while biomechanical measurement approaches offer opportunities for quantitative validation. As such, PSF is presented as a testable hypothesis framework rather than a closed theory.

By offering a consistent systemic vocabulary and operationalizable markers, PSF may facilitate interdisciplinary collaboration and support prevention-oriented veterinary practice. Ultimately, the framework aims to contribute to improved structural resilience, movement efficiency, and welfare in the ridden horse.

## Data Availability

The original contributions presented in the study are included in the article/Supplementary material, further inquiries can be directed to the corresponding author/s.

## Appendix: Glossary

***Control Nodes:*** Key factors of modification within the PSF framework. In horses, the primary control nodes are: (1) the functional state of the LSJ, and (2) hoof management. These nodes represent influence points at system level that can shift the trajectory toward PSF^−^ or PSF^+^. The control nodes are not isolated structures but functional regulators of load, tension, and movement organization.

***FIT – Functional and Interactive Training:*** Operational training context within the PSF framework, retrospectively defined to describe the interaction-based approach applied in the documented cases. FIT emphasizes horse-initiated active horizontal tension toward the bit as a proposed organizing stimulus within the biotensegral system. Within this framework, posture, load distribution, proprioceptive input, and external forces (e.g., ground reaction forces and gravity) are understood to interact in a way that may support systemic reorganization and the emergence of impact stability. FIT does not prescribe specific riding styles or exercises but describes general principles applicable across training contexts. It is presented as a hypothesis-generating framework rather than as a validated therapeutic method.

***Functional Inversion:*** Core mechanism driving PSF^−^. Describes the systemic inversion of physiological roles and force directions: structures that should stabilize begin to destabilize, and movements that should unload tissues generate additional load.

***Functional Reversion:*** Describes the shift from PSF^−^ to PSF^+^, counterpart to functional inversion.

***Impact Stability:*** The transient capacity of the neuro-myofascial system to generate momentarily high stability under load, enabling the efficient absorption and redistribution of short, intense force impulses (primarily ground reaction forces).

***PSF – Progressive Structural and Functional Change***: Systemic framework describing how structure and function in the equine body change over time. PSF includes both deteriorating (PSF^−^) and improving (PSF^+^) trajectories. Proposed translations for international consistency: Spanish – Cambio Progresivo Estructural y Funcional, French – Changement Structural et Fonctionnel Progressif, German – Progressiver Struktur- und Funktionswandel.

***PSF***^**−**^**–** ***Progressive Structural and Functional Loss***: Self-amplifying pattern of declining stability, coordination, and tissue function. Arises when external forces are absorbed maladaptively, leading to compensatory tension, structural overload, and reduced movement quality. Proposed translations for international consistency: Spanish – Deterioro Progresivo Estructural y Funcional, French – Déclin Structural et Fonctionnel Progressif, German – Progressiver Struktur- und Funktionsverlust.

***PSF***^**+**^ **–** ***Progressive Structural and Functional Gain***: Self-stabilizing pattern of improving structure, function, and movement organization. Occurs when external forces are integrated efficiently and the body regains adaptive self-organization. Proposed translations for international consistency: Spanish – Mejoría Progresiva Estructural y Funcional, French – Gain Structural et Fonctionnel Progressif, German – Progressiver Struktur- und Funktionsgewinn.

## References

[1] Ehrle A Lilge S Clegg PD Maddox TW. Equine flexor tendon imaging part 1: Recent developments in ultrasonography, with focus on the superficial digital flexor tendon. Vet J. (2021) 278:105764. doi: 10.1016/j.tvjl.2021.105764 34678500

[2] Landman MAAM De Blaauw JA Hofland LJ Van Weeren PR. Field study of the prevalence of lameness in horses with back problems. Vet Rec. (2004) 155:165. doi: 10.1136/vr.155.6.165 15357376

[3] Haussler KK. “Chiropractic Evaluation and Management of Musculoskeletal Disorders,” Diagnosis and Management of Lameness in the Horse. Elsevier (2003). p. 803 doi: 10.1016/B978-0-7216-8342-3.50101-7

[4] Marshall-Gibson ME Durham MG Seabaugh KA Moorman VJ Ferris DJ. Survey of equine veterinarians regarding primary equine back pain in the United States. Front Vet Sci. (2023) 10:1224605. doi: 10.3389/fvets.2023.1224605 37565081 PMC10411723

[5] Back W Clayton HM. eds. Equine locomotion Second edition Edinburgh [Scotland]. New York, NY: Saunders Elsevier (2013).

[6] Clayton HM Hobbs S-J. The role of biomechanical analysis of horse and rider in equitation science. Appl Anim Behav Sci. (2017) 190:123. doi: 10.1016/j.applanim.2017.02.011

[7] Levin SM. “The Icosahedron as a Biological Support System,” in Proceedings of the 34th Annual Conference on Engineering in Medicine and Biology. Houston, Texas (1981). 404

[8] Scarr G Blyum L Levin SM De Solórzano SL. Moving beyond Vesalius: Why anatomy needs a mapping update. Med Hypotheses. (2024) 183:111257. doi: 10.1016/j.mehy.2023.111257

[9] Scarr G Blyum L Levin SM Lowell De Solórzano S. Biotensegrity is the super-stability hypothesis for biology. Biosystems. (2025) 256:10105569. doi: 10.1016/j.biosystems.2025.105569 40854365

[10] Kelso JAS. Dynamic patterns: the self-organization of brain and behavior. 3rd ed Cambridge, Mass: MIT Press. (1999).

[11] Volesky B. Das Topline Syndrom Warum die Rückenschwäche des Pferdes nur ein Symptom ist. Norderstedt: Books on Demand. (2020).

[12] Ursini T. Therapeutic exercise strategies for topline dysfunction in horses. ASEAN J Psychiatry. (2024) 8:1. doi: 10.54615/2231-7805.8.01.001

[13] McKenzie R. Treat your own back. Raumati Beach, NZ: Spinal Publications New Zealand. (1980).

[14] Travell JG Simons DG. Myofascial Pain and Dysfunction: The Trigger Point Manual. Baltimore: Williams & Wilkins. (1983).

[15] Jeffcott LB. Disorders of the thoracolumbar spine of the horse — a survey of 443 cases. Equine Vet J. (1980) 12:4. doi: 10.1111/j.2042-3306.1980.tb03427.x 7439145

[16] Richter T. Illusion Pferdeosteopathie: von ausgerenkten Wirbeln und anderen Märchen. Schondorf: Wu-Wei-Verl. (2011).

[17] Kattwinkel K. Raus aus der Trageschwäche: Der Schlüssel zur Tragkraft Deines Pferdes. Self-published. (2025).

[18] Drack M Pouvreau D. On the history of Ludwig von Bertalanffy's “General Systemology”, and on its relationship to cybernetics – part III: convergences and divergences. Int J Gen Syst. (2015) 44:523. doi: 10.1080/03081079.2014.1000642 26612966 PMC4610108

[19] Eser K Diehl M. Die Bedeutung des Lumbosakralgelenks für das Pferd im Allgemeinen und das Reitpferd im Speziellen. Z Für Ganzheitliche Tiermed. (2017) 31:132. doi: 10.1055/s-0043-113876

[20] Dyson S. Is there an association between conformation and lameness? UK-Vet Equine. (2018) 2:57. doi: 10.12968/ukve.2018.2.2.57

[21] Hobbs SJ Bertram JEA Clayton HM. An exploration of the influence of diagonal dissociation and moderate changes in speed on locomotor parameters in trotting horses. PeerJ. (2016) 4:e2190. doi: 10.7717/peerj.2190 27413640 PMC4933092

[22] Dyson SJ Tranquille CA Collins SN Parkin TDH Murray RC. An investigation of the relationships between angles and shapes of the hoof capsule and the distal phalanx: Angles and shapes of hoof and distal phalanx. Equine Vet J. (2011) 43:295. doi: 10.1111/j.2042-3306.2010.00162.x 21492206

[23] Kalka K Pollard D Dyson SJ. An investigation of the shape of the hoof capsule in hindlimbs, its relationship with the orientation of the distal phalanx and comparison with forelimb hoof capsule conformation. Equine Vet Educ. (2021) 33:422. doi: 10.1111/eve.13341

[24] Antonioli ML Canola PA De Carvalho JRG Fonseca MG Ferraz GDC. Immediate Effect of Hoof Trimming on Hoof and Thoracic Joint Angles in Mangalarga Mares. Animals. (2023) 13:2490. doi: 10.3390/ani13152490 37570298 PMC10416872

[25] Mata F Franca I Araújo J Paixão G Lesniak K Cerqueira JL. Investigating Associations between Horse Hoof Conformation and Presence of Lameness. Animals. (2024) 14:2697. doi: 10.3390/ani14182697 39335286 PMC11444133

[26] Pezzanite L Bass L Kawcak C Goodrich L Moorman V. The relationship between sagittal hoof conformation and hindlimb lameness in the horse. Equine Vet J. (2019) 51:464. doi: 10.1111/evj.13050 30472759

[27] Elbrønd VS Schultz RM. Deep Myofascial Kinetic Lines in Horses, Comparative Dissection Studies Derived from Humans. Open J Vet Med. (2021) 11:14. doi: 10.4236/ojvm.2021.111002

[28] Bürger U Zietzschmann O. Der Reiter formt das Pferd: Tätigkeit und Entwicklung der Muskeln des Reitpferdes. FNverlag. (2016).

[29] Todros S Biz C Ruggieri P Pavan PG. Experimental Analysis of Plantar Fascia Mechanical Properties in Subjects with Foot Pathologies. Appl Sci. (2021) 11:1517. doi: 10.3390/app11041517

[30] Bonaldi L Berardo A Pirri C Stecco C Carniel EL Fontanella CG. Mechanical Characterization of Human Fascia Lata: Uniaxial Tensile Tests from Fresh-Frozen Cadaver Samples and Constitutive Modelling. Bioengineering. (2023) 10:226. doi: 10.3390/bioengineering10020226 36829719 PMC9952725

[31] Creze M Lagache A Duparc F Broqué M Persohn S Slama C . Ex vivo mechanical properties of human thoracolumbar fascia and erector spinae aponeurosis under traction loading and shear wave elastography. J Mech Behav Biomed Mater. (2025) 168:107028. doi: 10.1016/j.jmbbm.2025.107028 40262430

[32] Hakkak F Jabalameli M Rostami M Parnianpour M. The Tibiofemoral Joint Gaps -An Arthroscopic Study. SDRP J Biomed Eng. (2017) 1:1. doi: 10.25177/JBE.1.1.1

[33] Wedlich-Söldner R Betz T. Self-organization: the fundament of cell biology. Philos Trans R Soc B Biol Sci. (2018) 373:20170103. doi: 10.1098/rstb.2017.0103 29632257 PMC5904291

[34] Newman SA. ‘Biogeneric' developmental processes: drivers of major transitions in animal evolution. Philos Trans R Soc B Biol Sci. (2016) 371:1701. doi: 10.1098/rstb.2015.0443 27431521 PMC4958937

[35] Armstrong C. The architecture and spatial organization of the living human body as revealed by intratissular endoscopy – an osteopathic perspective. J Bodyw Mov Ther. (2020) 24:138. doi: 10.1016/j.jbmt.2019.11.005 31987534

[36] Guimberteau JC Sawaya ET Armstrong C. New perspectives on the organization of living tissue and the ongoing connective tissue/fascia nomenclature debate, as revealed by intra-tissue endoscopy that provides real-time images during surgical procedures. Life. (2025) 15:791. doi: 10.3390/life15050791 40430217 PMC12112776

[37] Levin SM Lowell De Solórzano S. Bouncing bones—ancient wisdom meets modern science in a new take on locomotion. Front Physiol. (2024) 15:1432410. doi: 10.3389/fphys.2024.1432410 39403564 PMC11471642

[38] Mota-Rojas D Whittaker AL Lanzoni L Bienboire-Frosini C Domínguez-Oliva A Chay-Canul A . Clinical interpretation of body language and behavioral modifications to recognize pain in domestic mammals. Front Vet Sci. (2025) 12:1679966. doi: 10.3389/fvets.2025.1679966 41169680 PMC12570343

[39] Hernández-Avalos I Mota-Rojas D Mendoza-Flores JE Casas-Alvarado A Flores-Padilla K Miranda-Cortes AE . Nociceptive pain and anxiety in equines: Physiological and behavioral alterations. Vet World. (2021) 14:2984. doi: 10.14202/vetworld.2021.2984-2995 35017848 PMC8743789

[40] Zimmermann B Castro ANC Lendez PA Carrica Illia M Carrica Illia MP Teyseyre AR . Anatomical and functional basis of facial expressions and their relationship with emotions in horses. Res Vet Sci. (2024) 180:105418. doi: 10.1016/j.rvsc.2024.105418 39303445

[41] Dalla Costa E Minero M Lebelt D Stucke D Canali E Leach MC. Development of the Horse Grimace Scale (HGS) as a Pain Assessment Tool in Horses Undergoing Routine Castration. PLoS ONE. (2014) 9:e92281. doi: 10.1371/journal.pone.0092281 24647606 PMC3960217

[42] Dyson S. The Ridden Horse Pain Ethogram. Equine Vet Educ. (2022) 34:372. doi: 10.1111/eve.13468

[43] Fruehwirth B Peham C Scheidl M Schobesberger H. Evaluation of pressure distribution under an English saddle at walk, trot and canter. Equine Vet J. (2004) 36:754. doi: 10.2746/0425164044848235 15656510

[44] Greve L Dyson SJ. An investigation of the relationship between hindlimb lameness and saddle slip. Equine Vet J. (2013) 45:570. doi: 10.1111/evj.12029 23360352

[45] Martin P Chateau H Pourcelot P Duray L Chèze L. Effects of a prototype saddle (short panels) on the biomechanics of the equine back: preliminary results. Comput Methods Biomech Biomed Engin. (2015) 18:1990. doi: 10.1080/10255842.2015.1069591 26230480

[46] Werner D Nyikos S Kalpen A Geuder M Haas C Vontobel H-D . Von Rechenberg B. Pressure measurements under the saddle: a study using an electronic saddle mat system (Novel GmbH): Pferdeheilkunde. Equine Med. (2002) 18:125. doi: 10.21836/PEM20020201

[47] Roost L Ellis AD Morris C Bondi A Gandy EA Harris P . The effects of rider size and saddle fit for horse and rider on forces and pressure distribution under saddles: A pilot study. Equine Vet Educ. (2020) 32:151. doi: 10.1111/eve.13102

[48] Woods CT McKeown I Rothwell M Araújo D Robertson S Davids K. Sport Practitioners as Sport Ecology Designers: How Ecological Dynamics Has Progressively Changed Perceptions of Skill “Acquisition” in the Sporting Habitat. Front Psychol. (2020) 11:654. doi: 10.3389/fpsyg.2020.00654 32390904 PMC7194200

[49] Jönsson L Näsholm A Roepstorff L Egenvall A Dalin G Philipsson J. Conformation traits and their genetic and phenotypic associations with health status in young Swedish warmblood riding horses. Livest Sci. (2014) 163:12. doi: 10.1016/j.livsci.2014.02.010 24871069

[50] Suprun I Turchenko V. Sports achievements and morphometric characteristics of Oldenburg horse lines. Anim Sci Food Technol. (2024) 15:87. doi: 10.31548/animal.3.2024.87

[51] Jönsson L Egenvall A Roepstorff L Näsholm A Dalin G Philipsson J. Associations of health status and conformation with longevity and lifetime competition performance in young Swedish Warmblood riding horses: 8,238 cases (1983–2005). J Am Vet Med Assoc. (2014) 244:1449. doi: 10.2460/javma.244.12.1449 24871069

[52] Clayton HM. Equine back pain reviewed from a motor control perspective. Comp Exerc Physiol. (2012) 8:145. doi: 10.3920/CEP12023

[53] Sullivan HM Acutt EV Barrett MF Salman MD Ellis KL King MR. Influence of Chronic Lameness on Thoracolumbar Musculus Multifidus Structure in the Horse. J Equine Vet Sci. (2022) 117:104053. doi: 10.1016/j.jevs.2022.104053 35753637

[54] Dyson SJ Tranquille CA Collins SN Parkin TDH Murray RC. External characteristics of the lateral aspect of the hoof differ between non-lame and lame horses. Vet J. (2011) 190:364. doi: 10.1016/j.tvjl.2010.11.015 21169041

[55] Holroyd K Dixon JJ Mair T Bolas N Bolt DM David F . Variation in foot conformation in lame horses with different foot lesions. Vet J. (2013) 195:361. doi: 10.1016/j.tvjl.2012.07.012 22981735

[56] Osborn ML Cornille JL Blas-Machado U Uhl EW. The equine navicular apparatus as a premier enthesis organ: Functional implications. Vet Surg. (2021) 50:713. doi: 10.1111/vsu.13620 33710628 PMC8251969

[57] Egenvall A Byström A Lindsten A Clayton HM A. Scoping Review of Equine Biomechanics Revisited. J Equine Vet Sci. (2022) 113:103920. doi: 10.1016/j.jevs.2022.103920 35257826

[58] Serra Bragança FM Rhodin M Van Weeren PR. On the brink of daily clinical application of objective gait analysis: What evidence do we have so far from studies using an induced lameness model? Vet J. (2018) 234:11. doi: 10.1016/j.tvjl.2018.01.006 29680381

[59] Egan S Brama P McGrath D. Research trends in equine movement analysis, future opportunities and potential barriers in the digital age: A scoping review from 1978 to 2018. Equine Vet J. (2019) 51:813. doi: 10.1111/evj.13076 30659639

[60] Crecan CM Pesştean CP. Inertial Sensor Technologies—Their Role in Equine Gait Analysis, a Review. Sensors. (2023) 23:6301. doi: 10.3390/s23146301 37514599 PMC10386433

[61] Macaire C Hanne-Poujade S De Azevedo E Denoix JM Coudry V Jacquet S . Investigation of Thresholds for Asymmetry Indices to Represent the Visual Assessment of Single Limb Lameness by Expert Veterinarians on Horses Trotting in a Straight Line. Animals. (2022) 12:3498. doi: 10.3390/ani12243498 36552418 PMC9774792

[62] Pfau T Bolt DM Fiske-Jackson A Gerdes C Hoenecke K Lynch L . Linear Discriminant Analysis for Investigating Differences in Upper Body Movement Symmetry in Horses before/after Diagnostic Analgesia in Relation to Expert Judgement. Animals. (2022) 12:762. doi: 10.3390/ani12060762 35327159 PMC8944550

[63] Roepstorff C Dittmann MT Arpagaus S Serra Bragança FM Hardeman A Persson-Sjödin E . Reliable and clinically applicable gait event classification using upper body motion in walking and trotting horses. J Biomech. (2021) 114:110146. doi: 10.1016/j.jbiomech.2020.110146 33290946

[64] Hardeman AM Byström A Roepstorff L Swagemakers JH Van Weeren PR Serra Bragança FM. Range of motion and between-measurement variation of spinal kinematics in sound horses at trot on the straight line and on the lunge. PLoS ONE. (2020) 15:e0222822. doi: 10.1371/journal.pone.0222822 32097432 PMC7041811

[65] Byström A Hardeman AM Serra Bragança FM Roepstorff L Swagemakers JH van Weeren PR . Differences in equine spinal kinematics between straight line and circle in trot. Sci Rep. (2021) 11:12832. doi: 10.1038/s41598-021-92272-2 34145339 PMC8213771

[66] Egenvall A Engström H Byström A. Back motion in unridden horses in walk, trot and canter on a circle. Vet Res Commun. (2023) 47:1831. doi: 10.1007/s11259-023-10132-y 37127806 PMC10698108

[67] Mota-Rojas D Martínez-Burnes J Casas-Alvarado A Gómez-Prado J Hernández-Ávalos I Domínguez-Oliva A . Clinical usefulness of infrared thermography to detect sick animals: frequent and current cases. CABI Rev. (2022) cabireviews:202217040. doi: 10.1079/cabireviews202217040

[68] Greve L Dyson S. The horse–saddle–rider interaction. Vet J. (2013) 195:275. doi: 10.1016/j.tvjl.2012.10.020 23177524

[69] Diehl M. Der Konsens. Die Pferde Sind Nicht Das Problem (2021) https://www.die-pferde-sind-nicht-das-problem.de/der-konsens (Accessed December 14, 2025).

[70] Erklärvideos LSG (The Key Role of the Lumbosacral Joint). (2021). https://www.die-pferde-sind-nicht-das-problem.de/shop/Erklarvideos-LSG-c120109503 (Accessed December 14, 2025).

[71] Die Hebel im Pferd - Teil I - Die Hinterhand. (2021). https://www.youtube.com/watch?v=9qzJKUERNTw (Accessed December 14, 2025).

[72] Die Hebel im Pferd - Teil II - Die Quadratur des Kreises. (2021). https://www.youtube.com/watch?v=PMtwNkEEvvA (Accessed December 14, 2025).

[73] Die Hebel im Pferd - Teil III - Angewandte Hebel. (2021). https://www.youtube.com/watch?v=evC3xGaPpgw (Accessed December 14, 2025).

[74] Die Hebel im Pferd - Teil IV - Die LSG-These. (2021). https://www.youtube.com/watch?v=HSpNgrnq0Mw (Accessed December 14, 2025).

[75] Die Hebel im Pferd - Teil V - Die Vorhand. (2021). https://www.youtube.com/watch?v=Y0H6DbxQ_tM (Accessed December 14, 2025).

[76] Diehl M. Knäckebrot fürs Hirn. Die Pferde Sind Nicht Das Problem (2025) https://www.die-pferde-sind-nicht-das-problem.de/knaeckebrot-fuer-s-hirn (Accessed December 14, 2025).

[77] Diehl M. Hardtack for your brain. Die Pferde Sind Nicht Das Problem (2025) https://www.die-pferde-sind-nicht-das-problem.de/hardtack-for-your-brain (Accessed December 14, 2025).

