# Supplementary material for: Progressive structural and functional change in horses: a conceptual framework for systemic equine (patho-)physiology
Source: Front Vet Sci. 2026 Apr 7;13:1767386. doi: 10.3389/fvets.2026.1767386 (PMC13095508; doi:10.3389/fvets.2026.1767386)
Supplement: Supplementary file 1 [file Data_Sheet_1.pdf]

PSF Supplementary Information

Contents

Contents ... 1
1 Literature Search Methodology ... 2
2 Secondary Pathologies, Anomalies and Owner's Efforts accompanying PSF ... 3
2.1 Clinical Findings ... 3
2.2 Conformational and Posture Anomalies ... 3
2.3 Functional Anomalies ... 4
2.4 Behavioral Anomalies with Potential Link to Clinical Issues ... 4
2.5 Examples of Owner Efforts accompanying PSF^{-} ... 4
3 PSF Case Studies ... 5
3.1 Case Study 1: Functional Recovery in a Pony with Chronic Headshaking and Cervical Pain ... 7
3.2 Case Study 2: Progressive Structural and Functional Change in a Horse with Severe ECVM ... 14
3.3 Case Study 3: Progressive Structural and Functional Change in a Warmblood Gelding with Recurrent Suspensory Ligament Injuries and Gastric Ulcers ... 21
3.4 Case Study 4: Progressive Structural and Functional Gain (PSF^{+}) in a Former Racehorse with Chronic Pain and Headshaking ... 28
3.5 Case Study 5: Systemic Recovery Limited by Distal Hoof Asymmetry during a PSF^{+} Transition in a German Riding Pony ... 34
3.6 Case Study 6: Systemic and Functional Rehabilitation in a Horse with Cervical Pathologies and REM Sleep Disorder ... 41
3.7 Case Study 7: Physiological Hoof Management and Changes in Training Methods as a Pathway to Sustainable Recovery from Chronic Lameness ... 53
3.8 Case Study 8: PSF^{+} Development and its Limitations in a High-Level Endurance Horse ... 58
3.9 Case Study 9: Reversal of a Croup-High Posture within a PSF^{+} Framework under low Training Frequency and Intensity ... 65
3.10 Case Study 10: A Reference Case of Progressive Structural and Functional Gain (PSF^{+}) in a High-Mileage Trail and TREC Horse ... 69
4 Author Biographies ... 75

1 Literature Search Methodology

A structured literature search was conducted using PubMed and Google Scholar; additional searches and full-text access were complemented via ResearchGate. The search terms were combined using Boolean operators (AND/OR) as supported by each platform. A range of search terms and combinations of them was used, such as for example:

- systemic orthopedic disease in equines
- equine musculoskeletal diseases
- equine topline syndrome
- equine myofascial dysfunction
- pathogenesis diffuse lameness in horses
- poor performance in horses
- conformation and lameness in horses
- conformation and pathologies in horses

None of these searches revealed the use of an umbrella term comparable to PSF in titles, abstracts, or keywords. No studies addressing an underlying systemic process to secondary pathologies in scattered diagnostic domains or patho-physiologies in general were found.

2 Secondary Pathologies, Anomalies and Owner's Efforts accompanying PSF

Horses exhibiting PSF typically present with a broad range of secondary pathologies and abnormalities arising as downstream manifestations of the systemic processes described in the main manuscript. These findings frequently span several domains—musculoskeletal, neurological, visceral, postural, functional, and behavioral—and tend to co-occur rather than appear in isolation.

Owners commonly respond to these multifaceted problems with considerable effort, seeking solutions through diverse management, training, and therapeutic strategies. These efforts were self-initiated and varied widely among owners. While many of these measures provide temporary relief, they seldom lead to sustained improvement when the underlying systemic process remains unaddressed.

The following overview summarizes the secondary pathologies and abnormalities most frequently associated with PSF, together with the range of owner responses typically observed in practice.

2.1 Clinical Findings

- Musculoskeletal:
  - Osteoarthritis
  - Sacroiliac pain / sacroiliac disease
  - Kissing spines
  - Tendinopathies / desmopathies
  - Podotrochleosis / navicular syndrome
  - Regional muscular hypertonicity
- Congenital:
  - ECVM (Equine Complex Vertebral Malformation)
- Visceral:
  - Respiratory disorders
  - Susceptibility to colic
- Neurological / gait-related:
  - Stumbling
  - Marked asymmetry of gaits, increasing under load
  - Unclear lameness without musculoskeletal findings
- Other:
  - REM sleep deficiency

2.2 Conformational and Posture Anomalies

- Caudally displaced forelimbs
- Cranially displaced hindlimbs
- Steeper fore hooves than hind hooves (>48°, <50°)
- High heels at fore hooves and low, underrun heels at hind hooves
- Lordosis of the thoracic spine
- Dropped/lowered thorax
- Pendulous abdomen ("hay belly")

- Heave line
- Pointed croup (“hunters bump”)
- Steeply tilted pelvis
- Pelvic obliquity or pelvic asymmetry
- Atrophied epaxial musculature
- Difficult saddle fit (“impossible to saddle”)

2.3 Functional Anomalies

- Irregular rhythm / rider-induced gait asymmetry (“bridle lameness”)
- Phase shifts (prolonged second half of stance phase of the forelimbs)
- Hasty or hurried gait
- Forging, brushing or overreaching
- Dragging gaits / toe dragging
- Hooves pivoting or rotating on the ground

2.4 Behavioral Anomalies with Potential Link to Clinical Issues

- Spooking
- Bolting
- Hesitant, resisting, or refusing
- Restless or nervous chewing on the bit
- Lack of interest or disengagement

2.5 Examples of Owner Efforts accompanying PSF^{-}

- Comprehensive veterinary care
- Regular physiotherapeutic, osteopathic and/or chiropractic treatments
- Acupuncture
- Self-directed therapeutic education
- Changes in riding styles and tack
- Professional training
- Rehabilitation stays
- Optimized husbandry and management
- Dietary supplements

3 PSF Case Studies

The supplementary case studies illustrate the emergence and reversible nature of PSF and the transition from PSF^{-} toward PSF^{+}. They provide structured observational material that contextualizes the PSF framework under real-world training conditions. Their purpose is to illustrate how horses with different backgrounds, levels of prior training, and initial challenges respond when training aims to restore functional self-organization under load.

Supplementary Table 1 summarizes the sample characteristics of the horses represented in the case studies. Note that this table represents an initial screening of the case material and highlights what we considered most salient. It is not a complete dataset and does not constitute a statistical analysis. These case reports are descriptive and narrative in nature and not intended as clinical trials. They are not presented as evidence. Instead, they document consistent, independently reported patterns of change in posture, movement organization, and behavior that emerged when owners applied the training principles of FIT as described in the main manuscript. Across cases, we noted recurring tendencies toward more consistent rhythm, steadier posture, more coordinated forward movement, and a decline in behaviors that are often interpreted as signs of discomfort.

The rationale for including these case studies is twofold. First, they offer transparency regarding the practical observations that informed the conceptual development of the PSF framework. Second, they provide a preliminary foundation for future hypothesis-driven research by identifying observable variables, response patterns, and training conditions that appear relevant to equine welfare.

Please note that we are no veterinarians and the presented clinical data are reported from the owner's perspective. Where available, reports of veterinarians and clinics or imaging diagnostics including findings were used as base. Available Data is marked in the clinical data section by an asterisk * following the letter-numbered heading. Where no asterisk is included, data is based on owner reports. The clinical data was included to underline the broad variability of clinical findings associated with PSF^{-} and to demonstrate that even horses with severe and numerous clinical findings can manage a functional reversion and enter PSF^{+}.

Supplementary Material

Supplementary Table 1. *Data base for Table 1 in the main text: Sample characteristics of the retrospectively documented case study horses. a: Age refers to the horse´s age at the end of the documented period (2025). b: Discipline background corresponds to primary training discipline prior to implementation of the PSF⁺/FIT framework. c: "before" denotes status before initiation of FIT, "after" denotes status at end of documentation. d: Rideability status was based on owner report and ability to perform regular training sessions without clinical restriction. e: Secondary pathologies grouped into categories "P"- postural maladaptations, B: behavioral issues, N: neurological pathologies, M: musculoskeletal disorders, V: visceral pathologies.*

Case Number	Age[a]	Birth Year	Sex	Breed	Discipline Background[b]	Geographic Context	Start of Documentation	End of Documentation	Rideability Status Before[c,d]	Rideability Status After[c,d]	Presence of Secondary Pathologies Before[c,e]	Presence of Secondary Pathologies After[c,e]
**1**	11	2014	Gelding	German Riding Pony	English Leisure	Germany	2024	2025	no	full	yes (P, B, N, M)	no
**2**	7	2018	Gelding	Warmblood	English Leisure	Germany	2021	2025	partially	full	yes (P, B, N, M, V)	yes (M)
**3**	13	2012	Gelding	Warmblood	English Competitive (Dressage & Jumping)	Germany	2019	2025	partially	full	yes (P, B, M, V)	no
**4**	15	2010	Gelding	Thoroughbred	Steeplechase	Germany	2021	2025	no	full	yes (P, B, M)	no
**5**	12	2013	Mare	German Riding Pony	English Leisure	Germany	2021	2025	full	full	yes (B, M)	no
**6**	17	2008	Gelding	Warmblood	English Leisure	Germany	2020	2025	no	full	yes (P, B, N, M, V)	no
**7**	12	2013	Gelding	Warmblood	English Competitive (Military)	Switzerland	2020	2025	no	full	yes (P, B, M, V)	no
**8**	16	2009	Gelding	Arabian	Endurance	Germany	2020	2025	full	full	yes (P, M, V)	yes (M)
**9**	17	2008	Mare	Quarter Horse	Western Reining	Germany	2021	2025	no	full	yes (P, B)	no
**10**	9	2016	Gelding	Murgese	TREC	Germany	2020	2025	full	full	yes (V)	no

3.1 Case Study 1: Functional Recovery in a Pony with Chronic Headshaking and Cervical Pain

Case 1 represents a large group of horses that show severe behavioral and functional disturbances without distinct clinical findings, often leaving owners without a clear path for intervention.

A German Riding Pony gelding initially presented as an unrideable and idiopathic headshaker, showing pronounced gait irregularities, generalized pain reactions, and behavioral signs of physical discomfort, yet without any obvious clinical cause.

Following a change of ownership, training and hoof care were transitioned to the PSF^{+} approach and barefoot trimming focused on structural balance and self-organization. Within six months, the horse exhibited marked improvement: pain reactions diminished to a minimal level, all movement phases normalized, and the pony became fully rideable again in arena, track, and outdoor work.

The available data for this case originate from owner and trainer documentation since September 2024. Structural and functional changes were monitored through repeated photographic recordings throughout the observation period.

Hoof conformation improved substantially, with the coronary band angle increasing from ~20° on the front hooves and lowering from >30° on the hind hooves to ~30° on all four hooves, indicating restored load distribution and postural stability. This case exemplifies how, even in the absence of specific pathological findings, significant functional and behavioral dysfunctions can be resolved through targeted reorganization of movement and load management based on the PSF^{+} concept.

3.1.1 General information on case study 1 subject

Case Identifier: Case 1
Date of birth: 09.03.2014
Breed: German Riding Pony
Sex: Gelding
Trainer: Saskia Brieger

3.1.2 Case 1 PSF^{-} development

3.1.2.1 Case 1 PSF^{-} history

September 2024: The owner contacted trainer Saskia Brieger for corrective training. The horse was described as spooky, difficult to handle, frequently shying under saddle without apparent reason, and diagnosed as an idiopathic headshaker. It forcefully resisted contact toward the right rein and exhibited dysbalanced movement. The owner was unable to ride him. The horse showed strong pain reactions in the poll, cervical spine, and cervicothoracic junction. He barely tolerated saddling and bridling. Additionally, an ataxic hindlimb landing pattern was noted, together with poor hoof care and thrush in all four hooves.

3.1.2.2 Case 1 clinical findings

No clinical diagnostics were performed prior to the training intervention, as the previous owner interpreted the headshaking, shying, and resistance under saddle primarily as behavioral issues rather

than potential indicators of a physical condition. In leisure horses this is common, particularly when an initial veterinary examination does not reveal a clear pathology and more extensive diagnostic work-ups exceed the owner's practical or financial scope. Consequently, no medical findings are available for the period before October 2024.

3.1.2.3 Additional data on Case 1

Hoof Data from June 2025:

- Left front/right front: Hooves even, shod, narrow heels, dorsal wall angle >50°, coronary band angle ~20°, underdeveloped frog, thrush present.
- Left hind/right hind: Hooves even, unshod, long toe, underrun heels, coronary band angle >30°, suspected negative plantar angle, severe thrush right hind.

3.1.3 Case 1 development after introduction to PSF$^+$

3.1.3.1 Case 1 PSF$^+$ history

October 2024: The horse showed marked improvement after a short period of corrective training with the new trainer Saskia Brieger, who introduced the first functional principles later formalized in FIT. This initial reorganization was sufficient to make the horse rideable again for the owner. In late October, an invasive manual intervention by an external practitioner led to a dramatic relapse with pronounced headshaking, increased muscle tension, and renewed behavioral instability. Case 1 showing increased muscle tension and pain signs is displayed in Supplementary Figure 1.

November 2024–March 2025: Case 1 is unrideable and shows dangerous behavior triggered by cold air. Therapeutic groundwork only.

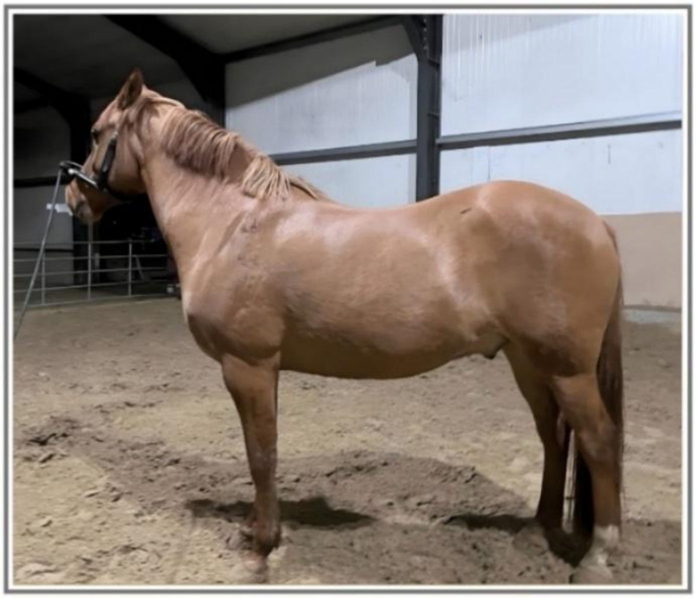

Supplementary Figure 1. *Case 1 in a relapse to PSF⁻ after an intense manual treatment at the end of October 2024.*

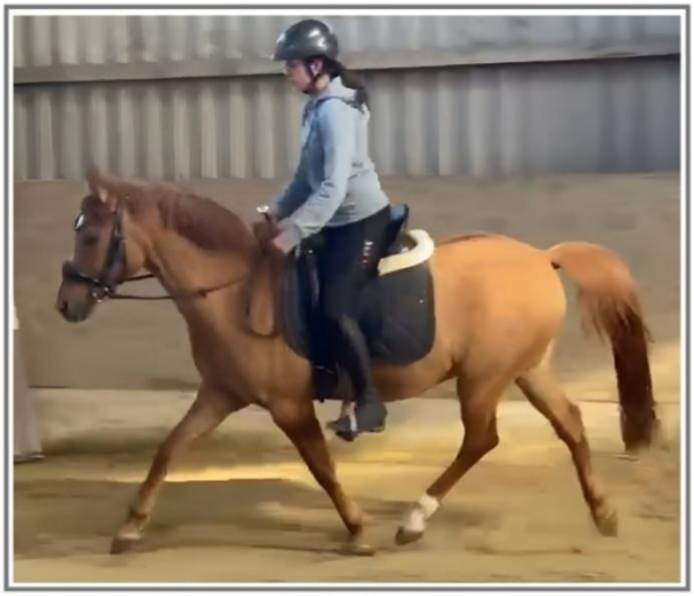

Supplementary Figure 2. *Case 1 in one of the first short riding sessions with FIT approach in March 2025 with one of the owners. The shifted stance phase improved but was still visible.*

Late March 2025: Mixed training approach—introduction of FIT in addition to continued and adapted therapeutic training. First short, successful riding sessions with improved gait phases as displayed in Supplementary Figure 2; saddling improved; still pronounced headshaking with triggers such as rain and wind.

July 2025 and thereafter: following a change of ownership, the horse was trained exclusively with FIT within the PSF^{+} framework (Supplementary Figure 3).

3.1.3.2 Current status Case 1

The results achieved as of November 2025 are:

- The horse is free of headshaking (last time recorded in September 2025 during light drizzle).
- Completely pain-free, poll and cervical spine unremarkable, no more sensitivity at cervicothoracic junction and lumbosacral joint
- No longer sensitive when saddled or bridled
- Fully rideable again, even for less experienced riders, both in arena and outdoors (see Supplementary Figure 4–Supplementary Figure 6)
- Improved stability and balance
- Significant improvement in hindlimb landing pattern
- No more thrush
- All hooves have widened considerably and are back to normal. The coronary band is ~30° on all four hooves
- Overall improved health, and quality of life

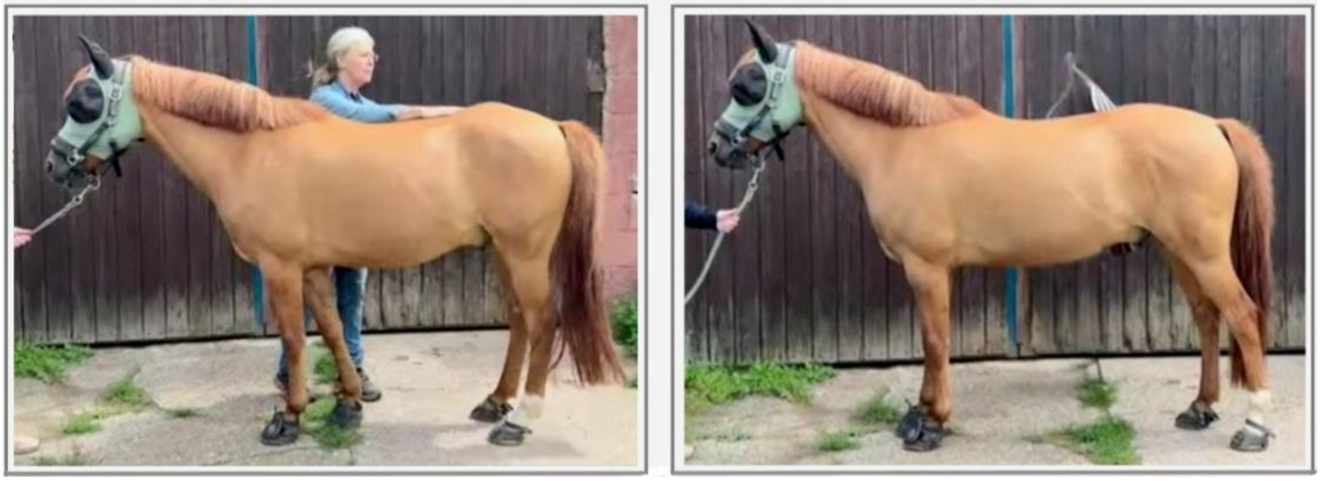

Supplementary Figure 3. *This is the unedited raw data of Figure 2 in the main text (Photograph taken in September 2025). In the left image, Case 1 adopted its stance voluntarily, showing an open lumbosacral joint. In the right image, the lumbosacral joint was functionally closed through manual stimulation of specific trigger points, after which Case 1 assumed the new posture of its own accord.*

3.1.4 Case 1 conclusion

This Case Study 1 demonstrates that substantial restoration of postural stability, rideability, and comfort can be achieved by riders of all experience levels when guided by trainers who have integrated a PSF^{+}-based approach. Although the trainer in this case had long-standing teaching experience, she had been working with FIT for less than a year before July 2025, when the training of Case 1 began to show lasting success. The rapid reduction of pain responses and disappearance of headshaking without any medical or therapeutic intervention supports the view that many pathological manifestations may resolve spontaneously once dysfunctional movement patterns are replaced by coordinated, self-organized motion. These findings highlight that systemic reorganization through functional movement

is a key to restoring structure and function across a wide range of initial conditions, providing an accessible and sustainable path toward long-term equine health and performance.

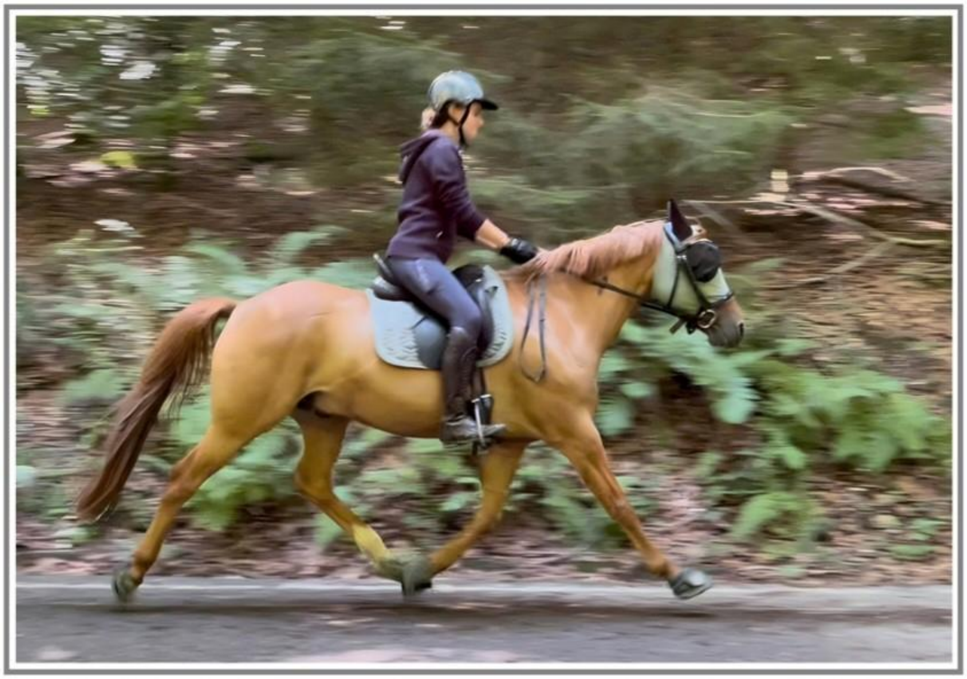

Supplementary Figure 4. *Case 1 in full PSF$^+$ state, outdoors in trot, persistent and dedicated, no more phase shift visible in July 2025.*

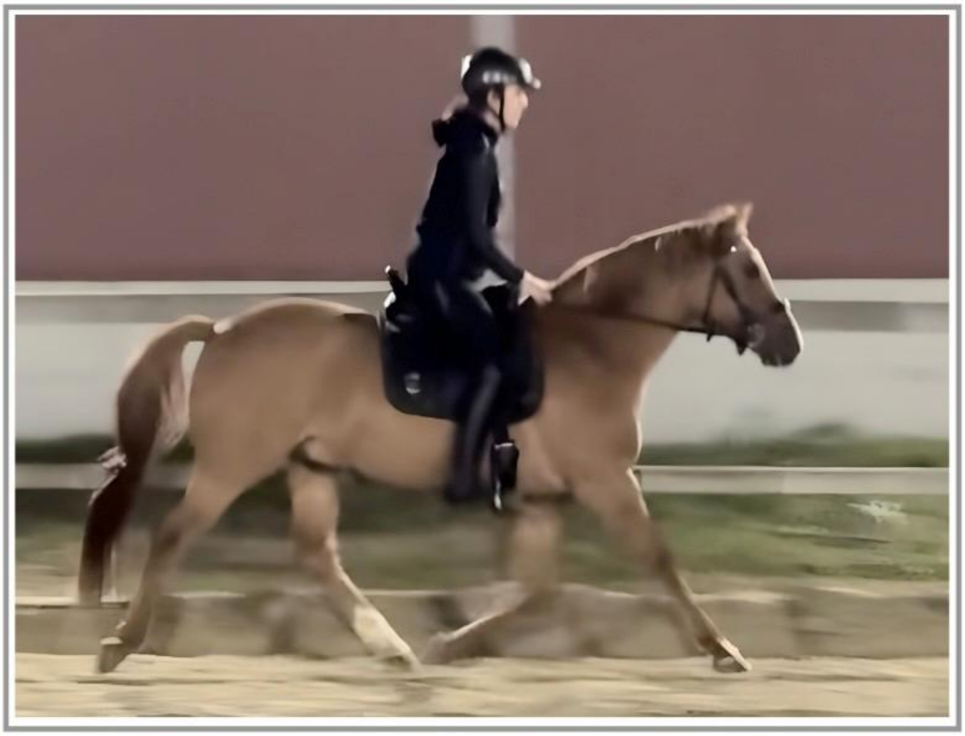

Supplementary Figure 5. *Case 1 in full PSF^{+} state, in the riding arena in October 2025, cooperative, willing to perform and adjustable.*

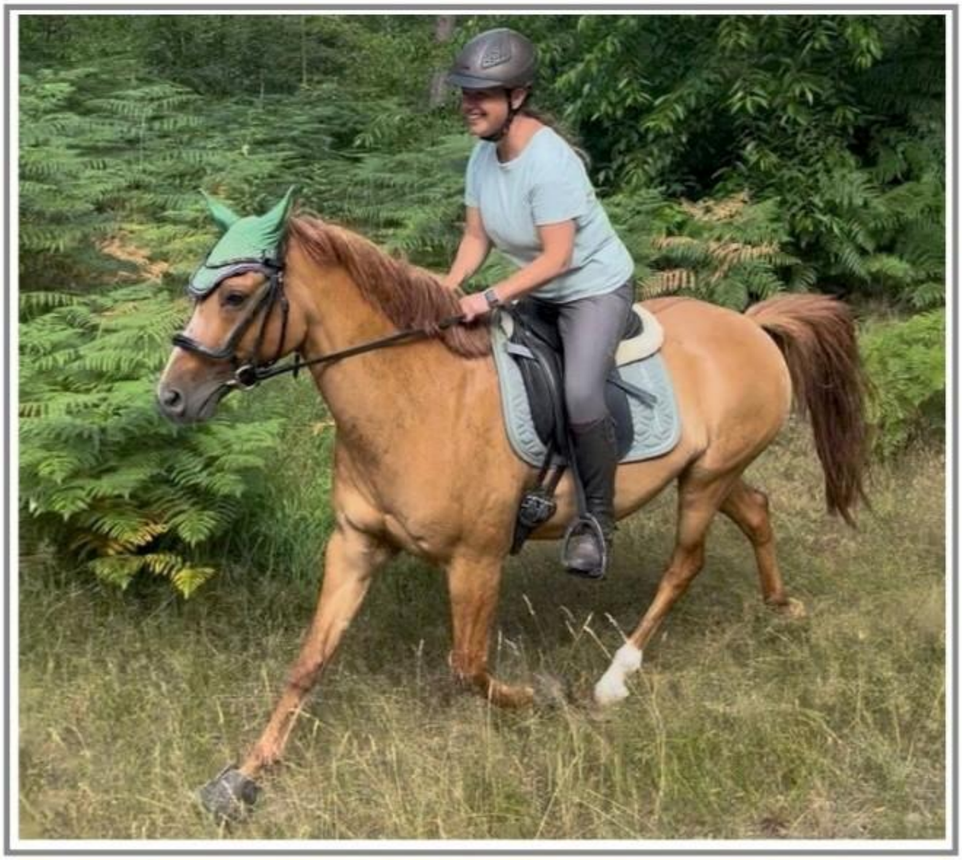

Supplementary Figure 6. *Case 1 in full PSF$^+$ state, June 2025—hacking with a stable, reliable and engaged horse.*

3.2 Case Study 2: Progressive Structural and Functional Change in a Horse with Severe ECVM

Case 2 represents a subgroup of horses with severe congenital or structural conditions such as Equine Complex Vertebral Malformation (ECVM), for which conventional medical and therapeutic approaches often yield only temporary improvement.

The Oldenburg gelding in this case presented with multiple cervical malformations, recurrent colics, gastric ulcers, right hindlimb lameness, and generalized instability. Despite controlled exercise and extensive veterinary treatment, including platelet-rich plasma injections, sustained improvement was not achieved.

From late 2023 onward, training and hoof care were transitioned to the PSF^+ approach of FIT, emphasizing structural balance, functional motion, and self-organization. Within one year, the horse exhibited stable recovery: colic episodes ceased, gastric ulcers healed, and postural symmetry, musculature, and coordination improved markedly. Even after a seven-month training pause, functional development continued, indicating a lasting systemic adaptation.

Data were collected from veterinary diagnostics, radiographic follow-ups, and longitudinal owner documentation between 2021 and 2025. Hoof geometry and lameness development were monitored in parallel.

This case demonstrates that, even in horses with severe congenital malformations and limited therapeutic options, the PSF^+ cycle, once initiated, can promote long-term functional regeneration through self-organization rather than compensatory management. Since the horse always had been trained predominantly cross-country, improvements cannot be attributed alone to work outdoors being favorable over arena work but indicate that the quality of movement is paramount. In this case, the transition from PSF^- to PSF^+ following the introduction of FIT in combination with the change to barefoot management represents the most plausible turning point in the horse's development.

3.2.1 General information on case study 2 subject

Case Identifier: Case 2
Date of birth: 03.05.2018
Breed: Oldenburg
Sex: Gelding

3.2.2 Case 2 PSF^- development

3.2.2.1 Case 2 PSF^- history

February 2021: The horse was started under saddle, mostly in the countryside alternating led and ridden, some free jumping, occasionally lame.

February 2021 & January 2022: Two severe impaction colics requiring hospitalization.

May 2022: Slight right hindlimb lameness at the beginning of warm-up, usually disappearing after a short period of work. Osteopathic treatment: After releasing a trigger point in the gluteal muscle, loss of control over the right hindlimb occurred and subsequent referral to Clinic 1 (anonymized), which

started a series of veterinary investigations (clinical findings section). As a result of the diagnoses found, movement and training were severely restricted due to the high risk of falling.

November 2022: Platelet-rich plasma (PRP) therapy resulted in improvement, but not full rehabilitation.

Winter 2022–Summer 2023: Recurrent lameness episodes, gastric ulcers, repeated colic, and poor general condition as depicted in Supplementary Figure 7. Between the lameness episodes, the horse was ridden at walk in the countryside

Autumn 2023: Therapeutic training introduced, slight improvement observed.

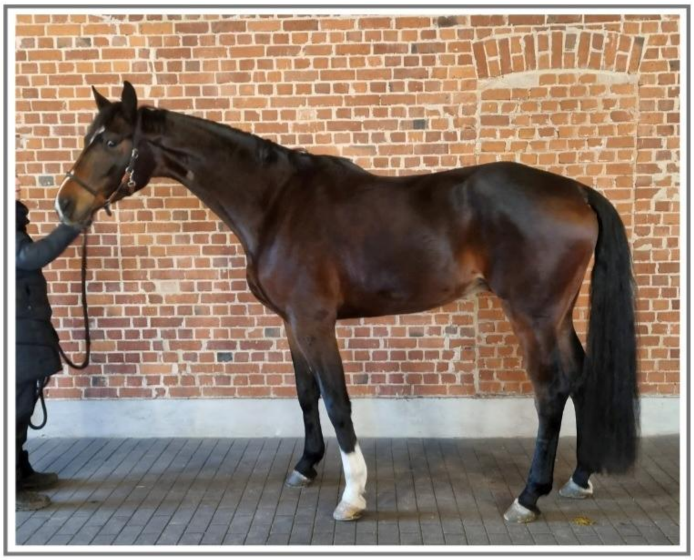

Supplementary Figure 7. *Case 2 in February 2023 in clear PSF^{-} state–unstable and in poor general condition after several colics and gastric ulcers.*

3.2.2.2 Case 2 clinical findings

a. Lameness examination in Veterinary Clinic 1 (anonymized; May/June 2022)*

- Clinical Examination:
  - Highly pressure-sensitive/tender in the saddle position
  - Right stifle mildly swollen, otherwise unremarkable
  - Sound at trot on a straight line; unclear, mild lameness right hind on circle, especially on left hand
  - Slightly uncoordinated movement pattern
  - Flexion tests hind limbs negative bilaterally
- Nerve Blocks (RH):
  - Low four-point block: negative
  - Plantar nerve block: ~50% improvement
  - Tibial/fibular block: clearly positive, slight residual lameness
  - TMT block: negative
- Imaging:

  - Radiographs: changes in axis, occiput, C4/C5, C7/T1; angular deviation C4/C5; reduced intervertebral space C7/T1; extension of the dorsoproximal metacarpal canal (RH); mild to medium inhomogeneity in origin of suspensory ligament (RH)
  - Ultrasound: thickening at origin of suspensory ligament (RH)
- Suspected Diagnoses / Recommendations (not followed by the owner):
  - CT of neck
  - Symptomatic therapy with corticosteroids and DMSO
  - Stable rest & controlled exercise as well as shockwave therapy at suspensory origin

b. ***Secondary evaluation of diagnostic images by Veterinarian 1 (anonymized; June 2022)****

- Diagnosis: Severe ECVM, see Supplementary Figure 8
  - C6: bilateral complete absence of ventral laminae
  - C7: bilateral transpositions of ventral laminae
  - Rudimentary first rib articulations bilaterally
  - Bony proliferations on occiput, step formations C2–C4 and C7–T1
  - Missing spinous processes C6 and C7, insufficient development of T1
- Recommendations:
  - Movement restrictions due to risk of falling
  - Neurological examination including ridden assessment to determine rideability
  - Feeding: anabolic nutritional support.

c. ***Lameness examination by Veterinarian 1 (anonymized; June 2022)***

- Severe cartilage lesions in the right hock joint diagnosed

d. ***Neurological examination by Veterinarian 1 (anonymized; June 2022)***

- Severely reduced cervical sensation extending into the forelimbs; asymmetries in saddle position; marked pain sensitivity on the right side of the saddle area
- Riding was not recommended due to the risk of falling

3.2.2.3 Additional data on Case 2 in PSF^{-} state

Hoof data (August 2024):

- LF: Coronary band angle ~22°, tilted outward ~3°
- RF: Coronary band angle ~13° (club foot), tilted outward ~3°
- LH: Coronary band angle ~30°, tilted outward ~3°
- RH: Coronary band angle ~30°, tilted outward ~2°

Dental status:

- Marked overjet with step formation; requires dental correction every 3–4 months

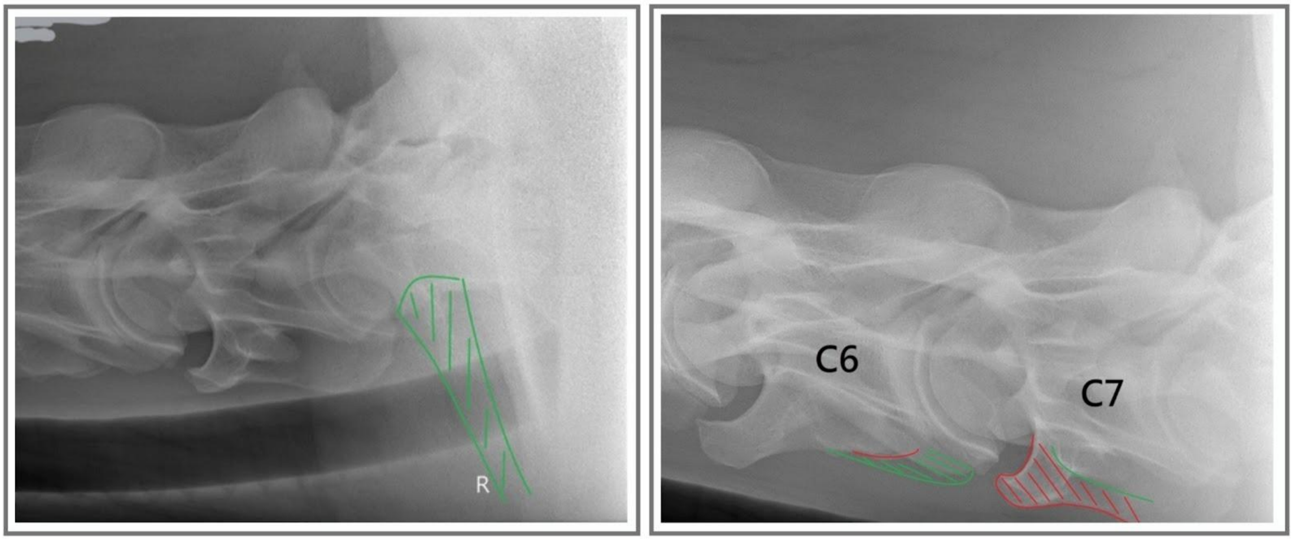

Supplementary Figure 8. *Radiographic findings ECVM (X-rays by Clinic 1, interpretation and drawings by Veterinarian 1). The left image shows a rudimentary first rib, which terminates prematurely compared to its expected anatomical extension (illustrated in green). In the right image, missing bone structures at C6 are highlighted in green shading, and transposed elements at C7 are marked red, indicating abnormal morphology*

3.2.3 Case 2 development after introduction to PSF$^+$

3.2.3.1 Case 2 PSF$^+$ history

November 2023: Owner began independent practice based on Maren Diehl's publications.

February 2024: Start of guided FIT with Saskia Brieger and Maren Diehl.

Although training was paused from July 2024 to February 2025 because of an accident of the owner, the horse showed a remarkable functional and postural development according to PSF$^+$ after this period (see Supplementary Figure 9):

- Rapid and stable improvement in movement organization
- Progressive increase in musculature, soundness, evident hindlimb engagement and impulsion
- Weight gain, no further colics or gastric ulcers
- Improved cervical and forelimb proprioception
- Gate asymmetries and phase shifts are no longer noticeable (before and after see Supplementary Figure 10)
- The horse appears more stable, confident, and willing to perform

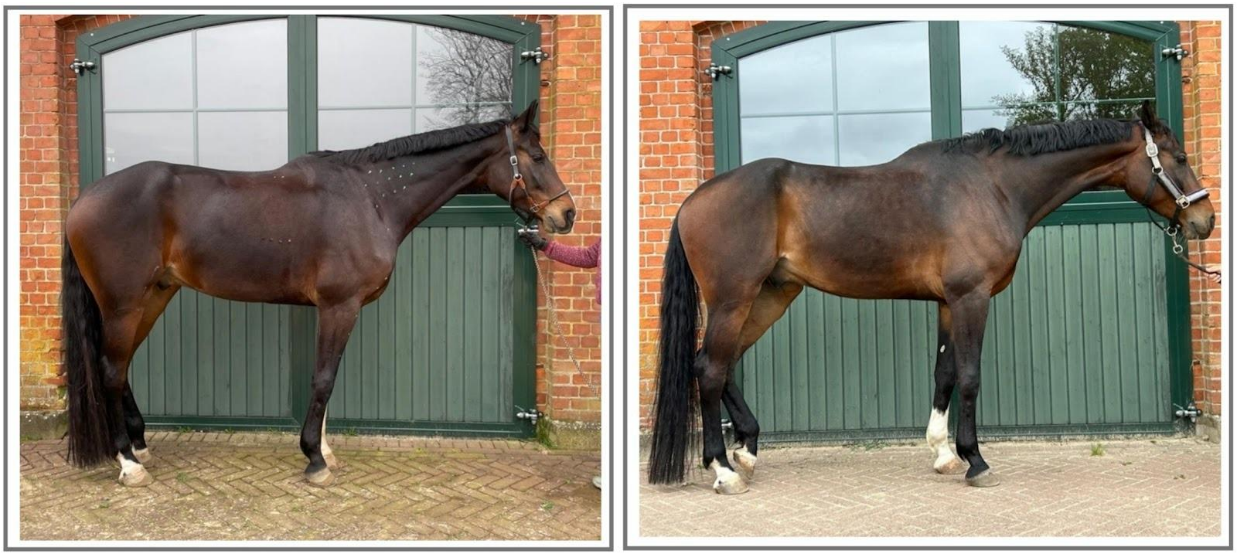

Supplementary Figure 9. *The left panel shows Case 2 in March 2024, at the very beginning of FIT and still in PSF⁻, the right panel shows Case 2 in May 2025 in PSF⁺. These photographs were taken at the same place from nearly the same perspective with approximately the same distance to the doors. The height of the withers in both pictures can be compared by the window frames.*

3.2.3.2 Current status Case 2

Current status (fall 2025, see Supplementary Figure 11):

- The gelding is sound, well-muscled, and shows strong hindlimb engagement and good overall fitness.
- No further issues with colic, gastric ulcers or neurological anomalies
- The right hindlimb remains a minor weak point and indicator for potential overload, but no persistent lameness is present.
- Overall impression: clearly improved stability, health, and quality of life
- Hoof data: Hoof care performed by a professional barefoot trimmer:
    - All hooves have widened about 1cm, one more cm is aimed at
    - LF: Coronary band angle ~28°, no outward tilt,
    - RF: Coronary band angle ~25°, no outward tilt,
    - LH: Coronary band angle ~30°, no outward tilt,
    - RH: Coronary band angle ~30°, no outward tilt,
    - The difference in coronary band angles between the right and left front hoof decreased from 9° to 3°.

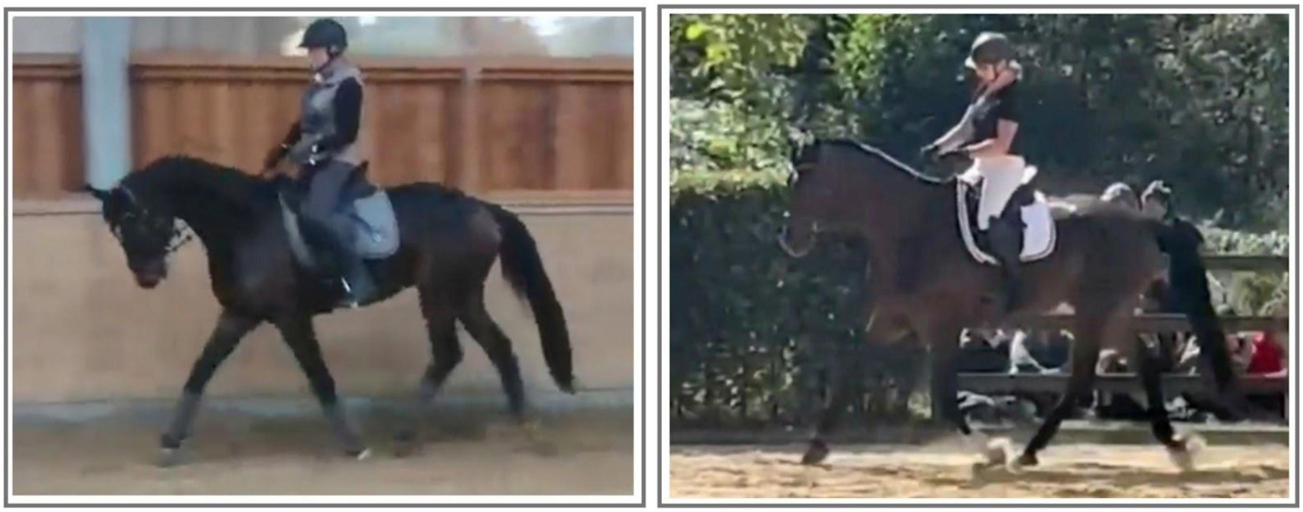

Supplementary Figure 10. *The left panel shows Case 2 in "forward and down" training in February 2023 with delayed lift-off in the forelimb, right panel shows Case 2 at an event in August 2025 in PSF⁺ with synchronized diagonal feet.*

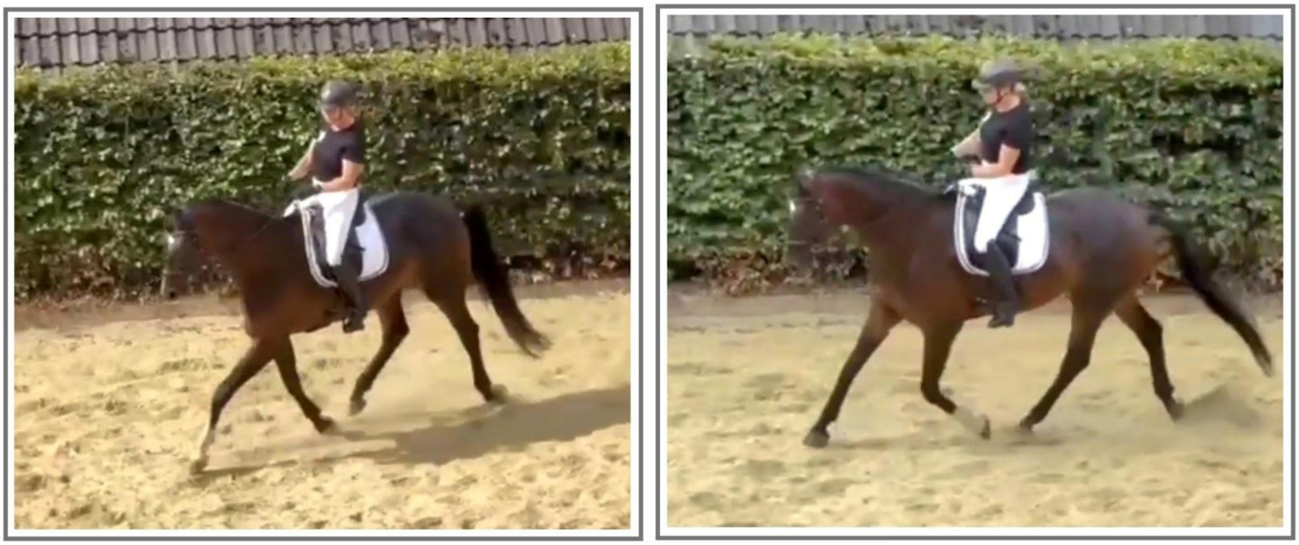

Supplementary Figure 11: *Both panels show Case 2 on the same event as in the right panel of Supplementary Figure 4 in August 2025. Both diagonals show simultaneous lift-off at the end of the stance-phase, although the horse was in a state of high agitation.*

3.2.4 Conclusion Case 2

This case demonstrates that even horses with severe congenital malformations, long-standing instability, and complex clinical histories can achieve sustained improvement when training and management are aligned with the PSF^+ framework. Beyond the restoration of postural stability, gastrointestinal function, and coordinated movement, the integration of hoof data provides objective evidence of systemic reorganization: over the course of FIT and physiologically guided trimming, the previously pronounced disparity between the steep club foot and the flat contralateral hoof diminished markedly from 9° to 3°, and the former outward rotation fully normalized.

The consistent pattern of change—across locomotion, posture, gastrointestinal health, and hoof geometry—supports the interpretation that these findings represent interconnected manifestations of PSF^{-} rather than isolated defects. The PSF framework enabled a clearer identification of the functional control points underlying this horse's instability and provided a coherent rationale for intervention across systems.

By approaching the problem systemically rather than symptomatically, the PSF^{+} cycle offered a replicable pathway toward functional recovery: improved load management, restored movement efficiency, and progressive structural convergence of the hooves emerged not as spontaneous resolution, but as predictable outcomes of restored interaction with external forces and renewed self-organization within the body.

3.3 Case Study 3: Progressive Structural and Functional Change in a Warmblood Gelding with Recurrent Suspensory Ligament Injuries and Gastric Ulcers

Case 3 represents a group of sport horses developing recurrent gastric ulcers and soft-tissue injuries as secondary manifestations of PSF⁻.

The gelding, purchased as a seven-year-old sport horse, showed behavioral resistance, recurrent gastric discomfort, and two consecutive suspensory ligament lesions under conventional training. Conventional veterinary and osteopathic treatments provided only temporary relief, and early retirement was considered.

Data were collected from veterinary diagnostics, longitudinal owner documentation between 2020 and 2025. Hoof geometry and lameness development were monitored in parallel

After the training focus shifted toward dynamic balance, horizontal bit interaction, and systemic postural organization, the horse regained rhythm, stability, and willingness within approximately six months. From that point onward, no further signs of gastric discomfort were observed, and the horse returned to being sound, confident, and capable of competing. Over the following year, continued emphasis on postural self-organization resulted in normalized hind-limb alignment, a calm mental state, and sustained soundness under regular athletic workload, including participation in competitions. This case demonstrates how restoring functional self-organization can re-establish performance and resilience in horses previously maintained in PSF⁻ patterns despite professional training and veterinary intervention.

3.3.1 General information on case study 3 subject

Case Identifier: Case 3
Date of birth: 16.06.2012
Breed: Rheinländer (Warmblood)
Sex: Gelding

3.3.2 Case 3 PSF⁻ development

3.3.2.1 Case 3 PSF⁻ history

October 2019: The gelding was purchased as a seven-year-old sport horse by his current owner, at that time being a 13-year-young amateur sports rider. The pre-purchase examination was without findings except for mild O-legged conformation of the hindlimbs.

Summer 2020: First occurrence of Gastric issues.

October 2020: First gastroscopy performed, showing ulceration. Treatment with Omeprazole and Sucralfate was followed by six weeks of rest.

Spring 2020–Fall 2023: The horse received twice-weekly lessons with an FN-licensed Sports Trainer ("Trainer C Reiten") with focus on "low, deep and round" (LDR-posture, see Supplementary Figure 12, left) interrupted by health-related breaks. During this period, severe behavioral resistance developed: kicking at the rider's leg, saddle aversion and girth intolerance, resistance during mounting,

intense and hectic chewing on the bit, and episodes of bucking. The trainer reported "poor performance attitude" and recommended mechanical restrictions such as knots in the martingale and a tightened noseband. During this period, the horse was treated by an osteopath approximately every three months.

December 2022: The first suspensory ligament lesion occurred, leading to a rest period.

April 2023: Work was resumed as previously described with focus on LDR-posture (see Supplementary Figure 12).

May 2023: Second suspensory ligament lesion. The horse also showed unphysiological lateral rotation of both hocks in movement, frequent bucking, and increasing unreliability under saddle. Conventional veterinary treatment and repeated osteopathic interventions brought only short-term relief.

By late summer 2023, early retirement of the horse was being considered.

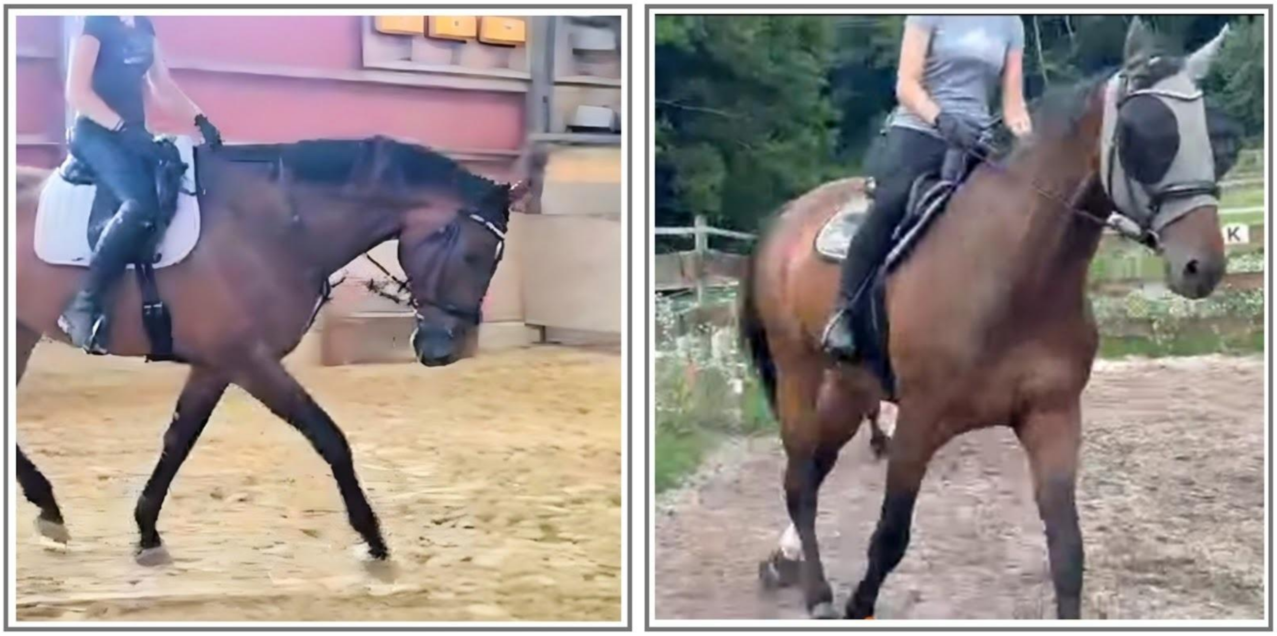

Supplementary Figure 12. *The development of case 3 from PSF^{-} (left) in Summer 2023, to PSF^{+} (right) in Summer 2025.*

3.3.2.2 Case 3 clinical findings

a. Gastroscopy in Veterinary Clinic 1 (anonymized; October 2020)*

- Gastroscopy Findings (Supplementary Figure 13):
  - Ulceration
  - Treatment with Omeprazole and Sucralfate
  - Recommendation to six weeks of rest (followed by the owner)

b. Lameness examination by Veterinarian Clinic 2 (anonymized; December 2022)

- Diagnosis: suspensory ligament lesion (hind left)
- Recommendations:

- 5 days Meloxicam
- 6 weeks 1–2 times a day up to 60 minutes of walk
- Twice a day ICE vibe gaiters

c. ***Lameness examination by Veterinarian Clinic 2 (anonymized; May 2023)****

- Diagnosis: suspensory ligament lesion (front left)
- Recommendations:
  - 3 weeks 1–2 times a day up to 60 minutes of walk in hand or ridden

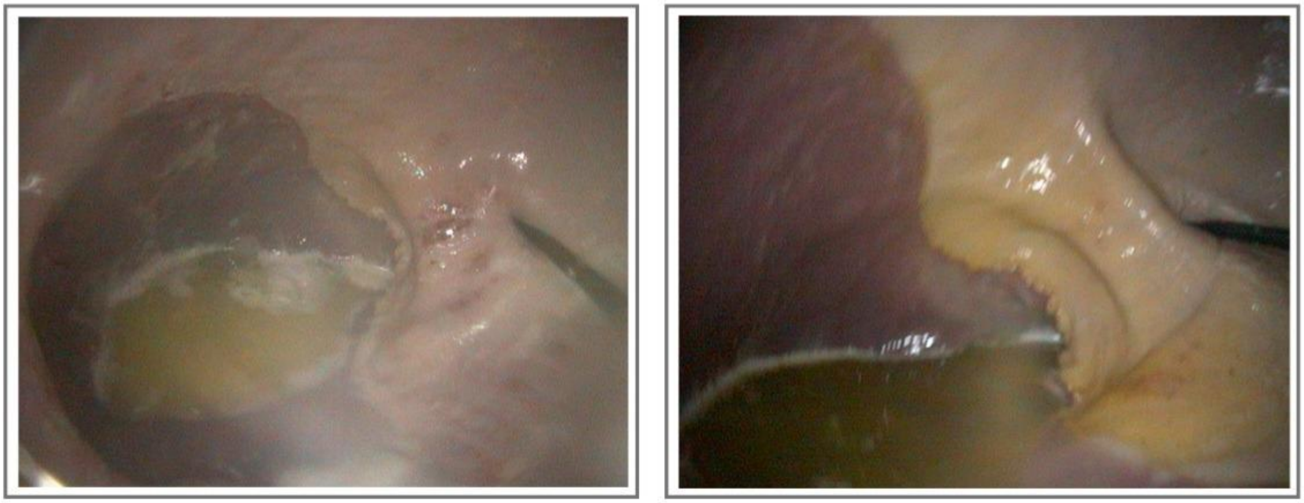

Supplementary Figure 13. *Supplementary Figure 2. Gastric ulcer (left) and after six weeks of treatment with Omeprazole and Sucralfate (right).*

3.3.3 Case 3 development after introduction to PSF$^+$

3.3.3.1 Case 3 PSF$^+$ history

October 2023: Return to the PSF$^+$ Cycle via FIT with short sessions (15 minutes), alternating between groundwork and light riding on firm ground. The focus was on rhythm, balance, and coordination rather than muscular effort. The rider learned to establish functional horizontal connection to the bit and to support postural organization instead of aiming for the LDR-posture.

November 2023–January 2024: Increasing session lengths up to one hour including all gaits. The gelding developed a visibly steadier rhythm, better weight distribution, and a calm, forward attitude. No further gastric discomfort occurred.

March 2024: Hacking became possible without resistance or bucking.

April–May 2024: Re-establishment of the athletic career with a first competition in dressage test (German Dressage-A-level, see Supplementary Figure 14) and a victory in show-jumping (German A-level). The latest osteopathic treatment in 2024 was without any findings.

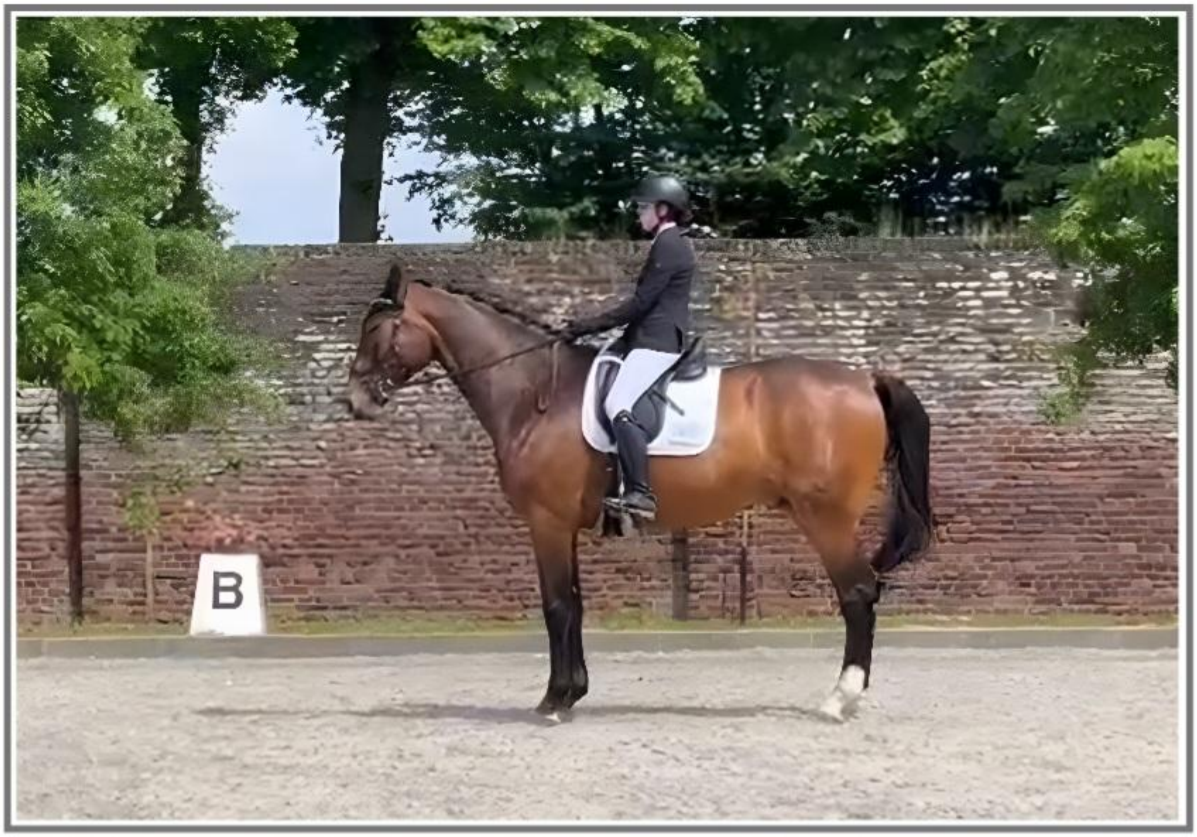

Supplementary Figure 14. *Case 3 in a dressage competition (April 2024). This image shows raw data to the right panel of Figure 7 in the main text. The horse appears in good general condition, although not yet fully in PSF*$^{+}$ *state which would be indicated by four vertical cannon bones in contrast to the slightly underneath the body positioned limbs here. Note that this picture was taken at the end of the movement in halt, under consistent horizontal tension applied to the bit.*

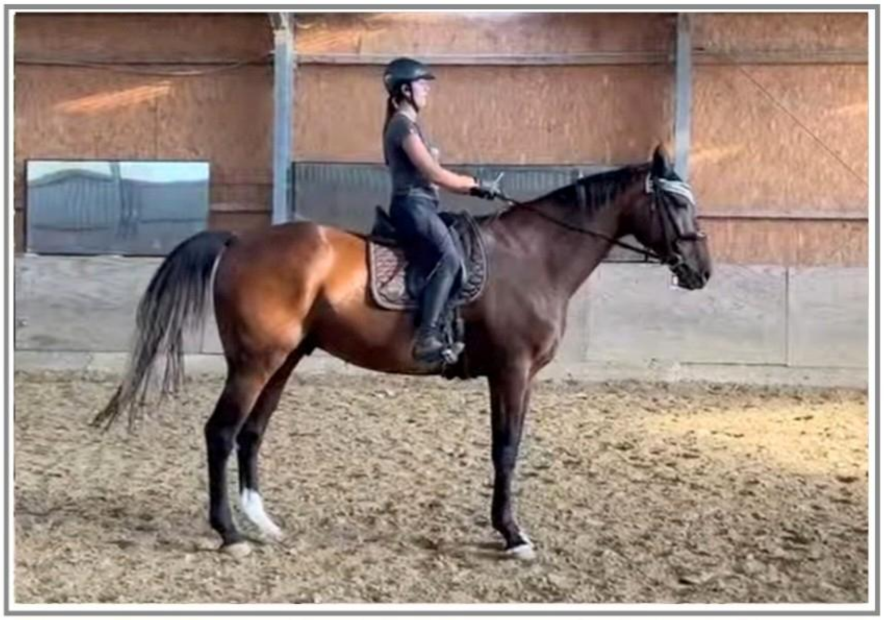

Supplementary Figure 15. *Case 3 in almost fully restored PSF^{+} state (August 2025). This is the raw image corresponding to the left panel of Figure 7 in the main text. The horse's conformation has changed favorably: the cannon bones are nearly vertical, and rotation of the hocks during motion has markedly decreased. Note that this picture was taken at the end of the movement in halt, under consistent horizontal tension applied to the bit.*

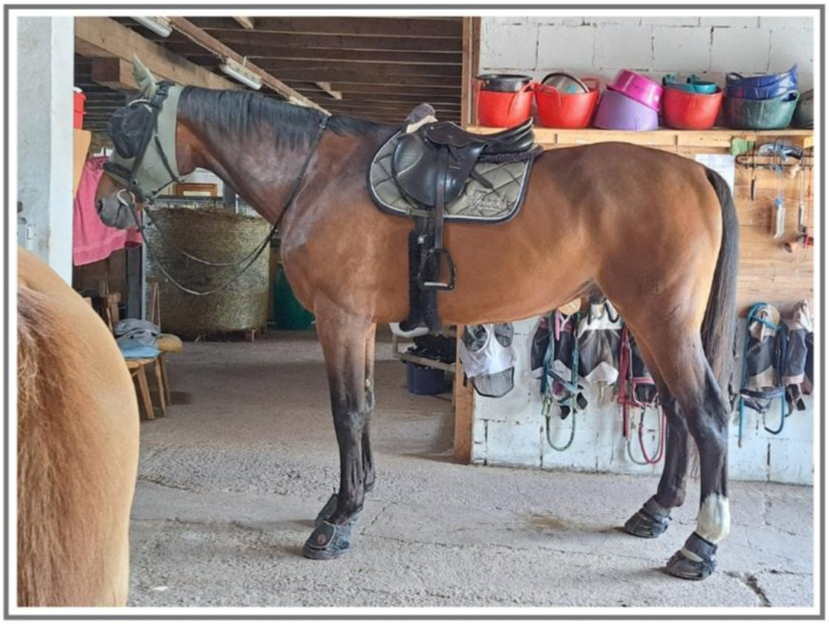

Supplementary Figure 16. *The gelding during an active holiday, following several days of hacking across gently rolling terrain. Compared to the arena situation shown in Supplementary Figure 14, the forelimb joint angles are noticeably wider (elbow approximately ~135° vs ~120°).*

June 2024–October 2025: Training continued to follow the principles underlying FIT. The young owner trained independently, and the gelding stayed completely sound and reliable, competing regularly in dressage and show-jumping events. Regular hacking and holiday trips to hilly terrain further improved his coordination and mental relaxation. Hock rotation during movement reduced markedly. By late summer 2025, conformation photographs revealed nearly vertical cannon bones (Supplementary Figure 15 & Supplementary Figure 16).

In March 2025, hoof care was changed to another concept, improving symmetry in the hind hooves. The right hind medial wall remained slightly higher, while the left hind hoof is functionally balanced.

3.3.3.2 Current status Case 3

October 2025: The gelding is fully rideable and in active competition (Supplementary Figure 17), both in arena work and on outdoor tracks. Overall, the horse is in excellent condition, stable, cooperative and well-muscled, and the difference to the time of purchase is remarkable (Supplementary Figure 18).

3.3.4 Case 3 conclusion

This case illustrates how a targeted shift in training focus—applied here through principles consistent with FIT—can support the reversal of PSF^{-} patterns and facilitate lasting PSF^{+}. By restoring coordinated self-organization, balanced load distribution, and a stable rhythm, the gelding transitioned from behavioral resistance and recurrent soft-tissue injury to sustained postural stability and athletic soundness, as shown in Supplementary Figure 17. This outcome demonstrates that functional recovery and long-term performance can emerge without continuous therapy or conventional performance training, highlighting the systemic regenerative capacity inherent in the PSF^{+} process when training aligns with physiological principles.

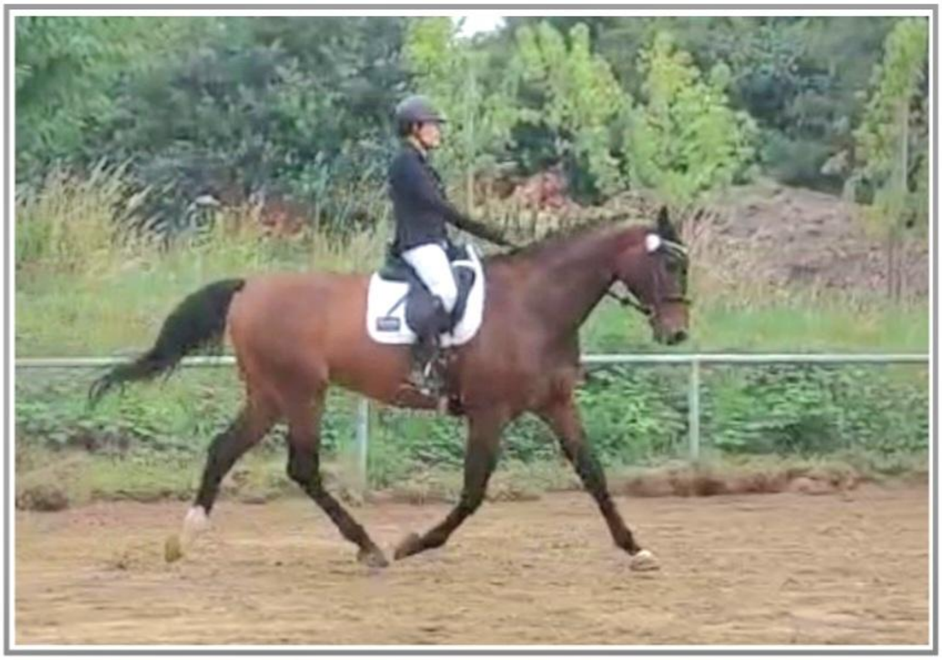

Supplementary Figure 17. *Case 3 in a dressage competition in July 2025 in a clear suspension phase in medium trot.*

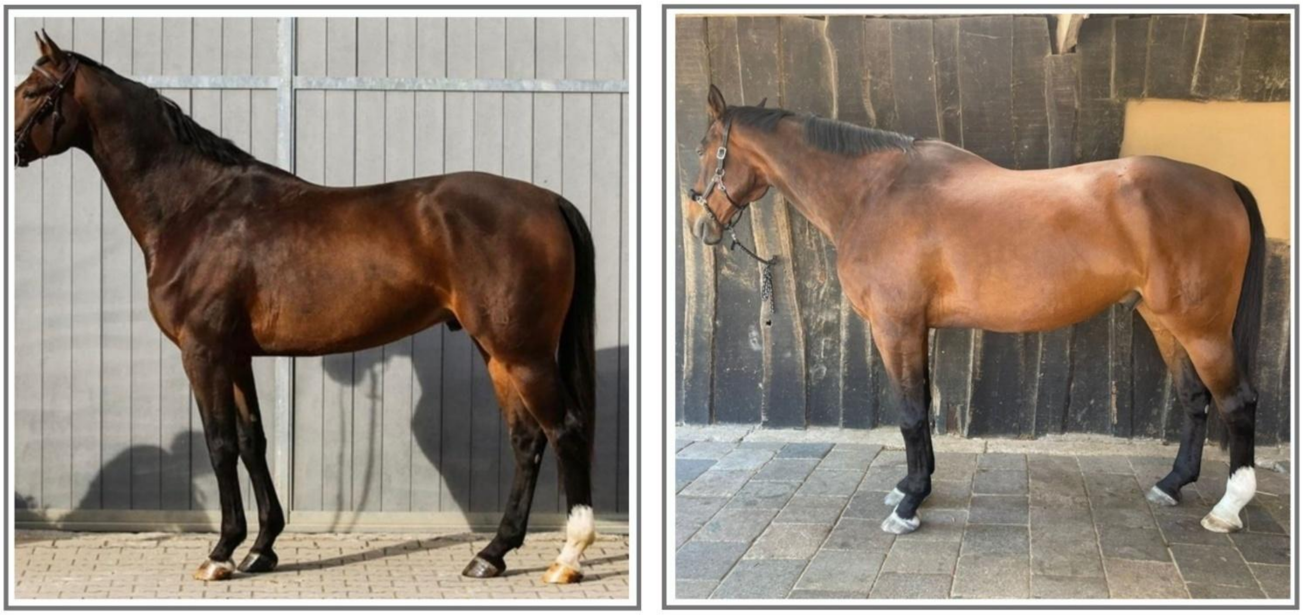

Supplementary Figure 18. *Comparison of the horse's conformation at the time of purchase in 2019 (left) and the current state in autumn 2025 (right).*

3.4 Case Study 4: Progressive Structural and Functional Gain (PSF^+) in a Former Racehorse with Chronic Pain and Headshaking

Case 4 represents a large group of horses which retired from competitive sport (Supplementary Figure 19) and transferred into leisure riding. Many of these horses carry lasting physical strain and psychological tension from their athletic careers and struggle to adapt when no longer required to perform. Caused by the lack of challenges and not being retrained into a state compatible with leisure riding, their frustration, pain and discomfort often manifest as dangerous aggression rather than resignation. At the beginning of observation, the Thoroughbred gelding Case 4 was unrideable and aggressive, showing idiopathic headshaking, generalized pain sensitivity, and severe behavioral dysregulation. Conventional training programs and therapeutic interventions, including osteopathic and professional rehabilitation approaches, had failed to achieve lasting improvement.

Data were collected from veterinary diagnostics, radiographic follow-ups, and longitudinal owner documentation between 2020 and 2025. Hoof geometry and lameness development were monitored in parallel.

Following a shift in training focus and barefoot management that aimed at restoring structural balance and self-organization, the horse underwent a marked transformation. Within one year, pain responses subsided, postural stability improved, and the gelding became calm, cooperative, and reliable under saddle. The disappearance of aggression coincided with the return of physical comfort and functional integrity.

This case exemplifies how systematic reorganization of movement and load distribution within the PSF^+ framework can support the restoration of both physical function and behavioral stability in ex-sport horses that might otherwise remain difficult or unsafe to handle.

3.4.1 General information on case study 4 subject

Case Identifier: Case 4
Date of birth: 02.03.2010
Breed: Thoroughbred
Sex: Gelding
Veterinarian: Dr. med. vet. Maria-Dorothee Faust and others, anonymized
Trainer and Owner: Saskia Brieger

3.4.2 Case 4 $PSF^–$ development

3.4.2.1 Case 4 $PSF^–$ history

January 2021: The horse arrived at the owner's facility in late January as an unrideable and unmanageable ex-racehorse. Previous owners had reported aggressive and dangerous behavior toward humans. He was emaciated, had an old tendon injury on the left forelimb, poor hoof condition including shod high/low forefeet, and showed marked pain sensitivity throughout the thoracic region. He could barely be led, escalated during lunging, and attacked stable staff during feeding and mucking out. Riding was impossible.

After receiving veterinary and osteopathic clearance for training, work began using a therapeutic training aid following a rehabilitation training concept with focus on fascial function regulation and

promoting self-posture of the horse on the loose rein. During this period, it became evident that the horse suffered from severe headshaking, which made training unpredictable and, on some days, impossible.

April/May 2021: Euthanasia was being seriously considered after the horse attacked and bit a fellow boarder during hand-walking.

July 2021: Consistent work with a therapeutic training aid as initially described (four to five sessions per week) began to show progress. The horse became more manageable and cooperative, and riding was possible again. First transition to barefoot management.

May 2022: The owner and Case 4 participated in a training program for instructors of the initially described rehabilitation method and Case 4 was relocated for this purpose. Although he was rideable, the gelding remained unpredictable and difficult to handle. At that time the horse was shod with wedges (forefeet) as recommended by the master instructor.

June 2022–August 2022: Case 4 was sent for three months of professional training of the initially described rehabilitation method, which resulted in no substantial improvement. The gelding remained explosive, continued headshaking, and the master instructor stated that this horse was only trainable to a very limited extent.

September 2022: Back home the horse sustained a severe injury on his left hind leg in an accident in the stable, leading to a 10-month layoff and hospitalization (not separately mentioned in the Clinical Findings Section). During this time, his owner attended several courses with Maren Diehl.

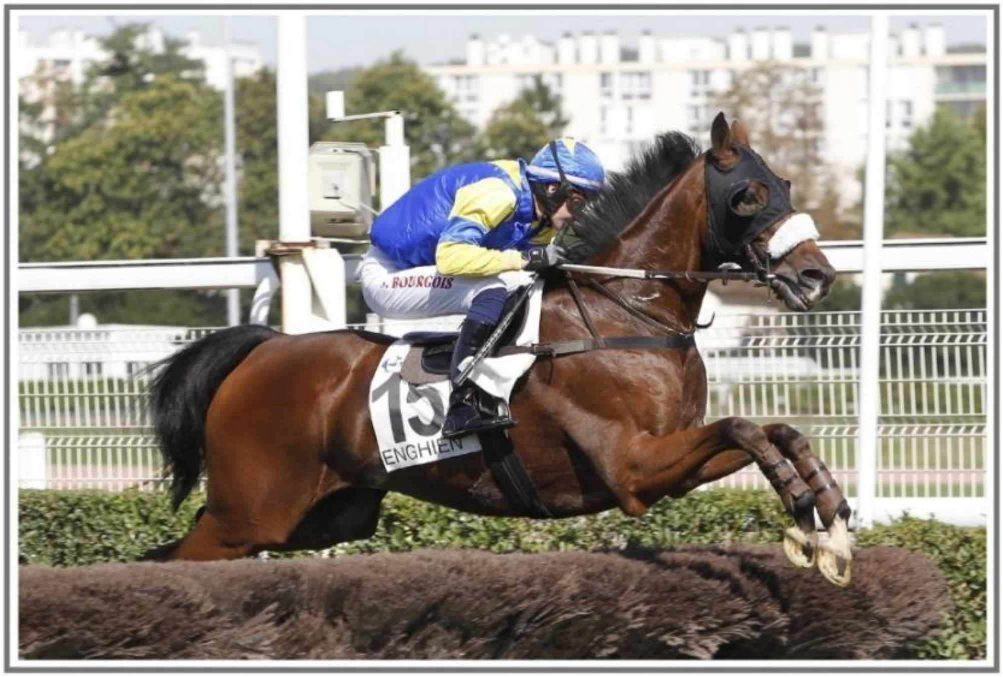

Supplementary Figure 19. *The gelding Case 4 in a steeplechase race in 2017.*

3.4.2.2 Case 4 clinical findings

a. ***General assessment due to behavioral issues, Veterinary Clinic 1 (anonymized; January 2021)***

- Diffuse back pain in the thoracic region without further diagnostics
- Recommendation: proceed with rehabilitation training and re-evaluate if symptoms persist

b. ***ECVM investigation by Dr. med. vet. Maria-Dorothee Faust (June 2024)****

- Radiographic findings in neck:
  - Reduced left lamina on C6, but no ECVM
  - Multiple facet joints with arthrosis
- Additional radiographs of thoracic spine:
  - Old, double spinous processes impingement (Supplementary Figure 20)

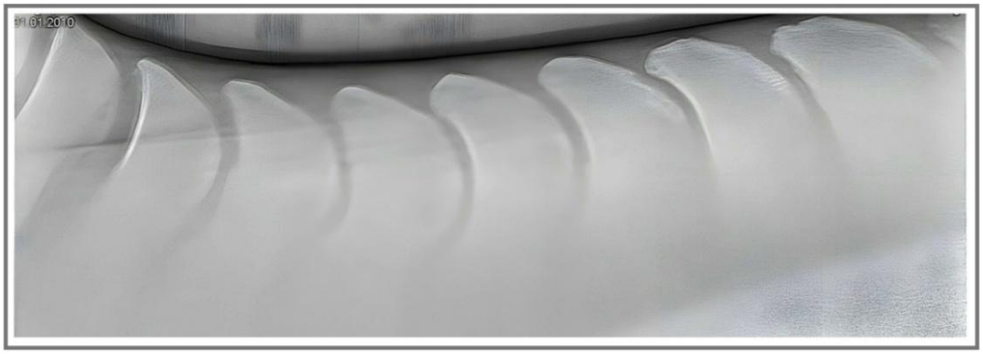

Supplementary Figure 20. *Intervertebral spaces present normal, while former thoracic processes impingements can be deduced from the still visible bone remodeling.*

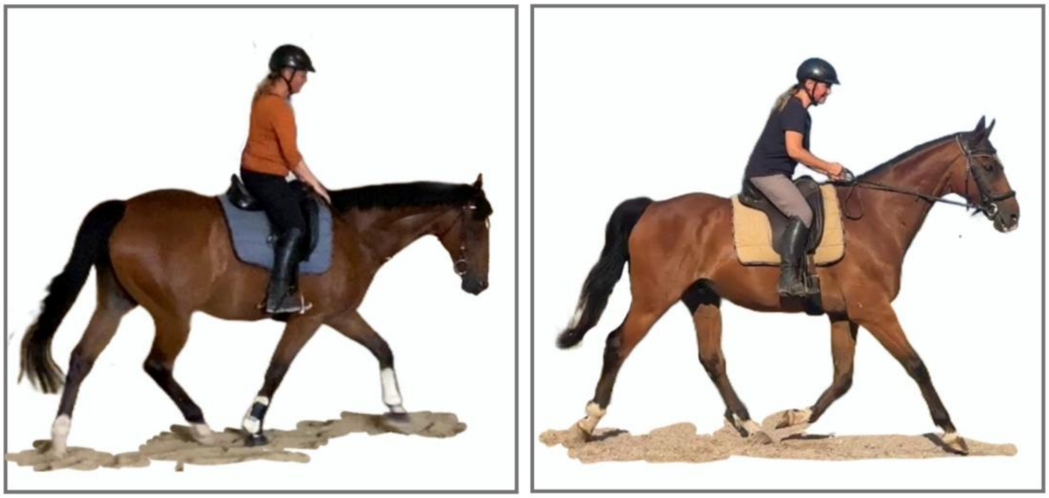

Supplementary Figure 21. *The left panel shows Case 4 in April 2022 in forward and down position, with visible phase-shift and the croup positioned higher than the withers. In June 2024 (right panel)*

the horse is in PSF$^+$-state with a clear horse-initiated forward tension towards the bit, closed lumbosacral joints and almost phase-synchronized fore and hind legs.

3.4.3 Case 4 development after introduction to PSF$^+$

3.4.3.1 Case 4 PSF$^+$ history

July 2023: After its recovery from the major injury in September 2022, the horse was rehabilitated with FIT. This proved to be the turning point in rideability and behavior (see Supplementary Figure 21). He was transitioned to barefoot again and trained with horizontal, horse induced tension to the bit, primarily outdoors. The phase-shift in trot became almost unnoticeable, while range of motion and speed increased and the horse was overall calm and unagitated.

June 2024: Radiographs were taken to check for ECVM. A reduced left lamina on C6, and multiple facet joint arthritic degenerations were found in the neck X-ray images, but no ECVM. In addition, the thoracic spine was investigated, which revealed old, double spinous processes and potential old and already healed impingements (Supplementary Figure 20). However, no current impingements were visible, and only residual bone remodeling at previous contact points remained. The previously described clinical manifestations and behavioral abnormalities (see PSF$^-$ section of this Case Study) are attributed from our side to these findings. With FIT and functional reversion proceeding, Case 4 no more shows clinical symptoms associated with these findings (Supplementary Figure 22).

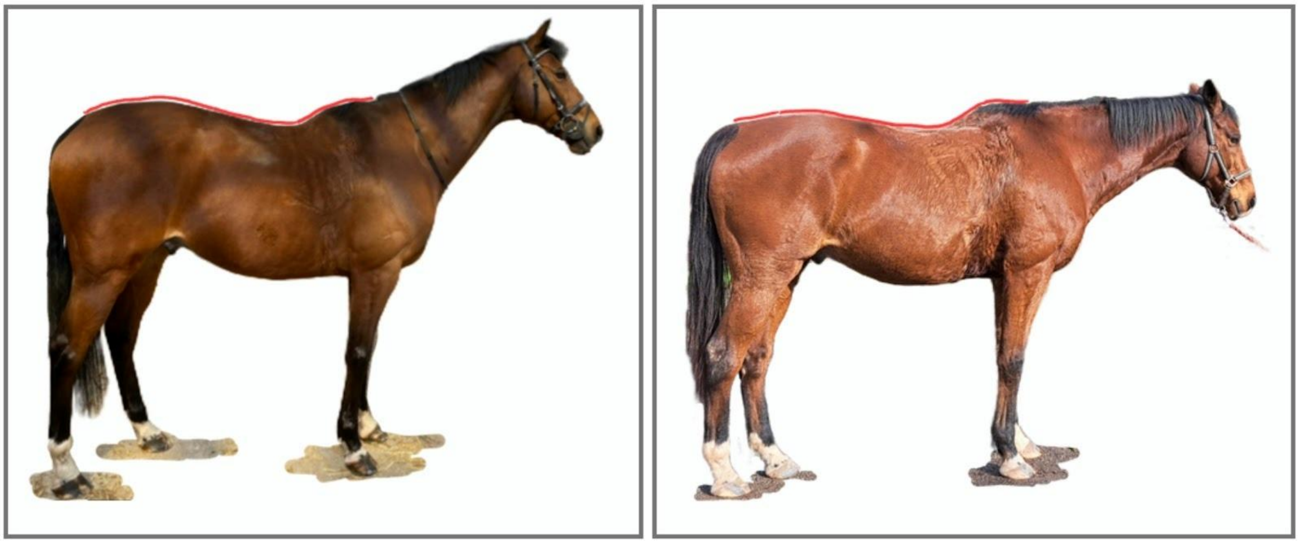

Supplementary Figure 22. *The horse in April 2021 (left) and October 2025 (right). The red line highlights the changes in the horse's back, which, in combination with the previous findings shown in Supplementary Figure 20, may be associated with the horse's former aggressive behavior, pain reactions, and periods of un-rideability. The unhappy face on the right results from a stop and turnaround on the way to the pasture.*

August 2024: Stables were moved and despite this potentially stressful event for a horse, Case 4 remained sound and calm without showing any of the behavioral anomalies described in the PSF$^-$ history of this case study.

September 2024: At the end of the month Case 4 completed a three-day trail ride that marked a major physical and mental transformation. Since then, the gelding has become a motivated, cheerful, and highly reliable horse. Explosiveness, aggression, and headshaking completely disappeared. He no longer resisted during riding and showed increasing relaxation in arena work.

September 2025: Previous hoof management including an invasive trim so far failed to realign the hoof capsule. Prior to a change in trimming strategy, radiographs of the left forelimb (Supplementary Figure 23) were taken. The findings were an almost ground-parallel coffin bone with good sole thickness and moderate navicular bone changes, which cause no clinical symptoms. Subsequently, the hoof trim was adjusted by the horse owner and the underrun heels as well as the height of the toe could be corrected successfully without compromising the toe wall. The coronary band angle on the left front is ~27° now, and ~30° on the other feet.

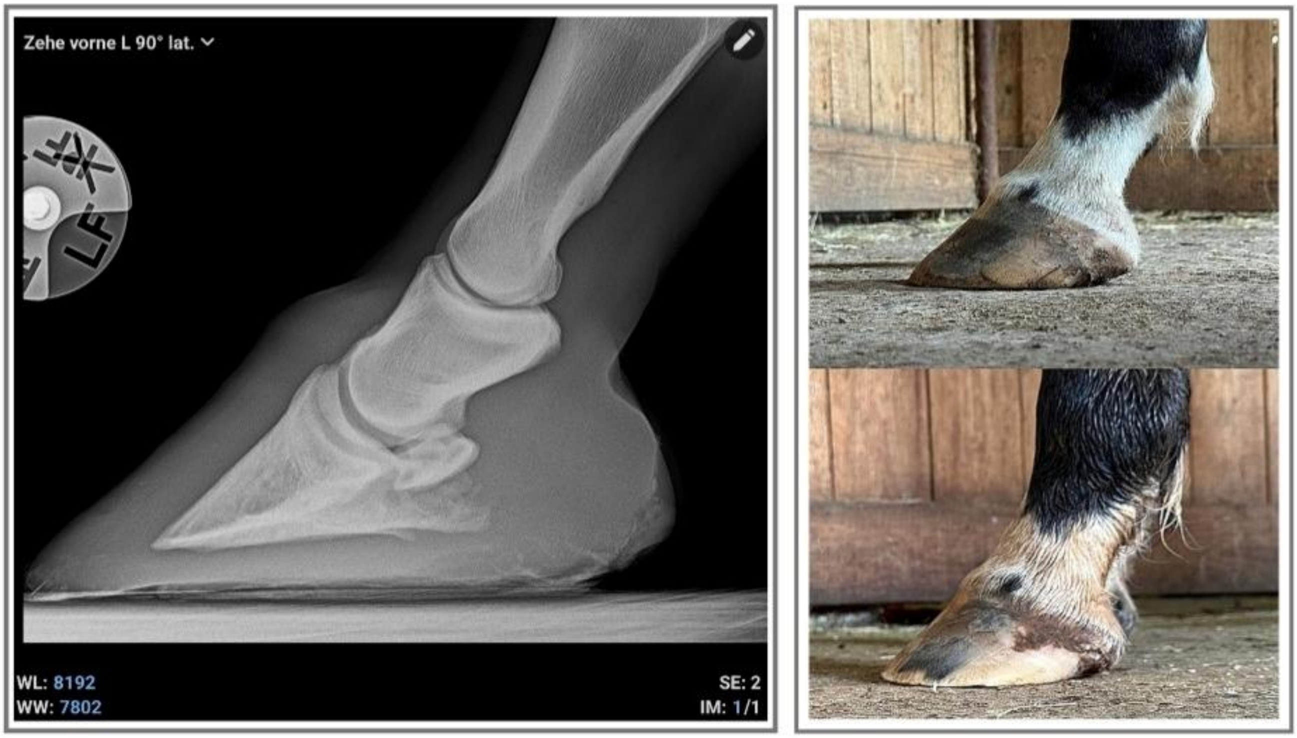

Supplementary Figure 23. *The Radiograph of the left forehoof was taken in September 2025 to check the sole thickness and rule out a negative palmar angle. The upper right photograph shows the left fore in September 2025 with the hoof wall still not in full contact with the ground and with cracks from the invasive trim some months ago. The bottom picture shows the same foot after the last trim by the owner at the end of October 2025.*

3.4.3.2 Case 4 current status

By November 2025, the gelding is overall friendly, social, and easy to handle by anyone. He has not shown any aggressive behavior against humans since the transition to PSF^{+} in 2024. In addition:

- Case 4 now is sensitive yet responsive under saddle.
- The horse is calm and reliable in all situations.
- No therapeutic interventions are needed anymore.

3.4.4 Case 4 conclusion

PSF^{-} represents a substantial risk factor for retiring racehorses, as their bodies are conditioned to levels of training intensity that leisure work rarely provides. In this case, previous behavioral issues and pain responses can retrospectively be linked to former spinous process impingements. The resolution of clinical signs associated with these impingements coincided with a shift in training focus toward restoring structural balance and self-organization, indicating a transition consistent with PSF^{+}. The overall history illustrates that systemic coordination and resilience can re-emerge even when conventional performance or rehabilitation approaches fail to produce lasting improvement.

Equally remarkable was the transformation in behavior: the horse's former aggression toward humans and unpredictable reactions under saddle disappeared once functional postural organization was restored. This rapid behavioral normalization supports the view that many forms of defensive, explosive, or otherwise problematic behavior originate from systemic instability and physical discomfort rather than temperament. By addressing the underlying PSF^{-} mechanisms through the re-establishment of coordinated self-organization, this case highlights the close interdependence of posture, movement, and emotion in equine welfare.

3.5 Case Study 5: Systemic Recovery Limited by Distal Hoof Asymmetry during a PSF$^+$ Transition in a German Riding Pony

Case 5 represents a common subgroup of ridden leisure and junior sport horses that present with unspecific behavioral and locomotor disturbances unresponsive to conventional veterinary or therapeutic intervention.

Data were collected from veterinary diagnostics, radiographic follow-ups, and longitudinal owner documentation between 2021 and 2025. Hoof geometry and lameness development were monitored in parallel.

An integrative approach focusing on systemic self-organization rather than localized correction initiated a progressive PSF$^+$ cycle characterized by improved locomotor symmetry, postural stability, and behavioral normalization. The mare regained rhythm and range of motion, and tactile hypersensitivity resolved. However, persistent mechanical imbalance between the forehooves, with the right forehoof being markedly steeper, led to asymmetric load distribution and subsequent bone edema in the proximal phalanx. Corrective trimming aimed to reduce dorsal compression and re-establish balanced alignment between the hoof capsule and the distal phalanx.

This case demonstrates that systemic functional organization can be restored where conventional treatments fail to identify underlying causes. However, unresolved hoof asymmetry may perpetuate focal overload even within an advancing PSF$^+$ cycle. Sustainable rehabilitation requires the coordinated adaptation of both training and hoof care to maintain long-term biomechanical integrity.

3.5.1 General information on case study 5 subject

Case Identifier: Case 5
Date of birth: 24.04.2013
Breed: German Riding Pony
Sex: Mare

3.5.2 Case 5 PSF$^-$ development

3.5.2.1 Case 5 PSF$^-$ history

June 2021: The German Riding Pony mare was purchased at age 8 by her at that time juvenile owners as a healthy, cooperative horse with no reported medical findings. Retrospective evaluation indicated a distinct high/low hoof conformation at the time of purchase, with both forehooves shod.

During 2021–2022, the mare was ridden by her owners, friendly and without pressure, primarily in a forward and down frame (English Riding Style). Although behavior remained compliant, locomotor performance and range of motion did not improve as expected with proceeding training of the horse. No estrous cycles were observed.

November 2022: The mare developed pronounced tactile hypersensitivity, teeth grinding, and reluctance to move forward. Episodes of stiffness and decreased mobility became evident. Empirical treatment for presumed gastric ulceration with Omeprazole and Sucralfate produced no improvement.

March 2023: The mare was admitted to a referral clinic because of severe movement disturbance and behavioral irritability. Hormonal regulation with Altrenogest was initiated but discontinued in August 2023 without effect on the mare's behavior or performance. A muscle enzyme (MIM) test was negative.

3.5.2.2 Case 5 clinical findings

a. ***General examination, Veterinarian 1 (anonymized; November 2022)***

- Examination due to marked tactile hypersensitivity, teeth grinding, reluctance to move forward, stiffness and reduced mobility
- No clinical findings to explain the issues listed above
- Recommendation: Empirical treatment for suspected gastric ulceration with omeprazole and sucralfate (followed by the owner, no improvement)

b. ***General examination, Veterinarian 1 (anonymized; March 2023)***

- Examination due to severe movement disturbance and behavioral irritability
- Recommendations (followed by the owner):
    - Muscle enzyme (MIM) test (negative)
    - Hormonal regulation with Altrenogest (without effect, discontinued in August 2023)

c. ***Lameness examination, Veterinarian 1 (anonymized; August 2023)***

- Lameness right front
- Radiographs indicate a potential navicular lesion

d. ***Lameness examination, Veterinary Clinic 1 (anonymized; July 2024)****

- Lameness right front recurred
- Radiographic and ultrasonographic imaging shows no abnormalities, navicular lesion ruled out (Supplementary Figure 24, left panel)

e. ***Lameness examination, Veterinary Clinic 1 (anonymized; June 2025)****

- X-rays (Supplementary Figure 1, middle panel) & MRT imaging right forelimb:
    - Bone edema in the proximal phalanx
    - Structural defect of subchondral bone in proximal fetlock bone in sagittal plane with mild sclerosis of surrounding bone
    - Moderate to severe diffuse bone edema-like lesion
    - Overall moderate to severe degeneration of navicular bone with mild bone edema-like lesion in distal region
- Treatment:
    - Local injection of right front fetlock joint with polyacrylamide hydrogel
    - Bisphosphonate injection
- Recommendations (followed by the owner):
    - 6-8 weeks hand walking
    - Supplements for promoting bone and cartilage growth

*f. Follow-up examination, Veterinary Clinic 1 (anonymized; October 2025)**

- Radiographs of front right: complete resolution of bone edema (Supplementary Figure 24, right panel)

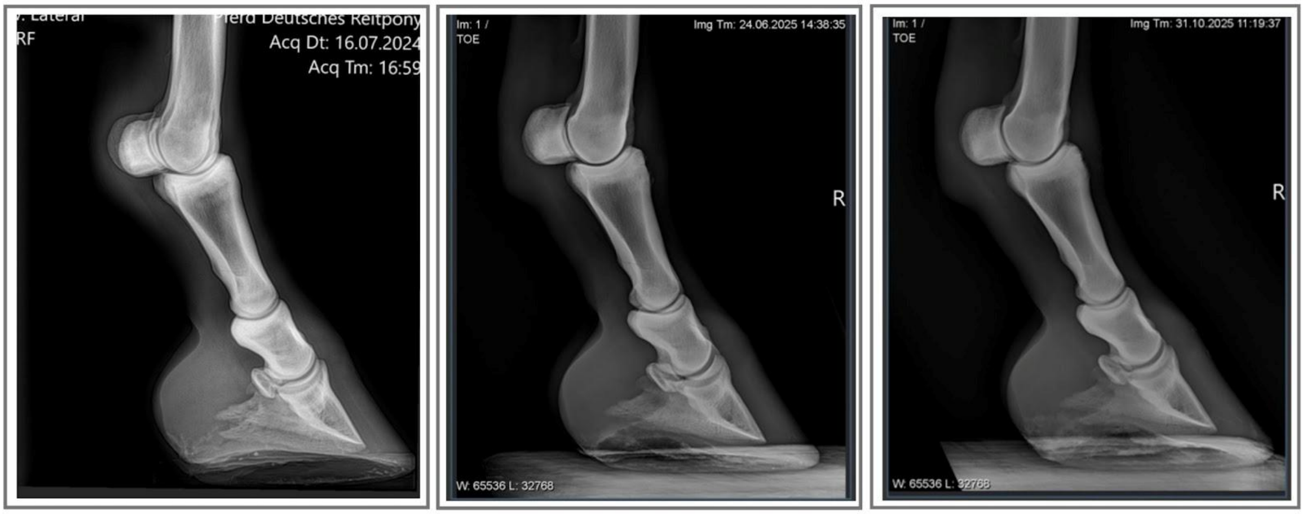

Supplementary Figure 24. *Radiographs of right forelimb of Case 5 from June 2024 (left panel, ~56° dorsal hoof wall angle (DHWA), lameness without findings), July 2025 (middle panel, ~57° DHWA, bone edema) and from October 2025 (right panel, ~53° DHWA, fully recovered and training clearance). The DHWAs were estimated by the authors through digital measurement of the provided X-ray images as displayed.*

3.5.3 Case 5 development after introduction to PSF$^+$

3.5.3.1 Case 5 PSF$^+$ history

July 2023: FIT with horizontal forward tension toward the bit was introduced and replaced the so far applied training regimen. The mare responded rapidly with visibly increased range of motion and greater freedom in her overall movement, accompanied by an improved willingness to work (Supplementary Figure 25). Coordination, rhythm, and postural control improved; tactile hypersensitivity subsided. Despite this systemic improvement, intermittent right forelimb lameness persisted.

August 2023: Radiographs obtained by the attending veterinarian because of lameness in the right front suggested a navicular lesion, which was later ruled out by two independent veterinary clinics (2024 and 2025). The horse was subsequently fitted with egg-bar shoes, producing only transient improvement.

April 2024: The mare was transitioned to barefoot management. A brief improvement in proprioception was noted, but lameness recurred in June 2024 without radiographic or ultrasonographic abnormalities. We interpret here that the horses postural organization improved with the systemic approach, but the hoof capsule geometry (Supplementary Figure 24, left panel) did not yet adapt to these changes, presenting the limiting factor to PSF$^+$ in this case.

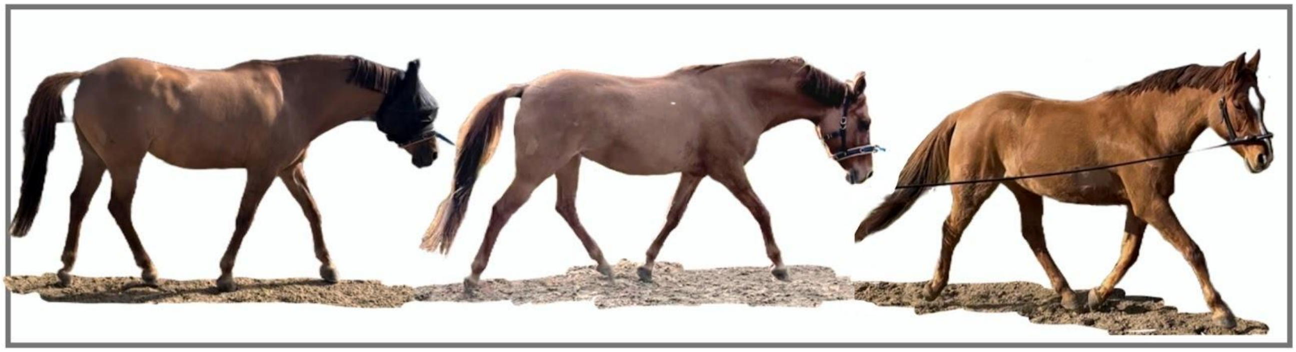

Supplementary Figure 25. *The picture shows the development of the mare from her first painful period in 2022 (left image, February 2022), to 2023 in forward and down (middle image, Summer 2023), and after about 9 months of FIT (right image, Spring 2025).*

June 2025: Clinical re-evaluation identified a bone edema in the proximal phalanx of the right forelimb, for which four months of controlled walking exercise were prescribed and executed. The authors attribute the recurring lameness to the enthusiasm of both horse and riders about the renewed freedom of motion that led to unintentional overuse before full structural adaptation of the distal limb had occurred. The substantial improvement in posture and locomotor function following FIT masked subtle signs of discomfort, leading to a delayed recognition of the developing bone edema. This observation underscores that systemic recovery may temporarily mask localized mechanical limitations. Integrative management must therefore include continuous monitoring of hoof geometry and loading symmetry during functional recovery and corresponding intervention when necessary.

June 2025: The radiographs taken four weeks after the last trim (Supplementary Figure 24, middle panel) showed that rapid heel growth during the trim cycles led to renewed steepening of the hoof.

October 2025: Radiographs—taken a few days after the last trim—showed complete resolution of the bone edema (Supplementary Figure 24, right panel). Comparing the three images in Supplementary Figure 24, in the most recent one a more relaxed lower limb and a lower fetlock- and palmar angle can be seen in comparison to the older images.

During an online consultation involving the owner, the hoof care practitioner, and the first author, the hoof capsule geometry was reviewed and discussed in detail. To ensure an accurate assessment of both hooves, radiographs were obtained to verify the position of P3 within the hoof capsule (Supplementary Figure 26), giving the owner and hoof care practitioner a reliable basis for further decisions.

The author outlined the principles of functional trimming relevant to the case, emphasizing the mechanical implications of the high/low pattern. Persistent steepness of the right forehoof was identified as the likely mechanical cause of overload, and corrective trimming toward a lower hoof angle with continued light heel maintenance was recommended, to preserve the corrected geometry until autonomous growth balance was restored. Following this discussion, all parties agreed to modify the trimming approach as described. A follow-up for this case is planned.

November 2025: The mare has returned to full training without any observable locomotor or postural abnormalities. No signs of discomfort or compensatory movement patterns have been noted during work or daily handling.

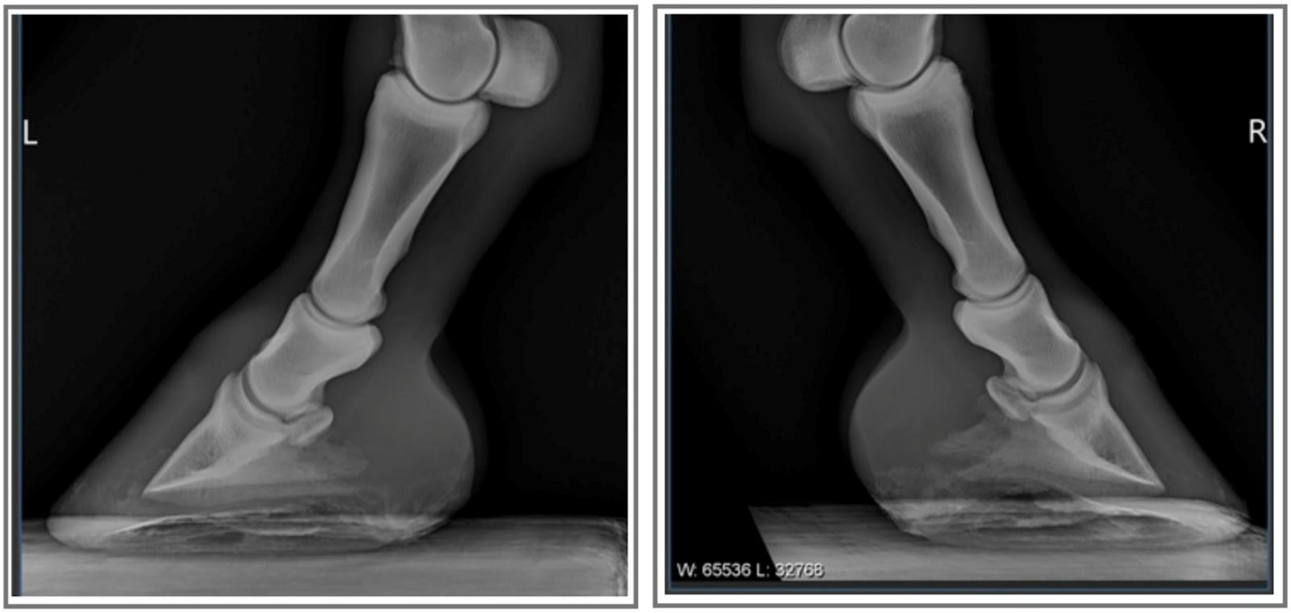

Supplementary Figure 26. *Radiographs of the mare's forefeet acquired in October 2025 reveal still distinct geometric asymmetry between the front hooves. The right forehoof (right panel) is characterized by a steeper dorsal wall, increased heel height, and a more constricted caudal outline, while the left forehoof (left panel) displays a flatter capsule and wider caudal expansion.*

3.5.3.2 Current status Case 5

The introduction of FIT initiated a functional reversion for Case 5 consistent with a PSF^+ trajectory:

- Muscle tone normalized
- Fascial continuity improved
- Coordinated oscillation patterns re-emerged across the thoracolumbar region
- The mare's behavior stabilized
- Movement quality improved substantially (Supplementary Figure 27).

The mare is friendly, reliable and resuming training after the lameness related break was easy; the horse was able to pick up seamlessly where it had been before the break.

3.5.4 Case 5 conclusion

This case illustrates a typical manifestation of PSF^- in a ridden pony and shows that functional reorganization can markedly improve coordination, posture, and emotional balance, even when no clear diagnostic lesion is present. The pony demonstrated stable gains in movement quality (Supplementary Figure 28) and behavior, confirming that system-level training can initiate PSF^+ processes independently of local therapeutic interventions.

At the same time, the case highlights an important limitation: the pony retained a persistent high-low pattern of the forehooves, with a consistently steeper and narrower right hoof. Despite repeated attempts at corrective trimming and shoeing, this asymmetry remained essentially unchanged during the observation period. The renewed lameness—despite ongoing postural and behavioral

improvement—suggests that chronic distal imbalance can act as a structural constraint within the PSF$^+$ process.

Rather than proving that FIT must be combined with any specific form of hoof care, this case demonstrates that the hoof can function as a critical control node: when its geometry remains mechanically divergent, systemic reorganization may not fully translate into sustainable soundness. The pony's persistently positive attitude underscores that behavioral recovery and structural recovery may follow different trajectories when key distal factors remain unresolved.

Overall, this case provides a clear example of how PSF$^+$ processes can begin, stabilize, and still be limited when a major peripheral constraint persists — highlighting the hoof as a potential limiting factor within the system.

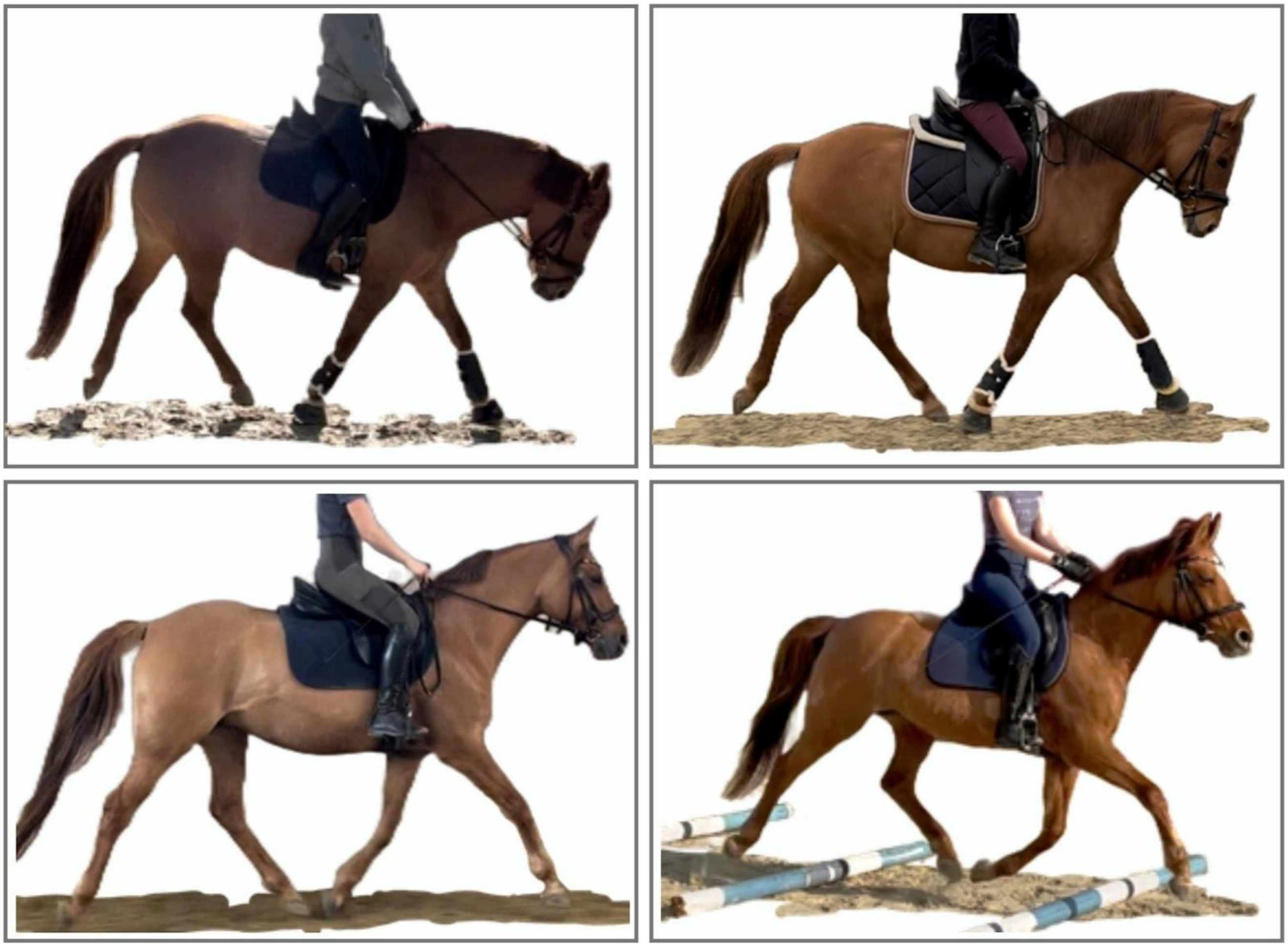

Supplementary Figure 27. *This collection shows the mare's development under the rider. Top row, left panel (02/2021) shows the horse with the croup higher than the withers and in forward-downward posture, and top row, right panel (11/2021) displays an overall improved posture, although the extended stance-phase of the forelimb in trot is still visible. In the bottom row, left panel (06/2024), the phase shift is completely gone, and the poll represents the highest point. In the bottom*

row, right panel (05/2025), Case 5 finally presents with a remarkable increase in range of motion after practicing 9 months in FIT.

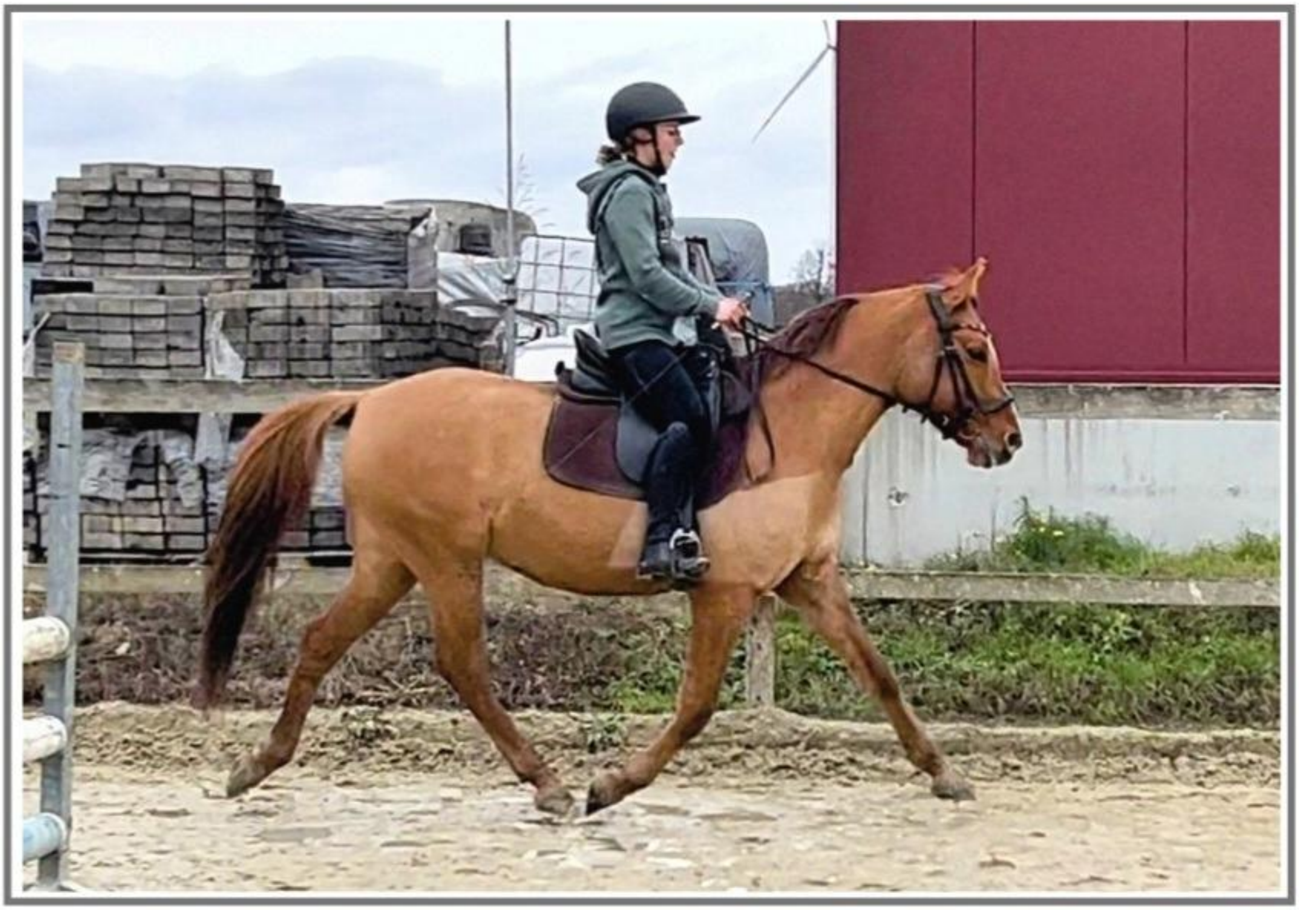

Supplementary Figure 28. *Case 5 at the end of November 2025 in the riding arena after successful rehabilitation.*

3.6 Case Study 6: Systemic and Functional Rehabilitation in a Horse with Cervical Pathologies and REM Sleep Disorder

Case 6 describes a Hanoverian gelding with multiple cervical pathologies, chronic non-specific and shifting lameness, and a pronounced REM sleep disorder, where conventional therapeutic and training approaches provided only temporary or symptomatic relief.

Data was collected from veterinary diagnostics, including radiographic-, CT- and ultrasonic imaging, chiropractic treatment reports, hoof morphology monitoring via photographic documentation and longitudinal owner documentation between 2020 and 2025.

At the time of purchase, the horse presented with severe asymmetric ischial- and pelvic musculature, lameness and brittle hooves. Over the following three years, the horse was neither consistently rideable nor trainable and underwent a variety of interventions including osteopathic treatment, corticosteroid injections, controlled exercise programs, and professional rehabilitation stays, without achieving lasting improvement. Neurological symptoms, chronic pain behavior, and repeated collapse episodes related to REM sleep deficiency persisted.

Comprehensive diagnostic work-up confirmed mild ECVM, osteoarthritis of the lower cervical facet joints, and a bone fragment at C3. Surgical removal of the fragment was performed, whereas a planned decompression surgery at the C6/C7 foramen was later cancelled after clinical reassessment revealed spontaneous resolution of fascial constriction. Rehabilitation subsequently continued with horizontal tension toward the bit and physiologically guided barefoot hoof management emphasizing coordinated movement, proprioceptive feedback, and structural self-organization.

Within a few months, the horse developed marked improvements in postural stability, symmetry, and coordination. Lameness, stumbling, and collapse episodes ceased completely, and the horse's psychological condition stabilized. REM sleep was gradually restored under low-dose pharmacological support and consistent training. The latter contributed to complete resolution of a severe and chronic functional pelvic obliquity. By November 2025, the gelding was fully sound, balanced, and in regular work under saddle, maintaining physiological movement and relaxed behavior.

This case highlights the potential of normalized cervical tension patterns and restored systemic coordination in horses with complex neuromuscular pathologies. The observed structural and behavioral recovery demonstrates that functional reorganization and self-organization within the PSF^{+} framework can replace compensatory patterns and achieve lasting, non-invasive resolution of chronic dysfunction.

3.6.1 General information on case study 6 subject

Case Identifier: Case 6
Date of birth: 17.11.2008
Breed: Hanoverian
Sex: Gelding
Owner: Katharina Bader (Second author of the publication)
Veterinarians: Dr. med. vet. Maria-Dorothee Faust, Veterinarian Michael Oberthür and others (anonymized)

Veterinary Clinics: Tierklinik Lüsche GmbH (Dr. med. vet. Jan-Hein Swagemakers & Dr. med. vet. Christine Fuchs) and other clinics (anonymized)
Hoof-Care Professional: Veterinarian Viviane Xenia Ulrich, hoofnerdyVet Natural Hoofcare

3.6.2 Case 6 PSF⁻ development

3.6.2.1 Case 6 PSF⁻ history

April 2020: The horse was bought by the current owner with PPE indicating lameness in the hind left hock w/o radiographic findings and significant atrophy of the long ischial/pelvic muscles on the left side. The horse was unshod and the hooves in overall brittle condition, the weight was normal (550 kg/1,70m, see Supplementary Figure 29). The horse was very shy and nervous when being handled and worked. Case 6 showed avoidant behavior towards the bit accompanied by hectic and constant chewing on the bit. The selling contract stated that the horse had experienced traumatic events and was difficult when being handled, ridden, in interaction with other horses and in new and unknown situations.

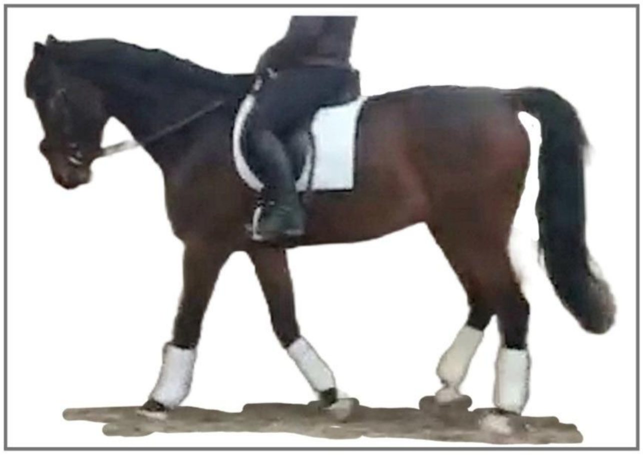

Supplementary Figure 29. *A screenshot from a video at the time of purchase in 2020, the horse in walk, behind the vertical and with visible phase-shift.*

Spring 2020–Summer 2020: Case 6 was unrideable most of the time due to several phases of mixed, shifting lameness on all four limbs and was stumbling and falling during training. The horse had difficulties maintaining the hand canter and often fell into the cross canter. Riding and lunging were practiced according to FN English Riding by the owner, but the training was limited to hand walking most of the time. When bridled with a bit, Case 6 continued his avoidant behavior, and answered any contact (even the weight of the rein) with a behind-the-vertical posture. The handling was difficult, Case 6 spooked in everyday situations, showed severe ground shyness and needed sedation for the farrier; he was shod on the front hooves and kept in hoof boots 24/7 on the hind hooves to prevent abrasion due to pronounced toe dragging. Osteopathic treatment was performed every 6 weeks due to marked functional and postural asymmetries and recurring findings in cervical-, thoracic- and lumbar spine, SI-joint and hips, but had no significant or visible effect on the horse's condition.

Summer 2020–Spring 2022: Training with slow, posture-orientated groundwork and occasional riding under professional instruction, all bitless, was focused but had no positive effect. Lameness and neurological deficits persisted; chronic pain, weight loss, anxiety, and repeated skin lesions at the limbs

were observed. The psychological condition further deteriorated including panic attacks when tied, running away from the owner and general refusal to work. After extensive R+-training, farrier appointments without sedation became possible. Osteopathic treatment was continued by another professional every 3 months without significant effect.

May 2022: A stress colic resulted in severe weight loss (to approx. 500 kg) and was treated with Omeprazole with minor improvement. A REM sleep disorder was diagnosed in a vet-check following the observation of several collapses from standing. Camera observation of the stable showed that the Horse laid down only once briefly in ten days; the stable was subsequently changed from a small group in an open stable to a paddock box.

August–September 2022: A four-week stay in a professional facility with focus on rehabilitation groundwork in forward and down posture (English riding style) and osteopathic treatment resulted in no improvement of Case 6. During the rehabilitation stay, he was shod on all fours to prevent abrasion. Subsequently, an extensive lameness exam in a veterinary clinic was performed, indicating alternating lameness of all limbs, but without conclusive findings. Scintigraphy was recommended but not performed. Feed was changed empirically to MIM-friendly ration with no visible effect on the horse's condition (Supplementary Figure 30, left panel).

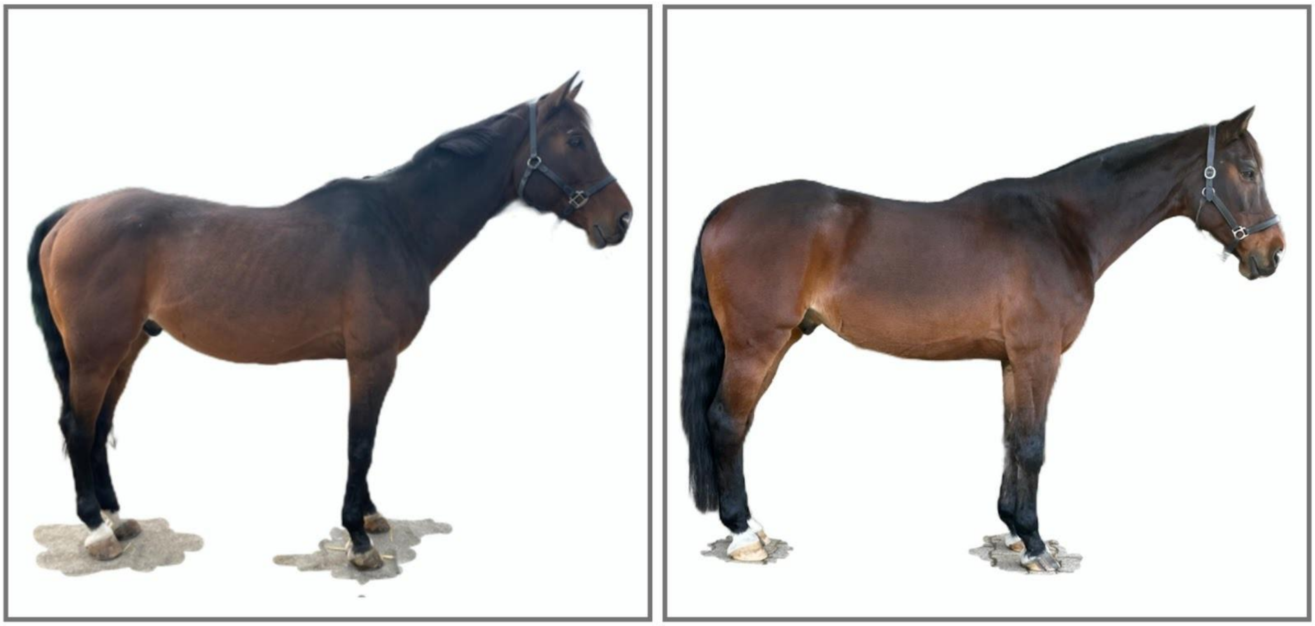

Supplementary Figure 30. *Case 6 in PSF*$^{-}$ *(left panel, picture taken in fall 2022) and PSF*$^{+}$ *(right panel, picture taken in fall 2025). Note the high muscle tone on the left panel and the overall emaciated appearance of Case 6. The right panel visualizes a healthy body condition score and normalized muscle tone after functional reversion through FIT (see below in PSF*$^{+}$ *history).*

October 2022–February 2023: Case 6 was rehomed twice to provide him with a stress-free stabling situation to enable a healthy REM-sleep pattern. Still no REM sleep was observed, and daily collapse episodes with frequent lesions of the skin on legs and head persisted. Furthermore, persistent mixed lameness, pain, stumbling, falling and other neurological deficits were still present. Proprioceptive rehabilitation exercises in walk were performed daily. The consultation of a veterinarian chiropractor

in January 2023 and continuous treatment by this professional from then on revealed severe functional pelvic obliquity and multiple findings over the entire body.

March 2023: Dr. med. vet. Maria-Dorothee Faust diagnosed Case 6 with ECVM (Supplementary Figure 31) and cervical osteoarthritis and applied corticosteroid injections with the recommendation to carefully observe and gentle re-train Case 6 in the following weeks.

March 2023–July 2023: After the treatment, Case 6 was re-trained by lunging and hand-walking which restored partial rideability after some weeks. The horse was mostly free of the previously named symptoms, although still showed unwillingness to work, toe dragging, difficulties to hold hand gallop as well as functional and postural asymmetries. The horse was mostly worked with bitless bridles (sidepull, cavesson) since he still showed the initially described avoidant behavior towards a bit when occasionally tested. Case 6's spookyness when being trained improved, although a severe ground-shyness persisted. Still no REM sleep while laying down was observed and collapse episodes with typical lesions of the skin persisted. Chiropractic treatment in April and July showed improvement in the pelvic obliquity (minor findings in April, medium findings in July) and only a few findings in the rest of the body.

August 2023–September 2023: Up to July, training was possible with 3–4 times riding per week (light dressage and hacking with forward and down position), alternating with lunging and hand walking. In early August, a chiropractic treatment revealed again medium severity of pelvic obliquity and findings along the entire spine of Case 6. Later in August, the overall condition of Case 6 deteriorated again, stumbling in work re-appeared and Case 6 became generally anxious and spooky, again. In August, Case 6 refused to trot when being ridden and became resistful (bucking, rearing). Riding was cancelled, but the resistiveness worsened in lunge work too. Corticosteroid injections (C-spine, LSJ) improved the movement in lunge work temporarily.

October 2023–November 2023: Training reduced to therapeutic sessions in walk and hand walking were performed; no significant improvement observed. Chiropractic treatment in October with similar findings as in August 2023.

December 2023: A CT scan of the neck revealed the presence of a bone fragment at C3 (Supplementary Figure 32) and cervical degeneration; a myelography was planned but aborted due to an anaphylactic reaction of case 6 to the contrast medium.

January 2024–March 2024: Still no improvement in REM sleep disorder. Therapeutic training with focus on strong bending, low head position and posture control combined with fascia release was initiated under professional instruction without positive effect on Case 6's condition.

3.6.2.2 Case 6 clinical findings

a. PPE findings, Veterinarian Michael Oberhür (April 2020)*

- Questionable hoof quality, broken edges
- Mild, mixed lameness in the hind left
- Significant atrophy of the long ischial/pelvic muscles on the left side
- Diagnostic conduction block anesthesia on the hind left was positive for the hock, but X-ray without significant findings

b. *Stress colic diagnosis, Veterinarian 1 (anonymized; May 2022)*

- BCS 3–4
- Suspected stomach ulcers
- REM sleep disorder discovered
- Recommendations (followed by the owner):
    - change from open stable to paddock box
    - Treatment with Omeprazole 2 weeks full dose, 2 weeks half dose

c. *Lameness examination, Veterinary Clinic 1 (anonymized; September 2022)**

- Significantly underweight, overall atrophied & asymmetrical musculature (left hind leg < right)
- Slight joint effusion on hind fetlocks on both sides
- Alternating lameness in front and hind legs, general pain signs; flexion test on all four limbs performed – all negative except on hind left; conduction anesthesia on both hind legs negative
- Imaging diagnostics: Minor age-related changes in knees and hocks, minor reactive findings C6/C7
- Assessment/conclusion:
    - No clear cause of lameness found
    - Imaging diagnostics findings probably age-related or secondary
- Recommendations (not followed by the owner):
    - Whole-body scintigraphy
    - Targeted follow-up diagnostics
    - MIM-diagnostics

d. *ECVM diagnosis, Dr. med. vet. Maria-Dorothee Faust (March 2023)**

- Neurological examination, gait analysis and X-rays
- Findings:
    - ECVM with mild severity (Supplementary Figure 31): caudal aspect of right lamina ventralis of C6 missing, left intact; no transposition at C7; ribs intact
    - Calcifications of cranial nuchal bursa; without findings on clinical examination
    - Osteoarthritis at facet joints of the caudal cervical vertebrae
    - SI-joints reactive in clinical examination
- Treatment:
    - Ultrasound-guided cortisone injections to facet joints of the caudal cervical vertebrae and SI-joints
- Recommendations (followed by the owner):
    - Gentle re-training and observation of symptoms

e. *Follow-up treatments, Veterinarian Michael Oberhür (August 2023)*

- Mixed, shifting lameness on left hind and right front
- Treatment: ultrasound-guided cortisone injection into the facet joints C5/C6 and C6/C7, SI-joints and left hock in multiple sessions

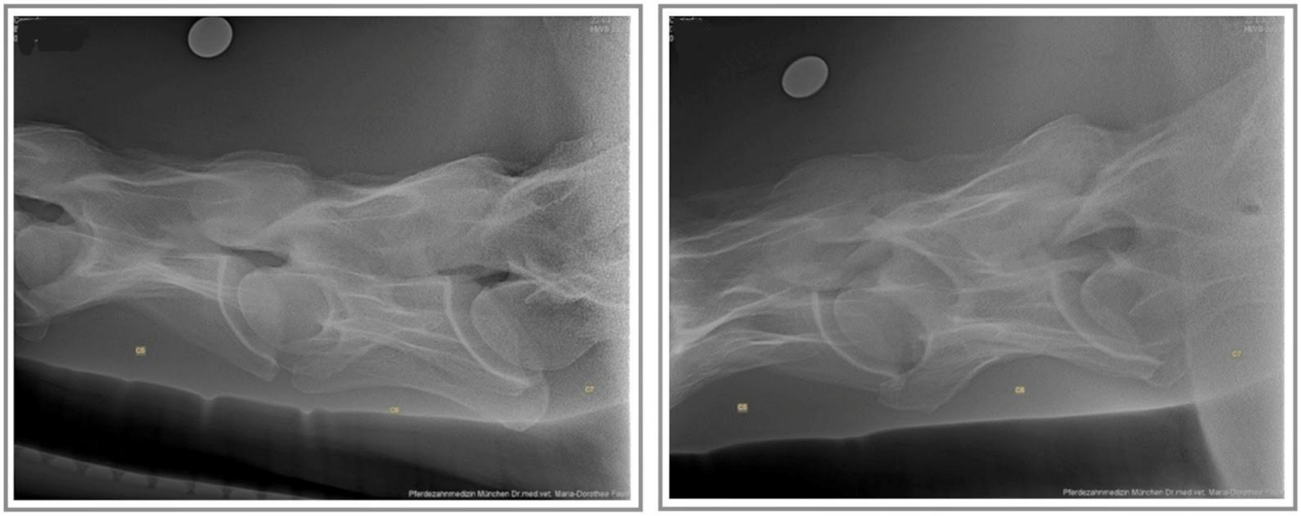

Supplementary Figure 31. *ECVM diagnostics of Case 6 by Dr. med. vet. Maria-Dorothee Faust. Left panel: Latero-lateral view showing intact appearing sixth caudal vertebrae. right picture: LVRDO 20° view showing the caudal aspect of the right lamina ventralis of C6 is missing.*

f. CT scan of the neck in Veterinary Clinic 2 (anonymized; December 2023)*

- A CT scan of the cervical spine was performed under general anesthesia, myelography was planned but aborted due to an anaphylactic shock reaction of Case 6 towards the contrast medium
- Findings:
  - C3/C4: mild to moderate chronic osteoarthritis on the right facet joint with isolated bone fragment at C3 (see Supplementary Figure 4); probably caused by osteochondrosis dissecans with potential differential diagnosis of traumatic avulsion
  - C6/C7: suspected mild cervical malformation (caudal aspect of right lamina ventralis of C6 missing)
  - C6/C7 left: mild chronic osteoarthritis
  - Overall assessment: Degenerative and structural changes in the cervical spine with possible spinal cord compression in the C3/C4- and C6/C7 region
- Recommendation:
  - Clinical correlation of imaging findings (neck mobility, neurological signs)
  - If necessary, further diagnostics or scintigraphy to clarify the cause of pain and functional relevance
  - No surgery recommended

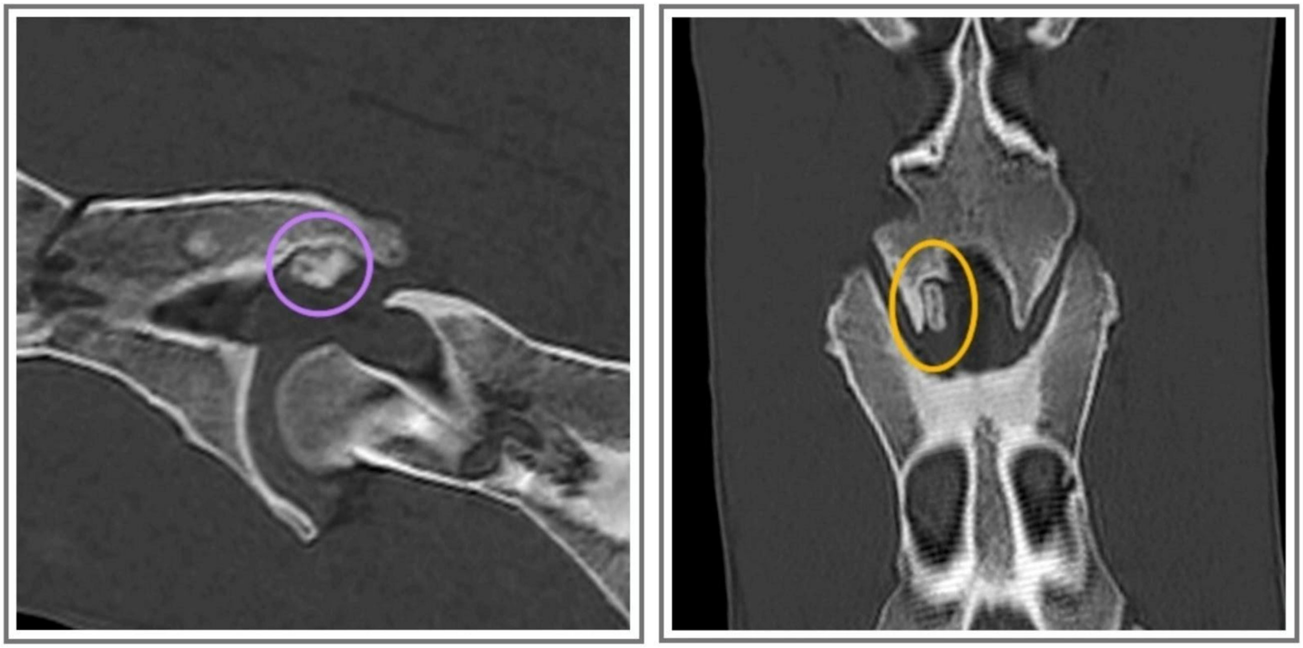

Supplementary Figure 32. *Left panel: Sagittal section of the mid-cervical spine, isolated bone fragment at the ventral margin of the right processus articularis caudalis of C3 marked with purple circle. Right panel: Dorsal section of the mid-cervical spine: The same bone fragment as in the left panel from a different view, marked with yellow oval.*

g. Secondary opinion on CT findings, Tierklinik Lüsche (March 2024)

- In addition to findings of Veterinary Clinic 2, the connective tissue at left foramen at C6/C7 appears hyperdense (suspected nerve-compression)
- Recommendation (followed by the owner):
    - Further clinical investigation: Diagnostic ultrasound-guided injection of cortisone to suspicious areas at C3/C4 and C6/C7 for decision pro/contra surgery

h. Lameness diagnostics and neck surgery, Tierklinik Lüsche (August–September 2024)*

- Neck Surgery & Rehabilitation:
    - Minimally invasive removal of the bone fragment at C3
    - Planned surgical removement of connective tissue at left foramen C6/C7 cancelled (no clinical indication)
- Recommendations (followed by the owner):
    - Comprehensive rehab plan (incl. physiotherapy, controlled lunging, sleep monitoring)
    - Alprazolam treatment for REM sleep facilitation

3.6.3 Case 6 development after introduction to PSF^{+}

3.6.3.1 Case 6 PSF^{+} history

April 2024: Participation of the owner in an online course held by Maren Diehl and Saskia Brieger. The rehabilitation strategy shifted fundamentally with the introduction of FIT with horizontal forward tension to the bit. A simple bar bit was used and within two weeks the horse learned the desired self-

initiated forward tension towards the bit and the previously observed hectic chewing and avoidance of the bit which persisted over many years (also according to the previous owner's statement) completely resolved. The horse did not show any more lameness, stumbling, or falling since the introduction of FIT. Toe dragging and problems holding the hand-canter were still present. The overall state including psychological constitution rapidly improved, although no changes in sleep behavior were observed.

May 2024: After six weeks of gentle re-training with FIT in hand, riding was introduced and duration slowly increased. Case 6 was motivated and willing to work, showing no more of the resistive behavior encountered in PSF^{-} phase.

August–September 2024: The horse had a training pause due to a scheduled surgery (removal of a bone fragment at C3) at the veterinary clinic Lüsche. Clinical re-evaluation by cervical specialists at veterinary clinic Lüsche revealed that the planned surgical correction of connective-tissue constriction at the C6/C7 foramen was no longer indicated and was therefore cancelled. This spontaneous resolution was attributed by us to systemic reorganization and restructuring induced by FIT, which likely reduced fascial tension and restored balanced load distribution in the cervical region. During the almost five-week clinical stay of Case 6, medication was introduced to assist REM sleep. During the clinical stay, no REM sleep and insufficient rest periods in laying position were observed. After one week at home, Case 6 started to show REM sleep and frequent and extensive laying periods (3–5 times per night, 20–90 minutes).

October–December 2024: Rehabilitation according to FIT proceeded with groundwork (lunging, hand walking, free jumping). The REM sleep behavior stabilized and medication weaning-out was started. To facilitate PFS^{+}, hoof management (Supplementary Figure 33) was transitioned to barefoot trimming done by the owner, including adjustments guided by the horse's natural loading patterns and proprioceptive response. Chiropractic treatment in November revealed skeletal symmetry and improved hoof condition, although a medium pelvic obliquity was still diagnosed among other findings over the entire body. This was attributed from our side to the previously performed surgery and accompanying circumstances (surgery under full anesthesia including handling of the horse via crane, several hours transport to and from the clinic etc.). Case 6 normalized in Winter 2024 to almost completely physiological movement: no more lameness, no more stumbling, falling, toe dragging. The ability to hold hand canter improved with the procession of the training.

January 2025–March 2025: Due to an injury of the owner, Case 6 continued structured FIT sessions alternating outdoor hand-walking, lunging and free-jumping. He remained barefoot and consistently sound, displaying a calm demeanor, symmetrical movement, and stable posture.

March 2025–October 2025: After the owner's rehabilitation, gentle re-introduction of riding started with increasing duration under the saddle. In March 2025, a chiropractic treatment revealed good muscle tone, although a slight functional pelvic obliquity was still present in addition to some findings along the entire body, and limbs were displaced towards the body center in standing position. The positioning of the limbs in standing position is attributed by us to the not fully functional hoof morphology at this point in Case 6's PSF^{+} journey.

In summer and fall 2025, the regular training plan included two longer rides (10–12km hacking) and one shorter ride (show jumping up to 1 meter, see Supplementary Figure 34) per week, without any recurrence of lameness or neurological issues. Case 6 was eager and willing to work the entire time. The gait was completely physiological by then, no more toe dragging, stumbling, lameness occurred,

and also holding hand-canter was no problem anymore. From a psychological perspective, moderate ground-shyness and being sceptical and careful in unknown situations remained with a tendency to improve with the procession of FIT.

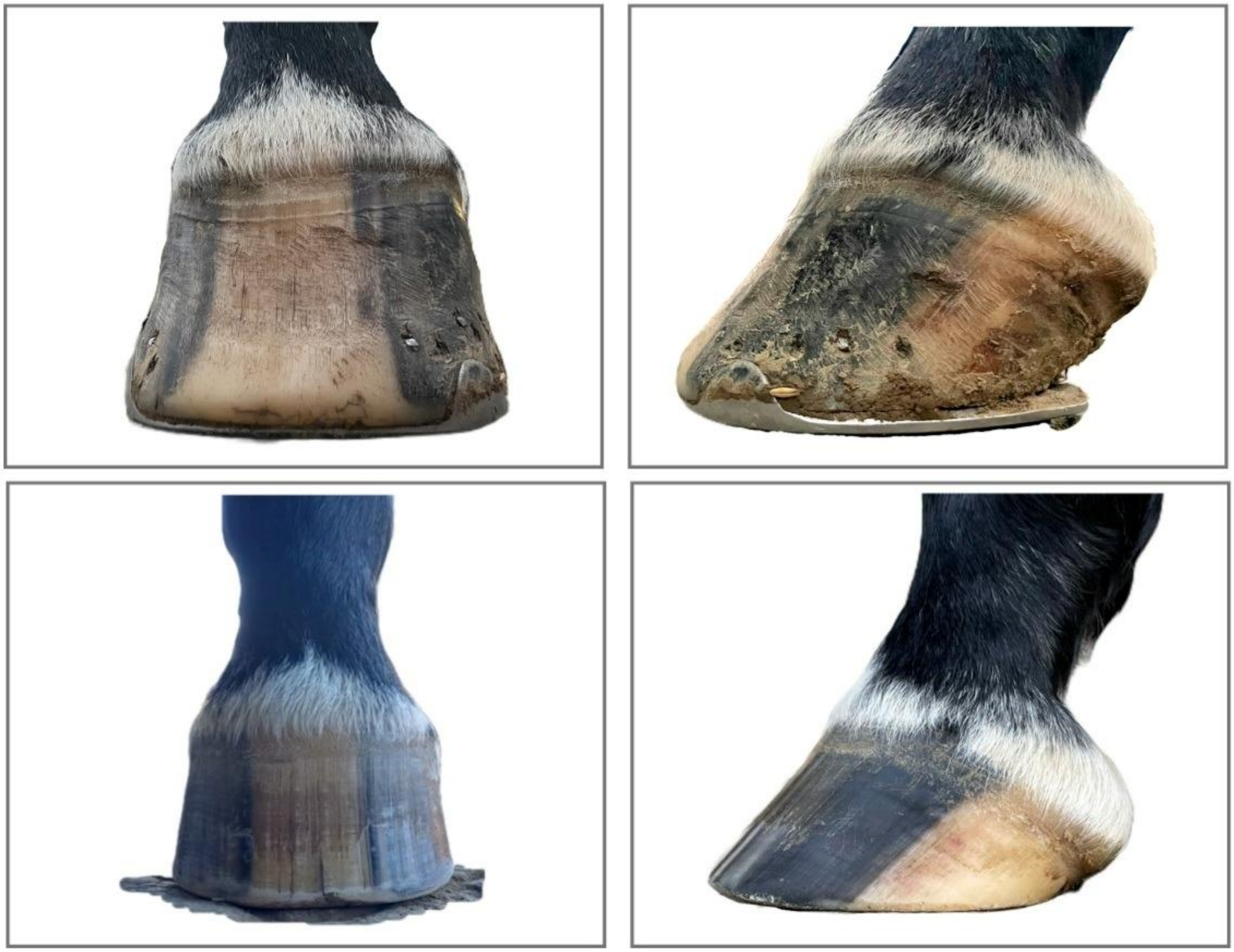

Supplementary Figure 33. *Comparisons of the left front hoof of Case 6. Top row: Dorsal (left panel) and lateral (right panel) view of the left front hoof of Case 6 in October 2024, shortly before the transition to barefoot management. Bottom row: Dorsal (left panel) and lateral (right panel) view of the left front hoof of Case 6 in November 2025, after one year of hoof trimming by the owner and immediately after the first trim of Veterinarian Viviane Xenia Ulrich, hoofnerdyVet Natural Hoofcare.*

October–November 2025: Case 6 developed a smooth topline (see Supplementary Figure 35). Since removal of the horseshoes in October 2024, hoof-care was done solely by the owner, but since the posture in standing position remained to be noticeable with limbs displaced slightly towards the body center, which we attributed to the still not fully physiological morphology of the hoof capsules, the owner initiated professional hoof-care (Viviane Xenia Ulrich, hoofnerdyVet Natural Hoofcare) from October 2025 onwards. With improved balance of all hooves (Supplementary Figure 33, bottom row), the stance position normalized. A subsequent chiropractic treatment in November revealed a good mobility in shoulders, thoracic- and lumbar spine, and was without significant chronic findings.

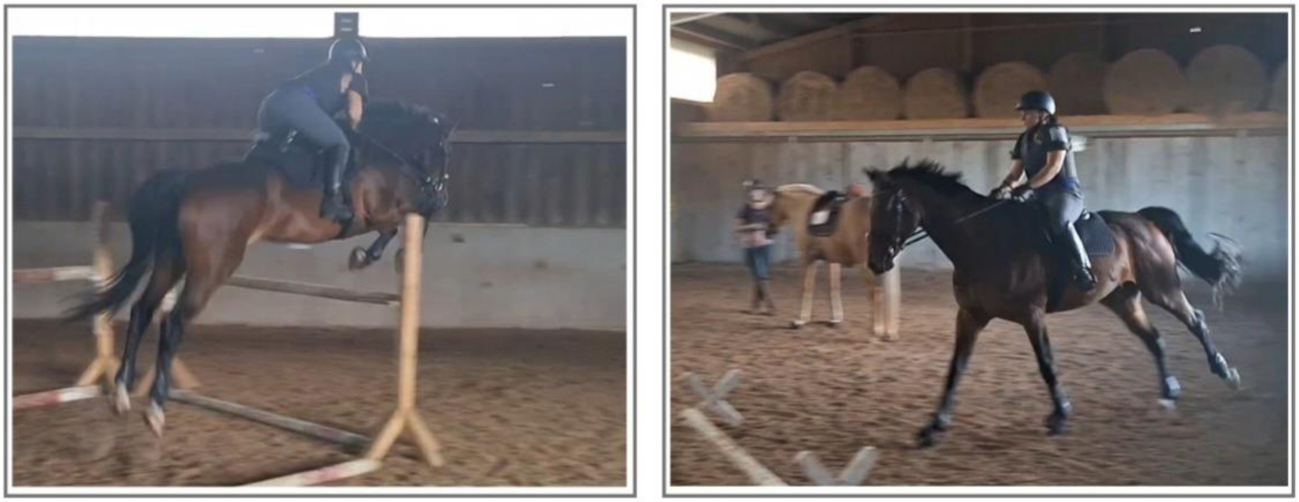

Supplementary Figure 34. *Case 6 in a show jumping training session in September 2025. The horse jumps up to 1m under saddle (left panel) and carries himself according to PSF^{+} (right panel) with active horizontal tension toward the bit, although the available riding arena is very small compared to the horse's frame (horse size 1,70m at withers, riding arena 15x35m).*

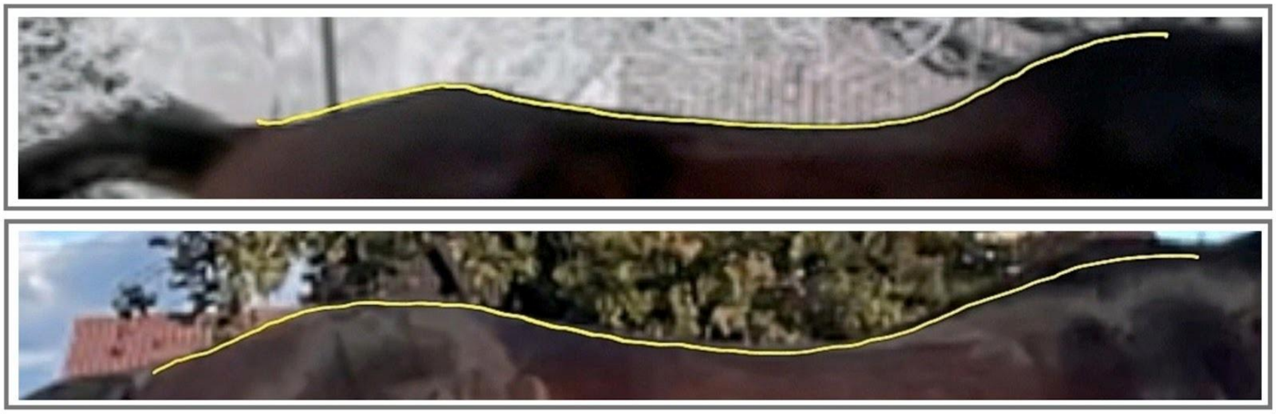

Supplementary Figure 35. *Development of the topline (enhanced by the yellow line for better visibility) of Case 6 in the middle of the stance-phase in trot. Top: Case 6 in PSF^{-} (February 2024), the topline appears atrophied and the edgy bump between the tuber sacrale and lumbar spine indicates an open LSJ. Bottom: Case 6 in PSF^{+} (October 2025), the topline is smooth and the harmonic transition between tuber sacrale and lumbar spine indicates a closed lumbosacral joint.*

3.6.3.2 Current status Case 6

The gelding is sound, relaxed, and mentally balanced. REM sleep is fully restored under low dose of medication, which is gradually withdrawn, with no recurrence of collapses. The barefoot rehabilitation process is still ongoing, with hoof management carried out by the owner with professional support. As a result, the hooves now show even wear, and the horse demonstrates remarkable improvements in confidence across all training environments. Case 6 is highly motivated to work, and presents himself with positive, self-initiated forward tension towards the bit and clear, phase-synchronized gaits

(Supplementary Figure 36). The combination of FIT and hoof management continues to support coordinated movement, resilience, and long-term structural health.

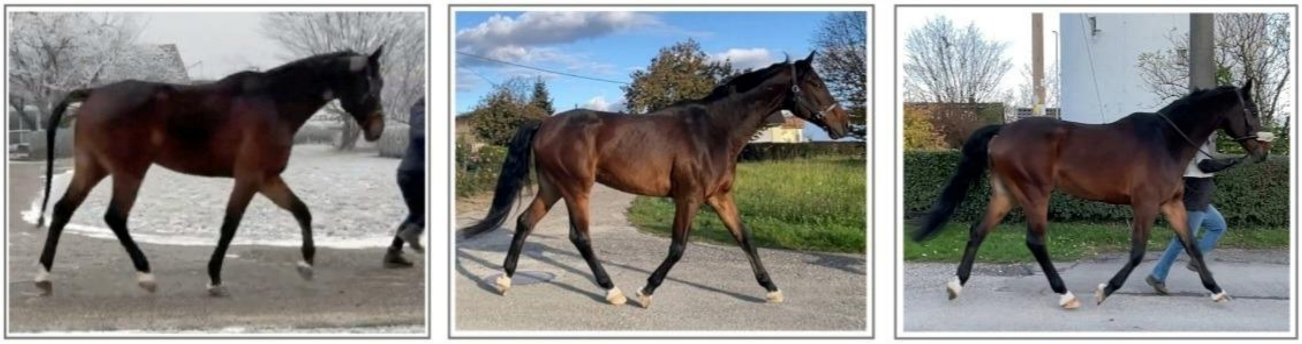

Supplementary Figure 36. *Development of Case 6 in hand at the beginning of the suspension phase in trot. Left panel: Case 6 in PSF⁻ before the initiation of FIT (January 2024). Note the prolonged stance phase of the front right and overall atrophied body condition. Middle panel: Case 6 in PSF^{+}, phase synchronized, although the horse is being pulled forward on the halter (October 2025). Right panel: Case 6 in PSF^{+}. With active horizontal forward tension, the horse's suspension phase becomes more pronounced, even with a bitless bridle (October 2025).*

3.6.4 Case 6 conclusion

The Hanoverian gelding had a long and complex history of systemic dysfunction, chronic pain, and behavioral instability, including anxiety, ground shyness, and episodes of collapse associated with REM sleep deficiency. Between 2020 and 2023, various therapeutic and training approaches produced only temporary or partial relief without restoring stable coordination or confidence.

Following the introduction of FIT in early 2024—several months before the surgical removal of the C3 bone fragment—significant improvement was observed in posture, coordination, and emotional balance. This early progress suggested that systemic reorganization was already underway prior to surgery and that the subsequent rehabilitation phase could build on a substantially stabilized foundation. Continued training following FIT principles, combined with physiologically guided hoof management, led to complete restoration of movement quality, postural balance, and behavioral stability. The horse now shows movement with symmetrical coordination, displays calmness in handling and under saddle, and maintains a stable barefoot condition with even wear patterns.

This case demonstrates that functional and behavioral recovery in horses with complex cervical and neuromuscular disorders can be achieved through coordinated self-organization within the PSF^{+} framework. It further illustrates how integrated, non-invasive rehabilitation approaches such as FIT can foster lasting improvements in both structure and behavior, complementing conventional veterinary treatment pathways. At the same time, it indicates limitations of PSF^{+}; both fragment surgery and medication to stabilize REM sleep were prerequisites for Case 6 to holistically activate his biotensegral potential and fully recover.

Finally, this case study highlights a broader principle: many established or commercially promoted training and therapy systems focus on local correction, postural alignment, or muscle activation but fail to address systemic coordination. Within the PSF framework, this limitation explains why previous

interventions, although well intended and professionally applied, did not result in lasting improvement. Sustainable change emerged only once functional self-organization was re-established.

3.7 Case Study 7: Physiological Hoof Management and Changes in Training Methods as a Pathway to Sustainable Recovery from Chronic Lameness

Case 7 represents horses with chronic lameness and postural instability, which are primarily caused by mechanical hoof compression and asymmetric loading.

Data were collected from veterinary diagnostics, radiographic follow-ups, and longitudinal owner documentation between 2023 and 2025. Hoof geometry and lameness development were monitored in parallel.

The Baden-Württemberger gelding in this case developed recurrent forelimb lameness and cervical tension in succession to an accident. Corrective shoeing and conventional rehabilitation provided only temporary relief. After transitioning to barefoot management and training focused on balanced load distribution and dynamic postural control, the horse rapidly regained coordination, stability, and confidence under saddle.

Lameness and stumbling resolved completely, respiratory issues subsided, and the horse remained sound without further therapeutic intervention.

This case demonstrates how the focus on self-organization in everyday training combined with functional hoof rehabilitation can restore systemic balance and sustainable soundness in horses with chronic lameness related to compressive hoof geometries.

3.7.1 General information on case study 7 subject

Case Identifier: Case 7
Breed: Baden-Württemberger
Sex: Gelding
Date of Birth: 13.05.2013
Hoofcare Professional: Veterinarian Viviane Xenia Ulrich, hoofnerdyVet Natural Hoofcare

3.7.2 Case 7 PSF⁻ development

3.7.2.1 Case 7 PSF⁻ history

February 2020: Case 7 was purchased for the owner’s daughter for Military riding on level B1/B2 (Switzerland).

February 2021: Shoeing transitioned to rocker shoes because the horse lost his front shoes frequently in the pasture.

Summer 2021: The owner started dressage training and endurance riding. Case 7 developed progressive stiffness, reduced functional engagement of the lumbosacral region, increased cold sensitivity, diminished strength, and moments of reduced sure-footedness.

November 2023: Tested positive on MIM n/P2, which led to a recalculation of food rations and changes in management (blanketing).

May–June 2024: A stable accident in which the gelding struck the chest and forelimbs against a tie post resided in recurrent right forelimb lameness without clear findings in veterinary examination. Radiographs were initially interpreted as normal, and the horse was treated with Phenylbutazone for 10 days. After treatment, the lameness worsened.

July 2024: A second veterinary opinion noted marked reluctance to move and possible cervical or gastric involvement. Early cervical osteoarthritis and left forehoof irregularities with mild ossification of the lateral cartilage were found. The horse frequently coughed when trotting and required to be fed with steamed hay or haylage. Radiographs excluded ECVM but ultrasonography revealed mild cervical arthrosis and increased synovial fluid. Intra-articular medication of the cervical joints was performed. Six weeks of controlled exercise at a brisk walk were recommended and followed.

August–September 2024: Rehabilitation exercises, introduced by the veterinarian with focus on deep, low, bended posture and sidewards movements were performed. These induced stress and behavioral discomfort in the horse, resulting in repeated tripping and falls and therefore were cancelled after some weeks. Despite supportive exercises and hill work, recurrent left forelimb lameness persisted with more than one minute of trotting.

December 2024: A follow-up examination revealed a highly compressed heel on the left fore hoof (Supplementary Figure 37) and a corrective shoe was applied under radiographic guidance. Initial improvement with the special shoes was only temporary.

3.7.2.2 Case 7 clinical findings

a. ***MIM diagnostics, Veterinary Clinic 1 (anonymized; November 2023)***

- MIM tested positive: n/P2

b. ***Lameness examination after accident, Veterinarian 1 (anonymized; May 2024)***

- Recurrent lameness right front
- X-rays without findings
- Recommendation (followed by the owner):
  - 10-day treatment with Phenylbutazone

c. ***Secondary lameness examination, Veterinarian 2 (anonymized; July 2024)****

- Marked reluctance to move, potential cervical or gastric involvement?
- Radiographic findings:
  - Cervical spine: no ECVM, mild cervical arthrosis
- Ultrasonographic findings:
  - Increased synovial fluid
  - Intra-articular medication of the cervical joints was performed
- Recommendations (followed by the owner):
  - Six weeks of controlled exercise at a brisk walk

d. ***Follow-up examination, Veterinarian 2 (anonymized; August 2024)***

- Introduction of therapeutic training

- Recommended 10 minutes of training per day (followed by the owner for some weeks with negative result)

*e. **Follow-up examination, Veterinarian 2 (anonymized; December 2024)****

- Radiographs of the left forehoof (see Supplementary Figure 37)
- Irregularities with mild ossification of the lateral cartilage
- Recommendations (followed by the owner):
    - o Corrective shoeing

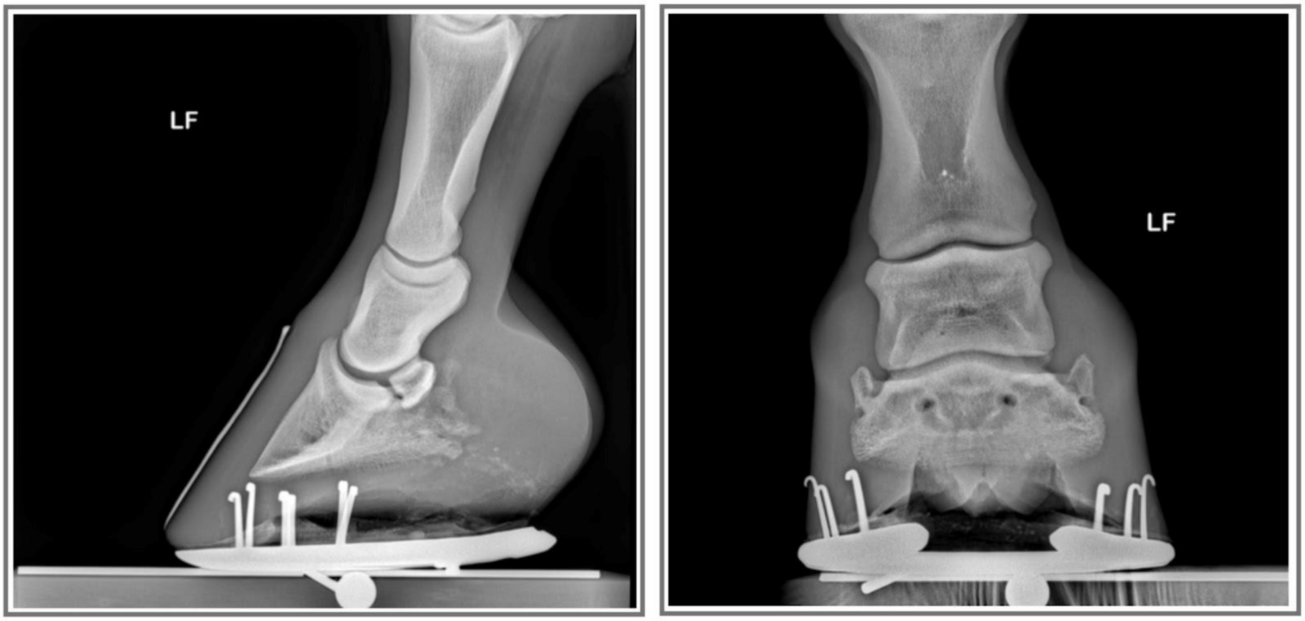

Supplementary Figure 37. *Radiographs of the left front hoof in lateral (left panel) and dorsopalmar (right panel) view of Case 7 with rocker shoes in December 2024 before application of the corrective shoe.*

3.7.3 Case 7 development after introduction to PSF$^+$ concept

3.7.3.1 Case 7 PSF$^+$ history

December 2024: During the preliminary consultation before the first online-course participation of the owner with the first author, Case 7 was presented as an insecure, stumbling horse with recurrent lameness and respiratory issues.

January 2025: In preparation for the course, the decision was made to remove the shoes and transition to barefoot management. The condition of the hooves (Supplementary Figure 38, left panel) indicated that initiating barefoot management prior to the start of the FIT course in January 2025 would be essential to ensure optimal adaptation prior to the start of training. Subsequently, the training was started, emphasizing active horizontal tension towards the bit and functional interaction under load.

January 2025–April 2025: Bare-hoof trimming was provided every six weeks by Veterinarian Viviane Xenia Ulrich, hoofnerdyVet Natural Hoofcare, while the horse was trained under continuous supervision in FIT. The horse's musculoskeletal functions improved; strength, stability, and movement

organization increased significantly. Within months, he was able to carry his rider securely in all gaits over varied and difficult terrain.

May 2025–November 2025: The owner continued training within follow-up courses guided by Maren Diehl. Throughout this period, both the gelding's motor coordination and overall health improved steadily. The interaction between rider and horse became increasingly harmonious, reflecting enhanced mutual perception, timing, and functional stability. Hoof-Management promoted these developments in the sense of PSF^+ (Supplementary Figure 38, right panel).

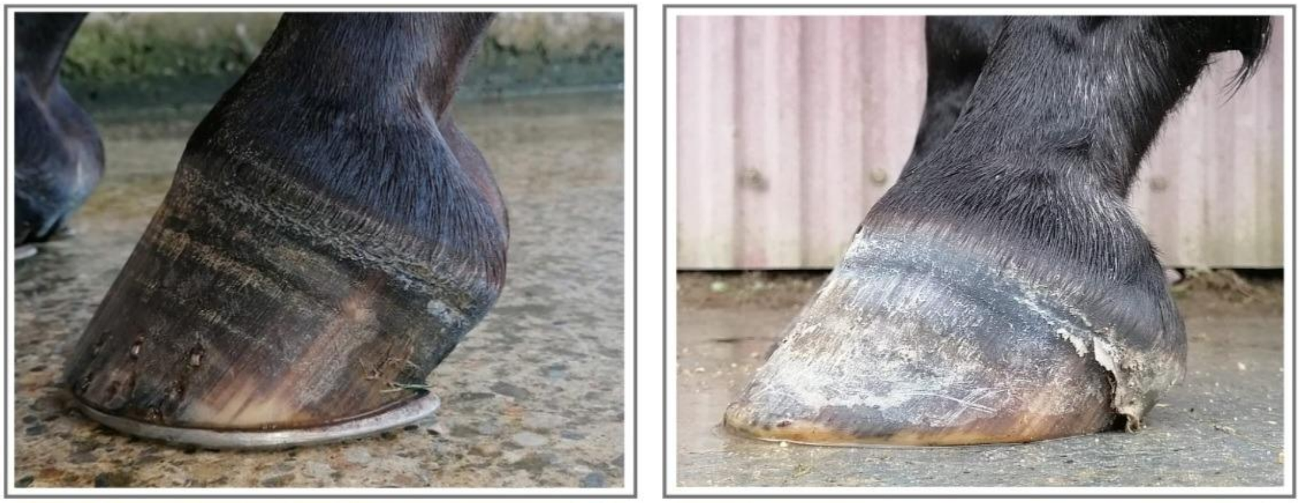

Supplementary Figure 38. *The left front hoof in $PSF^–$ state (left panel, December 2024) and in almost fully recovered PSF^+ state after nine months of barefoot trimming by Veterinarian Viviane Xenia Ulrich, hoofnerdyVet Natural Hoofcare (right panel, September 2025). These photographs represent the raw data underlying Figure 5 in the main text.*

Retrospective review of the case revealed that the application of rocker shoes was followed by the development of signs of the $PSF^–$ cycle in Case 7 (progressive stiffness, reduced functional engagement of the lumbosacral region, increased cold sensitivity, diminished strength, and moments of reduced sure-footedness). These signs prompted not only further veterinary evaluation but also caused the owners ambition "to become a better rider". With the benefit of current knowledge about hoof geometry and the role of stable ground interaction in functional movement, it seems possible to the authors that the rocker shoes contributed to the reported negative functional changes. While no direct causal inference can be made, the temporal context, combined with the later improvement once the rocker shoes were discontinued, suggests that shoeing was a relevant factor in the overall development.

3.7.3.2 Current status Case 7

The horse is currently sound, strong, and stable under saddle:

- He presents overall improved posture and muscle development (Supplementary Figure 39)
- Case 7 moves sure-footed on uneven ground
- The horse shows no signs of respiratory distress - the previous cough resolved, although steamed hay continues as a precaution

- Outdoor rides up to two hours without boots are possible, everyday rides are one-hour-hacks with and without boots about 7.5 kilometers in challenging terrain with trot and some gallop wherever the tracks allow.

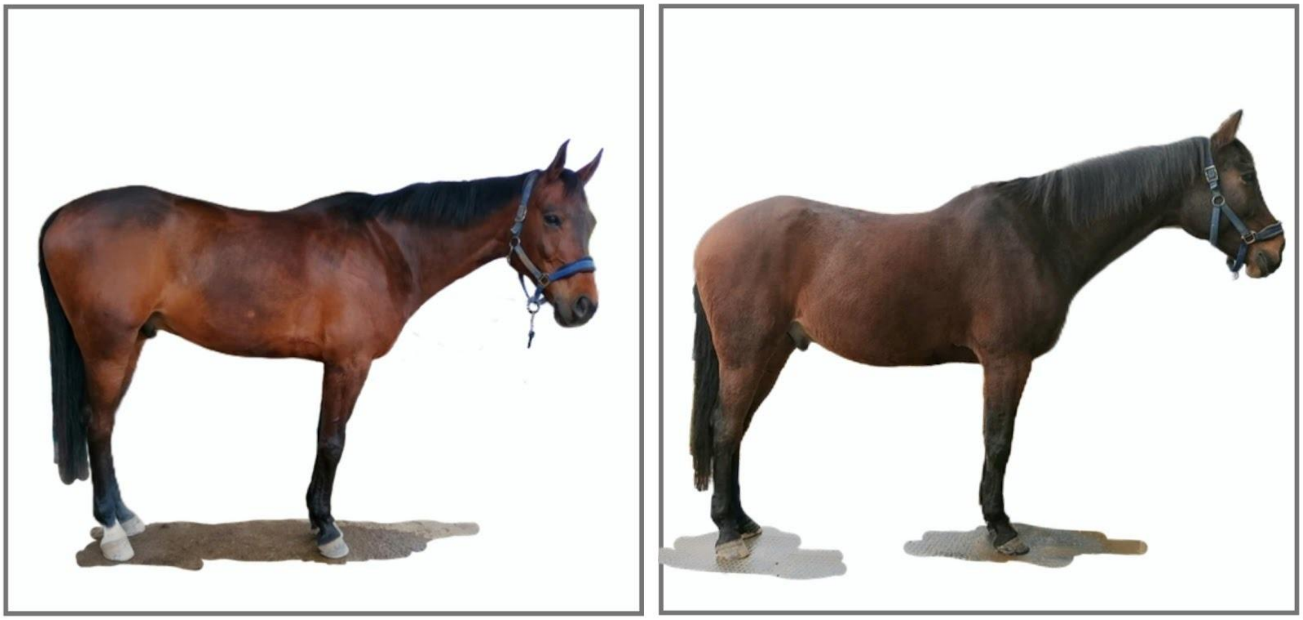

Supplementary Figure 39. *Left Panel: Case 7 in PSF^{-} state (April 2024). The front limbs are markedly displaced caudally, the croup is higher than the withers and the transition between tuber sacrale and lumbar spine indicates a dysfunctional lumbosacral joint. The topline displays a distinct forward-sloping configuration from croup to withers, characteristic of insufficient structural support (image mirrored along vertical axis for better comparability). Right panel: Case 7 in current PSF^{+} state (October 2025). All PSF^{-} signs in posture have improved.*

3.7.4 Case 7 conclusion

This case illustrates the restoration of systemic postural balance and locomotor stability in a gelding with chronic lameness, reduced coordination, and respiratory issues through the combined application of FIT and physiologically guided barefoot rehabilitation. The shift from a compressive, asymmetric hoof geometry toward balanced loading marks a transition from PSF^{-} to PSF^{+}.

Retrospective evaluation showed that the period of rocker-shoe application was followed by functional impairments characteristic of the PSF^{-} cycle. While no causal inference can be drawn, the temporal association—together with the subsequent improvement after discontinuation of the rocker shoes—suggests that the altered hoof–ground interface was a relevant contextual factor in the horse's decline.

Sustained recovery after the failure of previous conventional approaches indicates that lasting improvement resulted not from isolated structural correction but from global neuromuscular reorganization. The training supported functional load redistribution, improved proprioceptive regulation, and re-established self-stabilizing movement patterns. Notably, these outcomes were achieved by an experienced leisure rider under continuous remote guidance, underscoring the potential for systemic improvement even without in-person instruction.

3.8 Case Study 8: PSF$^+$ Development and its Limitations in a High-Level Endurance Horse

Case 8 represents high-level competition horses that, during or following intensive athletic careers at international championship level, develop progressive structural strain and overload-related injuries that may compromise their regenerative capacity.

Data were collected from veterinary diagnostics, radiographic follow-ups, and longitudinal owner documentation between 2020 and 2025. Hoof geometry and lameness development were monitored in parallel.

The Arabian gelding in this case competed successfully in endurances over distances up to 160 km at European Championship level before developing chronic back pain and later a suspensory ligament branch lesion in the forelimb. Following conventional rehabilitation and medical treatment, FIT was introduced during the recovery phase, focusing on structural balance, coordination, and movement efficiency. Marked improvements were observed consequently in rhythm, posture, and muscular stability, enabling a return to mid-level endurance competition without clinical pain or lameness. However, residual vulnerability in the limb structures indicates the biological limit of full regeneration after long-term high-performance strain.

This case highlights both the regenerative potential and the physiological boundaries of the PSF$^+$ approach for instance in elite endurance horses, demonstrating that sustainable function and performance are achievable even when complete restitution might be no longer possible.

3.8.1 General information on case study 8 subject

Case Identifier: Case 8
Date of Birth: 26.04.2009
Breed: Arabian
Sex: Gelding
Hoof-Care Professional: Veterinarian Viviane Xenia Ulrich, hoofnerdyVet Natural Hoofcare

3.8.2 Case 8 PSF$^-$ development

3.8.2.1 Case 8 pre-PSF$^-$ history

The gelding had an extensive international career in endurance racing, including participation in European Championships over distances up to 160 km (Supplementary Figure 40).

- 2013: Started under Saddle
- 2016: In October start at the first Endurance 27 km
- 2018: First international starts
- Following career highlights:
    - 2019 World Championship Juniors 120 km with 18 km/h
    - 2021 Qualifying race 160 km with 18.1 km/h
    - 2021 European Championship Elite 160 km with 20.2 km/h
    - 2023 Qualifying race 160 km with 16.7 km/h
    - 2023 European Championship Elite 160 km with 20.1 km/h

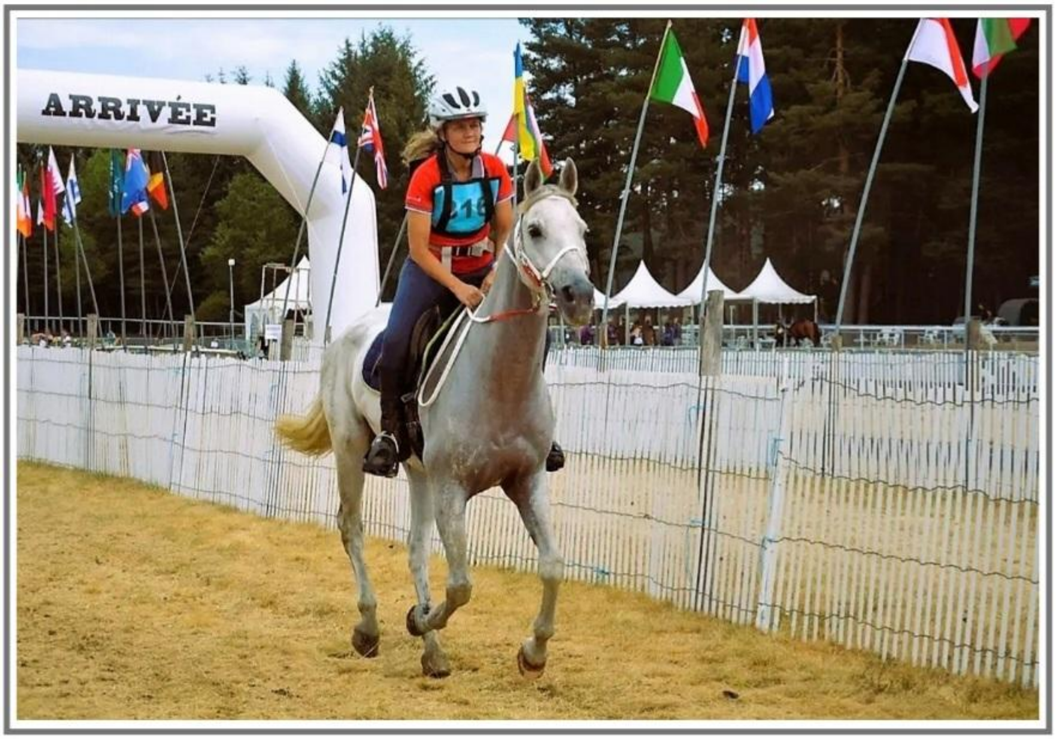

Supplementary Figure 40. *Case 8 at the finish line in the European Championships 2023.*

3.8.2.2 Case 8 PSF^{-} history

After several years at top competitive level, progressive signs of overload developed, including chronic back pain, sacroiliac arthritis, and recurring respiratory strain.

2020–2023: Multiple veterinary evaluations revealed kissing-spine-like lesions in the caudal thoracic region, alongside arthrosis of the lumbosacral and sacroiliac joints.

October 2023: Intermittent lameness associated with a suspensory branch lesion in the right forelimb occurred. Case 8 received standard medical treatment including corticosteroid injections, and systemic anti-inflammatory therapy.

December 2023–February 2024: Stay at a rehabilitation center (due to insufficient manageability at home), including equine treadmill twice daily for 1 hour and aqua trainer once daily for 30 minutes, resulting in training clearance for trot with gradual minute-by-minute increases

February 2024: The owner of Case 8 contacted Maren Diehl prior to online course participation. Case 8 presented at that time with chronic back pain, insufficient trunk stability and overall poor muscle tone. Furthermore, a reduced stride amplitude in the forelimbs and arrhythmic gaits accompanied by reduced coordination was noticeable.

The horse has been continuously shod since 2013, except during his stay in the rehabilitation center (December 2024–February 2024).

3.8.2.3 Case 8 clinical findings

a. Examination of musculoskeletal system & lungs in Veterinary Clinic 1 (anonymized; 2020)

- Clinical Examination:
    - Persistent, unchanging back pain
    - Marked sensitivity to palpation over the thoracolumbar region
    - Unusually high respiratory rate
- Diagnostics via lung endoscopy, radiography, ultrasonography & scintigraphy, findings:
    - Kissing-spines-like lesions in the caudal thoracic spine
    - Arthritis and osteoarthritis of the small articular facets of the cranial lumbar spine
    - Arthropathy of the sacroiliac joints
    - Suspicion of a mild form of Recurrent Airway Obstruction (RAO)
- Treatment:
    - Interspinous injection in the caudal thoracic spine
    - Injections into the small articular facets of the lumbar spine
    - Bilateral sacroiliac joint injections
    - Systemic NSAIDs and Gastrozol
- Recommendation (followed by the owner):
    - Inhalation therapy at home

b. Surgery in Veterinary Clinic 1 (anonymized; August 2022)*

- Surgical removement of a chip fragment in the fetlock joint of the right forelimb

c. Lameness examination in Veterinary Clinic 1 (anonymized; January 2023)

- Clinical Examination:
    - Irregular gait in the hindlimb and recurring signs of back pain
- Diagnosis:
    - Osteoarthritis of the sacroiliac joint, more pronounced on the right side, with increased joint effusion and bone remodeling
    - Muscular back pain
- Treatment:
    - Bilateral sacroiliac joint corticosteroid injections

d. Follow-up treatment in Veterinary Clinic 1 (anonymized; August 2023)

- Prophylactic corticosteroid treatment of the sacroiliac joints prior to the European Championships

e. Lameness examination in Veterinary Clinic 1 (anonymized; data available, October 2023)*

- Diagnosis:
    - Subacute, moderate tendinopathy (strain) of the lateral suspensory ligament branch of the right forelimb
- Treatment:
    - Management according to standard therapeutic protocol

- Recommendations (followed by the owner):
  - Re-evaluation after 4 and 6 months

f. 1st Follow-up examination in Veterinary Clinic 1 (anonymized; February 2024)

- Ultrasonography shows healing progression of the lesion
- Orthopedic examination reveals mild remaining irregularity in the right forelimb
- Recommendation (followed by the owner):
  - Trot work may begin with gradual minute-by-minute increases

g. 2nd Follow-up examination in Veterinary Clinic 1 (anonymized; April 2024)

- Ultrasonography shows progressive healing, with the lesion now completely filled with scar tissue
- Recommendation (followed by the owner):
  - Canter work may begin with gradual increase in duration

3.8.3 Case 8 development after introduction to PSF$^+$

3.8.3.1 Case 8 PSF$^+$ history

February 2024–Summer 2024: The owner contributed in an online course held by Maren Diehl and Saskia Brieger, FIT and especially the horizontal forward tension toward the bit was introduced. The focus was on restoring postural balance, movement coordination, and functional self-organization rather than isolated muscular strengthening. In preparation for mid-level endurance competitions, Case 8 presented with smoother rhythm and coordination in walk and trot, increased trunk stability and muscular tone, and visibly greater stride amplitude in the forelimbs. The back pain had resolved, and the mental state of the gelding was noticeably good.

October 2024: 25 km endurance ride.

April 2025: 50 km endurance ride at an average speed of 14.7 km/h.

May 2025: International 104 km endurance race at an average speed of 15.5 km/h. A post-race ultrasonographic control of the suspensory branch was negative (no abnormalities detected). The gelding was considered fully recovered at this point and presented with superior willingness in work and stable musculature.

Summer 2025: Preparation phase for a 140 km endurance race scheduled for October 2025.

October 2025: Slight gait irregularity following a high-intensity 40 km galloping work, accompanied by marked swelling from the fetlock joint up to mid-cannon of the left forelimb. Subsequent veterinarian examination confirmed early arthritic activity and minor strain to the old scar tissue. According to the attending veterinarians, the horse is expected to remain capable of competing safely in medium-distance endurance rides up to approximately 100 km, while the full 160 km distance would likely exceed the biological tolerance of the compromised tendon structures. The gelding remains fit, well-muscled, and mentally balanced. The recent findings were not preceded by lameness but by a slight decrease in performance capacity.

3.8.3.2 Case 8 clinical findings in PSF^{+}

h. Lameness examination by Veterinarian Clinic 1 (anonymized; October 2025)

- Mild swelling in the previously injured right forelimb after 40 km high intensity galloping work
- Renewed sensitivity over the old suspensory branch lesion
- Early arthritic activity in the affected region
- Limited tolerance of old scar tissue
- Subtle reduction in performance capacity without preceding lameness
- Prognosis:
    - Expected to remain safe for medium-distance endurance competitions (up to ~100 km)
    - Full 160 km endurance distances likely exceed the biological tolerance of the compromised structures

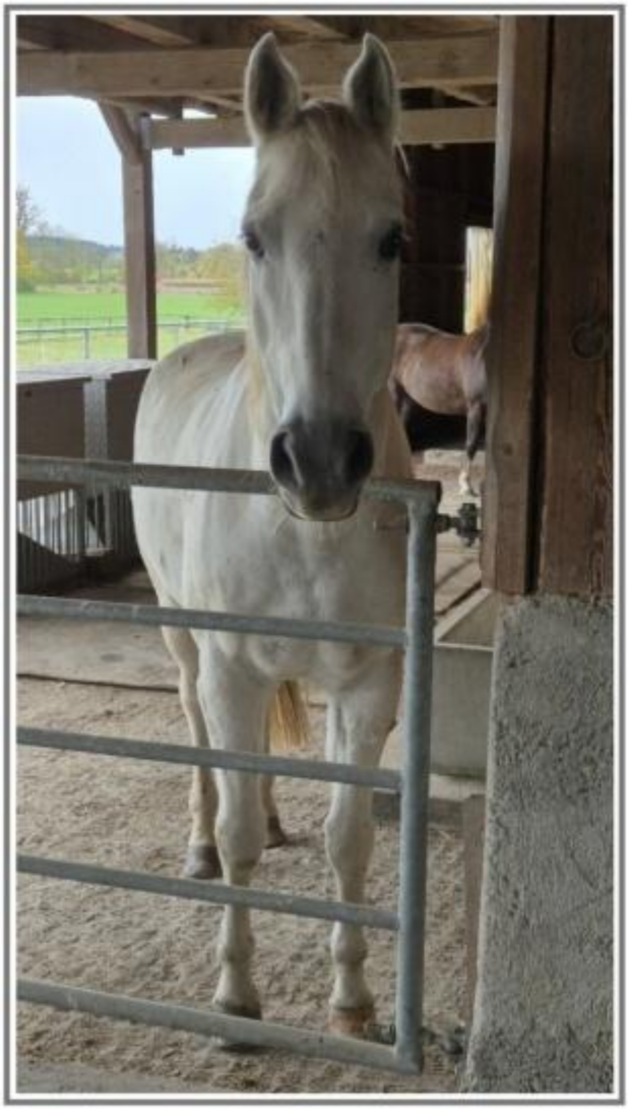

Supplementary Figure 41. *The gelding Case 8 in October 2025.*

3.8.3.3 Current status Case 8

The horse is in good health and good overall physical condition (Supplementary Figure 41), showing no signs of back pain, respiratory issues or discomfort. For the winter 2025, he will be exercised moderately at walk and trot while being transitioned to barefoot management. The goal of this phase is to evaluate whether adjustments in hoof balance and load distribution further improve general well-being, tendon condition, and performance stability.

3.8.4 Case 8 conclusion

This case shows that horses with extensive overload-related pathology can regain functional stability, coordination, and athletic usability when systemic reorganization is initiated during rehabilitation. After years of PSF^{-} development—including sacroiliac arthropathy, facet joint osteoarthritis, chronic back pain, and a suspensory branch lesion—the introduction of FIT facilitated a clear shift toward PSF^{+}, reflected in improved rhythm, trunk stability, and symmetrical movement. These changes enabled a successful return to mid-distance endurance competition.

The later recurrence of limb sensitivity following high intensity galloping work indicates persistent structural vulnerability and highlights the biological limits of full restitution in long-term performance horses. It further suggests that hoof mechanics may have constrained the regenerative potential during this period, as the horse remained shod throughout with hoof morphology not yet following the principles of PSF^{+} (Supplementary Figure 42). The upcoming winter conditioning phase will be employed to transition to barefoot management (Supplementary Figure 43), and the subsequent development of Case 8 could further support the investigation of limitations and capabilities of PSF^{+}, emphasizing the importance of synchronizing functional training with appropriate hoof balance to support long-term structural resilience.

Overall, this case demonstrates that PSF^{+} development can substantially restore function and performance even in high-level endurance horses with multi-regional pathology.

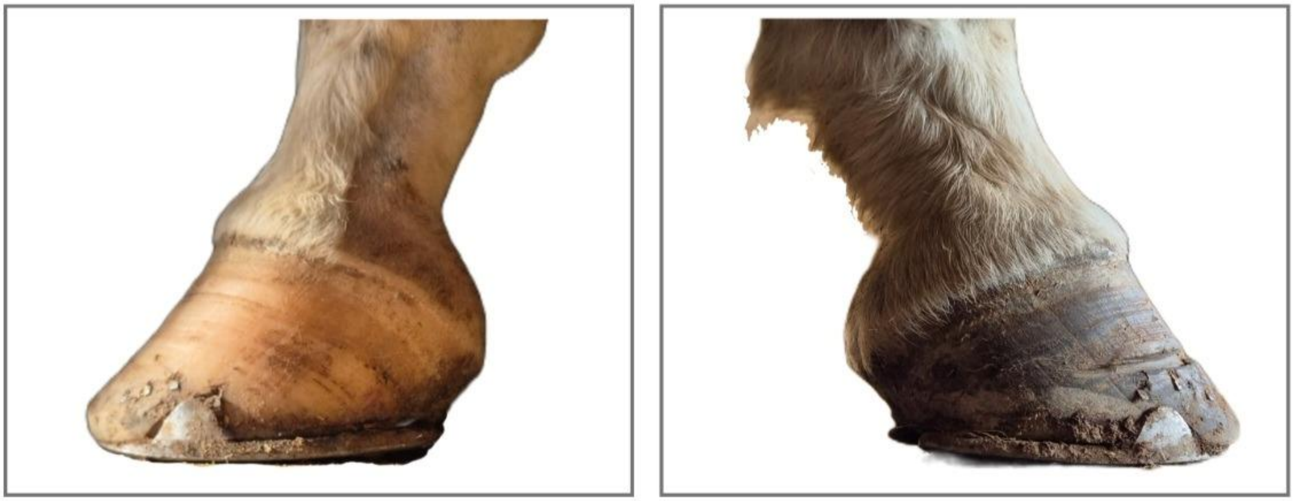

Supplementary Figure 42. *Lateral view of left (left panel) and right (right panel) fore hoof of Case 8 at the end of the shoeing cycle (early November 2025) right before transition to barefoot management.*

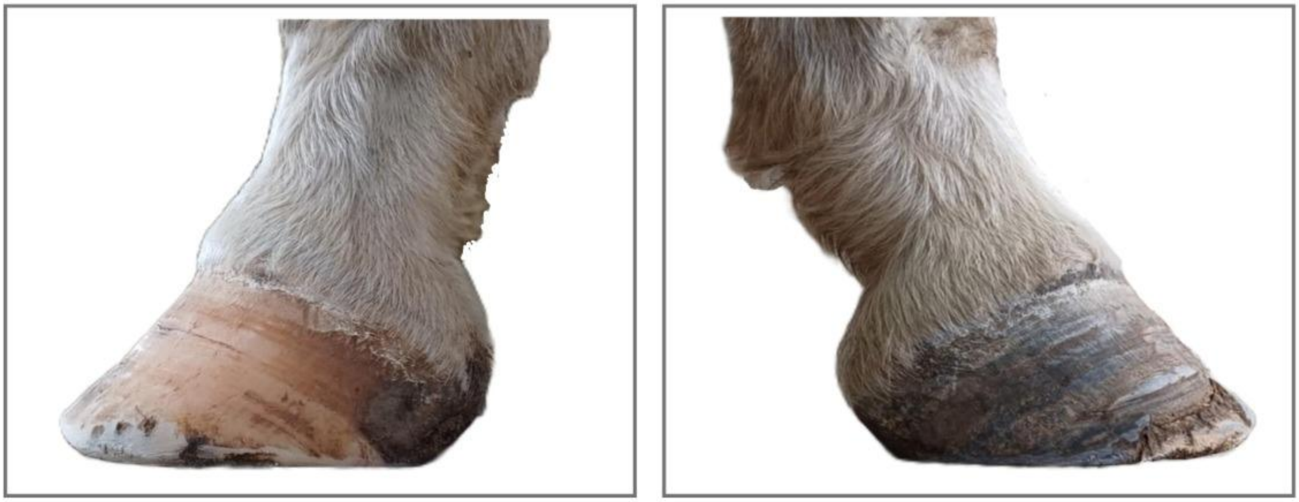

Supplementary Figure 43. *Lateral view of left (left panel) and right (right panel) forefoot of Case 8 after the first professional barefoot trim by Veterinarian Viviane Xenia Ulrich, hoofnerdyVet Natural Hoofcare (late November 2025). The toes will have to regrow. They have not been set back by the trimmer but are missing because of the former steepness and trim of the shoed hooves.*

3.9 Case Study 9: Reversal of a Croup-High Posture within a PSF$^+$ Framework under low Training Frequency and Intensity

Case 9 represents overbuilt horses whose conformation, often regarded as fixed anatomical expression, can demonstrably change within a PSF$^+$ process. The mare also exemplifies horses with low training intensity, as commonly found among companion horses, showing that postural improvement is achievable even with limited workload.

The Quarter Horse mare Case 9 was monitored from 2024 to 2025 during the transition from conventional management and low activity toward functionally organized training. Data was collected from longitudinal owner documentation including photographic documentation illustrating the transformation in musculature and body posture.

From 2021 until November 2024 the horse presented underweight, in poor musculoskeletal condition with cracked hooves and probably negative plantar angles. Also, the mare suffered collapse episodes of unclear origin. Initial treatments addressing presumed gastric discomfort and repeated changes in feeding rations showed no improvement. After conversion to barefoot management and later participation in structured training, the mare developed balanced musculature, improved stability, and behavioral calmness. Observable improvements included normalization of weight, reduction of the frequency of narcolepsy-like collapses, and the disappearance of overbuilt conformation.

Even in low-intensity training environments, it seems possible to reverse maladaptive postural organization and restore systemic stability. The case supports that PSF$^+$ processes depend not on training quantity but on the functional quality of interaction, confirming the importance of coordinated hoof management and self-organized movement for durable improvement.

3.9.1 General information on case study 9 subject

Case Identifier: Case 9
Date of birth: 28.03.2008
Breed: Quarter
Sex: Mare

3.9.2 Case 9 PSF$^-$ development

3.9.2.1 Case 9 PSF$^-$ history

2021: The mare was purchased from the Netherlands as a broodmare without pre-purchase examination. She had been kept under stable confinement and presented in poor nutritional status with marked muscular atrophy (Supplementary Figure 44). Prior to purchase, the horse had been trained for reining maneuvers such as circles, spins, and stops, but she did not share the previous owner's ambitions in competing, which led to her sale.

Both front hooves were shod and displayed vertical cracks extending to the coronary band; the hind hooves already showed indications of a negative plantar angle (retrospectively confirmed by radiographs taken in 2025). At her new home, the mare displayed nervous behavior and panicked when ridden outdoors.

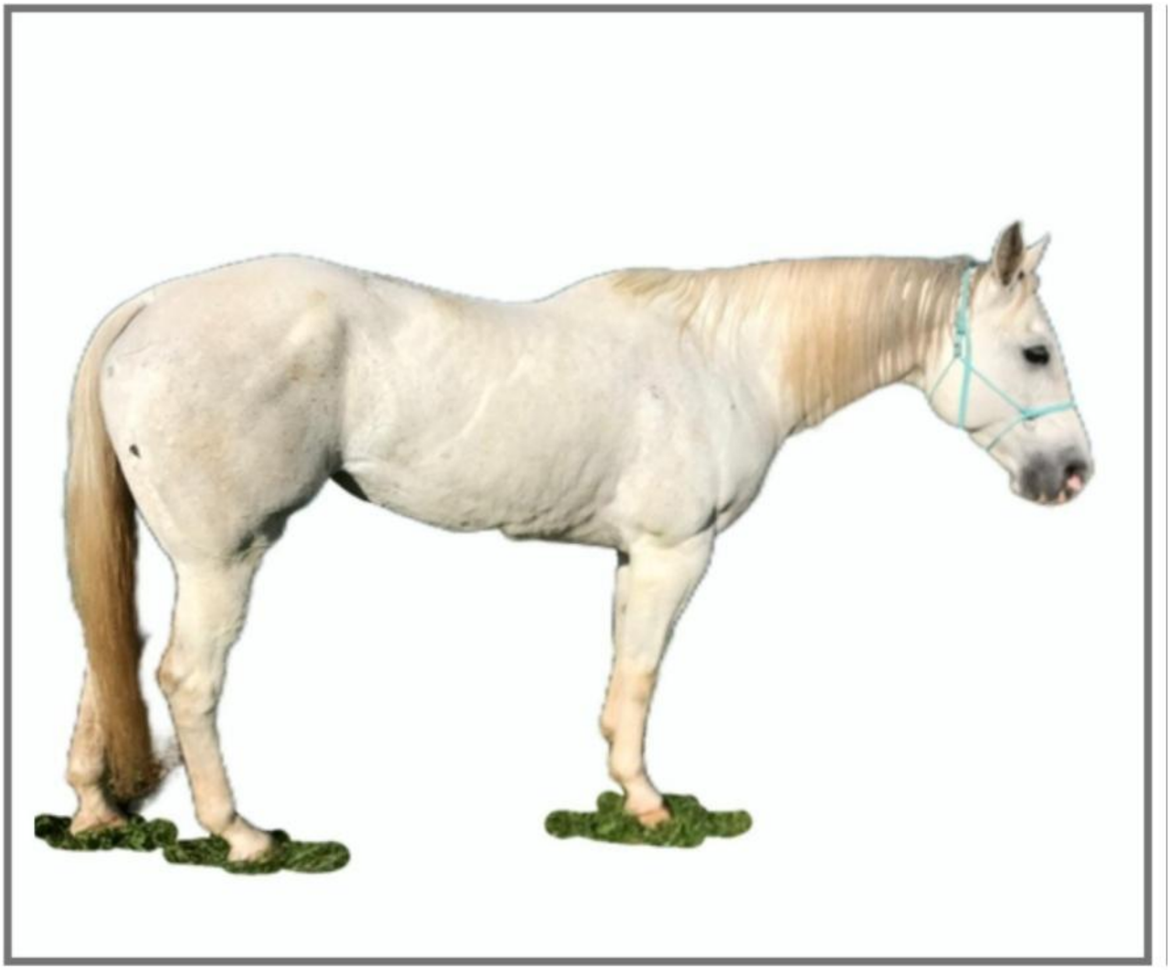

Supplementary Figure 44. *Body condition and posture of Case 9 after purchase in 2021.*

July 2021: The horse was moved from a box with paddock access to an open-stable system. Empirical treatment with omeprazole for suspected gastritis, various feed changes, and different housing forms provided no improvement; the mare remained underweight and nervous.

August 2021: The mare was bred but suffered early abortion in October 2021. During this period, collapse episodes became evident with frequent falls onto the carpal joints including severe skin lesions. The owner suspected at this point pseudo-narcolepsy as the origin of the collapses. Later that month, she transitioned to a paddock-trail environment (no improvement).

November 2021: Hoof management switched to barefoot management.

November 2021–May 2024: The mare was kept as a companion horse without further training and no changes in management.

3.9.2.2 Case 9 clinical findings

No clinical diagnostics were performed prior to the training intervention.

3.9.3 Case 9 development after introduction to PSF$^+$

3.9.3.1 Case 9 PSF$^+$ history

June 2024: The owner participated in one of the first author's 3-month courses and began transitioning to FIT. The mare learned to engage in horizontal contact to the bit, marking the first step toward functional self-organization.

November 2024: Under the guidance of Saskia Brieger, training sessions with in-person instructions began, combined with fascial release techniques using a fascia wheel, and a new hoof trimming approach. Training frequency increased from one to two weekly sessions to approximately three sessions per week by mid-2025.

Summer 2025: The owner participated in a multi-day in-person training session, focusing on postural stability and rhythmic locomotion in natural terrain.

November 2024–Summer 2025: The mare underwent a clear structural and behavioral transformation (Supplementary Figure 45). She gained weight, developed even musculature, and no longer appeared croup high. Behavioral relaxation and postural coordination improved markedly. Previously suspected gastric discomfort and collapse episodes diminished.

By summer 2025 Case 9 was able to sustain approximately 30 minutes of trail riding, including 5 minutes canter and 15 minutes trot, often also accompanying another horse as a lead horse. Despite this low workload, systemic organization and stability improved consistently, indicating that the quality of coordinated movement, not its intensity, determines the degree of PSF$^+$.

3.9.3.2 Current status Case 9

In October 2025, the mare is equipped with Western tack but ridden with taut reins and horse-initiated forward tension toward the bit instead of Western-typical loose reins. She presents in good mood with a stable posture and a healthy, well-regulated body condition. Training is temporarily paused due to an overly invasive hoof trimming procedure, and work will resume once hoof sensitivity and load tolerance have normalized.

3.9.4 Case 9 conclusion

The presented case shows that overbuilt posture in adult horses can be functionally modulated rather than being a fixed anatomical limitation. FIT facilitated a shift from maladaptive load distribution (PSF$^-$) toward integrated structural balance (PSF$^+$), even under moderate physical workload.

Improvements in posture, muscle texture, and stability were achieved without intensive exercise. The reduction of collapse episodes further indicates that functional load distribution and coordinated tension dynamics restored the function of the stay-apparatus. This case demonstrates that training focused on functional self-organization, even when applied at low frequency, can effectively reverse structural and behavioral dysfunctions. It emphasizes that long-term soundness depends on adaptive interaction between movement organization, hoof management and training quality rather than on training volume.

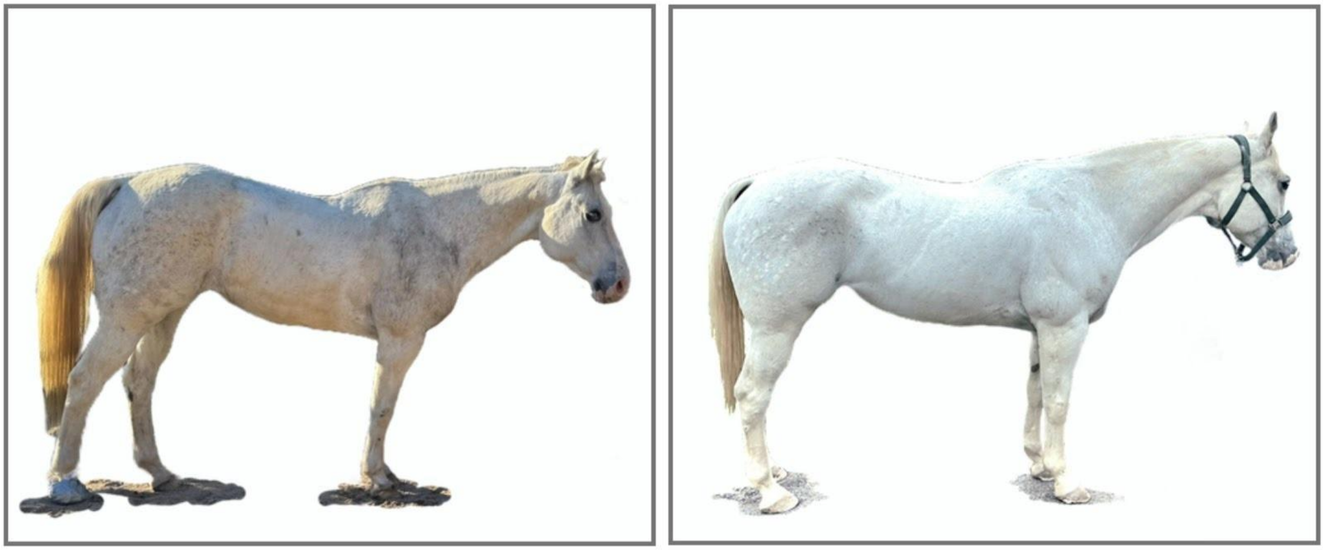

Supplementary Figure 45. *Transition from PSF^{-} to PSF^{+} of Case 9. Left panel: The horse on the first day of FIT (Nov 2024). Right panel: The mare in summer 2025 with visible improvements in posture and musculature.*

3.10 Case Study 10: A Reference Case of Progressive Structural and Functional Gain (PSF$^+$) in a High-Mileage Trail and TREC Horse

This case study illustrates a horse that never entered a trajectory of PSF$^-$ but instead developed within a stable pattern of PSF$^+$ from the beginning on. The gelding was trained exclusively with forward horizontal tension toward the bit, remained barefoot throughout his life so far, and accumulated substantial mileage across diverse terrain without developing musculoskeletal abnormalities. The horse–rider dyad followed a consistent practice of functional, interactive work in natural environments, focusing on exploration, movement, and shared experiences rather than corrective training. Over several years of intensive use in TREC competitions, trail riding, and multi-day excursions, the horse showed no clinical signs of overload, no postural decline, and no indications of systemic dysfunction. This case demonstrates that when horses are exposed early and consistently to functional self-organization under load—supported by appropriate hoof management and a coherent tension system—they may maintain structural integrity, behavioral stability, and long-term soundness even under substantial physical demands. Within the PSF framework, this case represents a distinct trajectory of continuous PSF$^+$, offering a reference profile for horses that develop robustness rather than decline. Notably, this case also documents a parallel developmental trajectory in the rider, whose transition to early precursor formats of FIT contributed substantially to the horse's continuous PSF$^+$ progression.

3.10.1 General information on case study 10 subject

Case Identifier: Case 10
Date of birth: 2016
Breed: Murgese
Sex: Gelding

3.10.2 Case 10 PSF$^-$ development

3.10.2.1 Case 10 PSF$^-$ history

Case 10 so far never showed signs of PSF$^-$.

3.10.2.2 Case 10 clinical findings

a. Colic Diagnosis by Veterinarian 1 (anonymized; 2021)

- Impaction colic
- Therapy: injection-based medical therapy

b. Colic Diagnosis by Veterinarian 2 (anonymized; 2024)

- Dehydration colic during a TREC competition
- Treatment: analgesics and fluid therapy, the following day cleared to start again

3.10.3 Case 10 development after introduction to PSF$^+$

3.10.3.1 Case 10 PSF$^+$ history

2016: born in Italy

2016–April 2019: Case 10 grew up under natural, age-appropriate conditions without any indications of early structural or functional problems. In spring 2019, shortly before purchase, he sustained a minor accident that resulted in a superficial injury and a permanent scar on the hindquarters, with no lasting functional impairment.

2019: Following his purchase in April, the horse underwent a gradual acclimatization period. Initially, he accompanied the owner's riding horse as a hand horse on trail rides. Over time he transitioned to being ridden in the group and showed no difficulties adjusting to his new role.

2020: The first solo ride with his owner took place on the turn of the year. The rest of the year was marked by a forced break with only light work for the horse, as the owner experienced an injury.

2021: With the resumption of regular work primarily outdoors and sometimes in the arena or in the round pen, the following activities were added:

- First off-site clinics
- A multi-day riding holiday in the Palatinate in Germany
- First camping trip
- Riding at the beach in Zeeland, Netherlands
- Participation at a TREC event where the owner served as a judge, allowing the horse to become familiar with the competitive environment
- Some few training sessions in the riding arena

The owner came from a corrective, effort-based riding-style which she had been practicing for many years before she participated in early pilot versions of the online training courses that later evolved into the current instructional framework. Throughout the observation period, the rider underwent a marked conceptual and technical shift, which became an integral component of the system's development. Although she had previously trained with considerable dedication within other riding traditions—often noting that "the more I practiced, the worse the results became"—her engagement in early precursor formats of FIT initiated a systematic reorganization of her own timing, movement strategies, and interaction with external forces. During this process, each fallback to old riding habits was immediately responded to by Case 10 by showing dysfunctional movement patterns (Supplementary Figure 46). The communication between horse and rider and breaking dysfunctional movement-patterns were key components of the couple's development. The rider translated conceptual material into practical experimentation, documented progress through repeated recordings, and used structured workbooks to refine her observations. This evolving rider-side organization consistently reinforced the horse's capacity for functional self-organization under load and appears to have been a decisive factor in maintaining an uninterrupted PSF^+ trajectory. The gelding responded positively to this structured and reflective approach, which facilitated the clear integration of new concepts into his daily work.

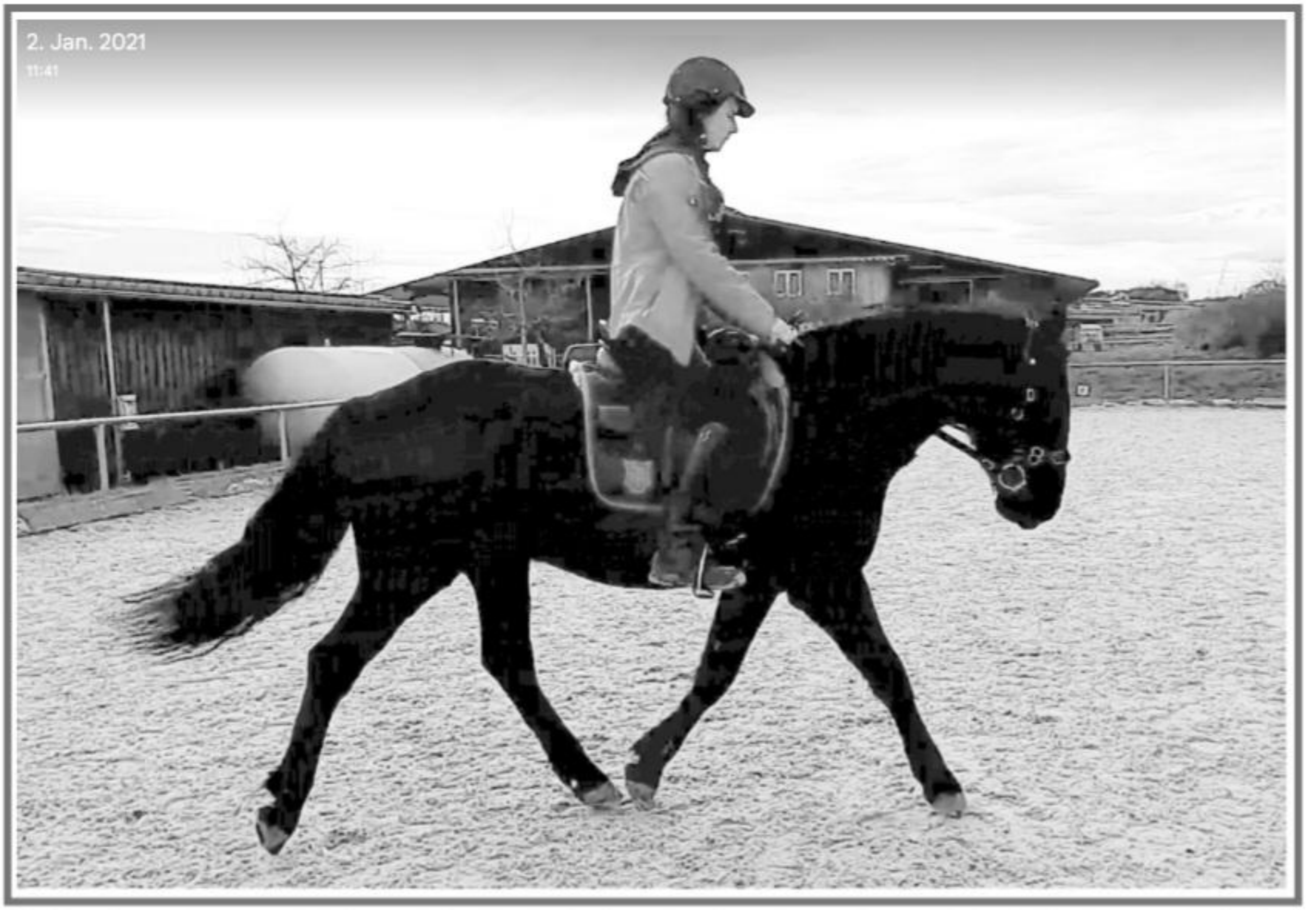

Supplementary Figure 46. *Case 10 in the arena (January 2021). Old habits of the rider become visible in the footing pattern of the horse. The left fore remains longer on the ground than the diagonal hind hoof.*

Horse and rider maintained a high activity level throughout the following years:

- 2022:
  - First TREC competition season, starting in Luxembourg (T 20)
  - Another riding holiday in the Palatinate
  - Extended stay in Zeeland, Netherlands
  - TREC training session in France
- 2023:
  - TREC competition (T 20) Netherlands
  - TREC competition (T 20) Luxembourg
  - Extended stay in the Palatinate including extreme-trail training
  - TREC training session in France
  - Zeeland vacation
  - TREC training in the Black Forest in Germany
  - TREC competition (T 20) in Züsch, Germany
- 2024:
  - TREC competition (T 20) Luxembourg (second colic episode occurred)
  - Trail ride holiday in the Palatinate in Germany

  - Vacation in Zeeland in the Netherlands
  - TREC competition (T 30) Black Forest in Germany
  - Military training in Leonberg
  - TREC competition (T 20) in Züsch, Germany
  - 10 day stay trail riding near Bad Kreuznach, Germany
- 2025
  - Trail ride holiday in the Palatinate in Germany (Supplementary Figure 47–Supplementary Figure 49)
  - Vacation Zeeland, Netherlands
  - TREC competition (T 20) in Luxembourg
  - TREC training in Züsch, Germany

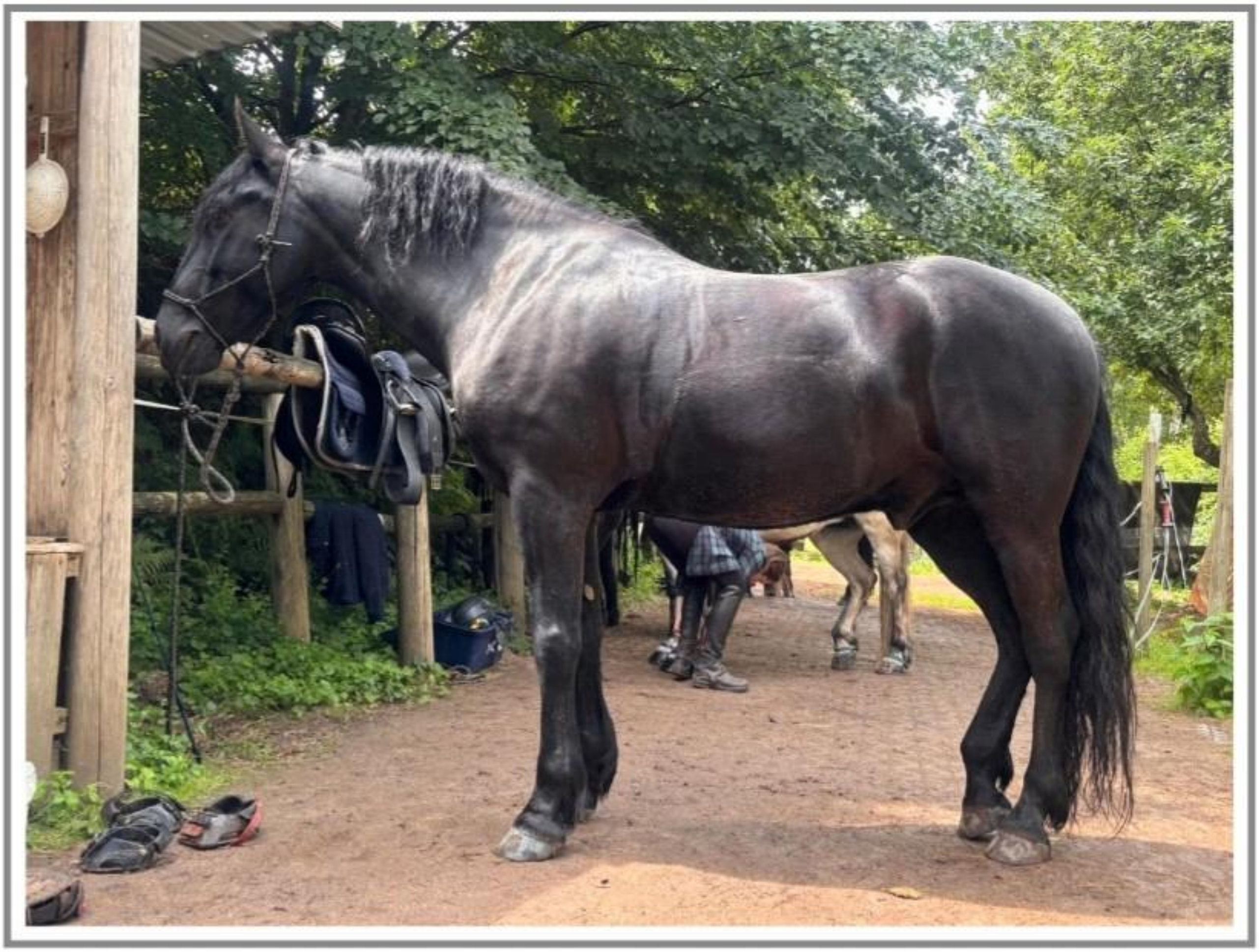

Supplementary Figure 47. *Case 10 on active holiday in Summer 2025 after a ride.*

3.10.3.2 Current status Case 10

In November 2025, Case 10 is doing trail rides at home, training for the next year's activities. The horse is overall sound, strong and willing to work.

3.10.4 Case 10 conclusion

This case presents an example of a horse that has maintained complete musculoskeletal soundness over several consecutive years of substantial physical use, including long-distance riding, varied terrain, and repeated TREC competitions. Although such long-term robustness is widely valued in equine training and management, horses that maintain this level of integrity are not often documented in everyday practice.

The continuous PSF^+ trajectory observed in this gelding suggests that early and consistent preservation of functional self-organization under load may help reduce the risk of structural or functional loss that can develop over a horse's lifetime. In this case, coherent tension toward the bit, functional interaction in natural environments, and lifelong barefoot management appear to have created conditions under which the horse's physiological integrity remained stable over time (Supplementary Figure 48 & Supplementary Figure 49).

An additional distinguishing feature of this case is the rider's own parallel development, which contributed directly to the stability of the PSF^+ trajectory. Her transition from corrective, effort-based training approaches to function-oriented interaction appears to have created conditions under which the horse never entered a PSF^- cycle, suggesting that long-term robustness may depend as much on rider-side organization and attitude as on equine factors.

While this single case cannot establish causation, it indicates that further exploration is warranted to understand why such favorable patterns of long-term soundness are not more consistently observed. Within the PSF framework, it offers a reference profile for understanding how uninterrupted functional organization may support sustained equine health.

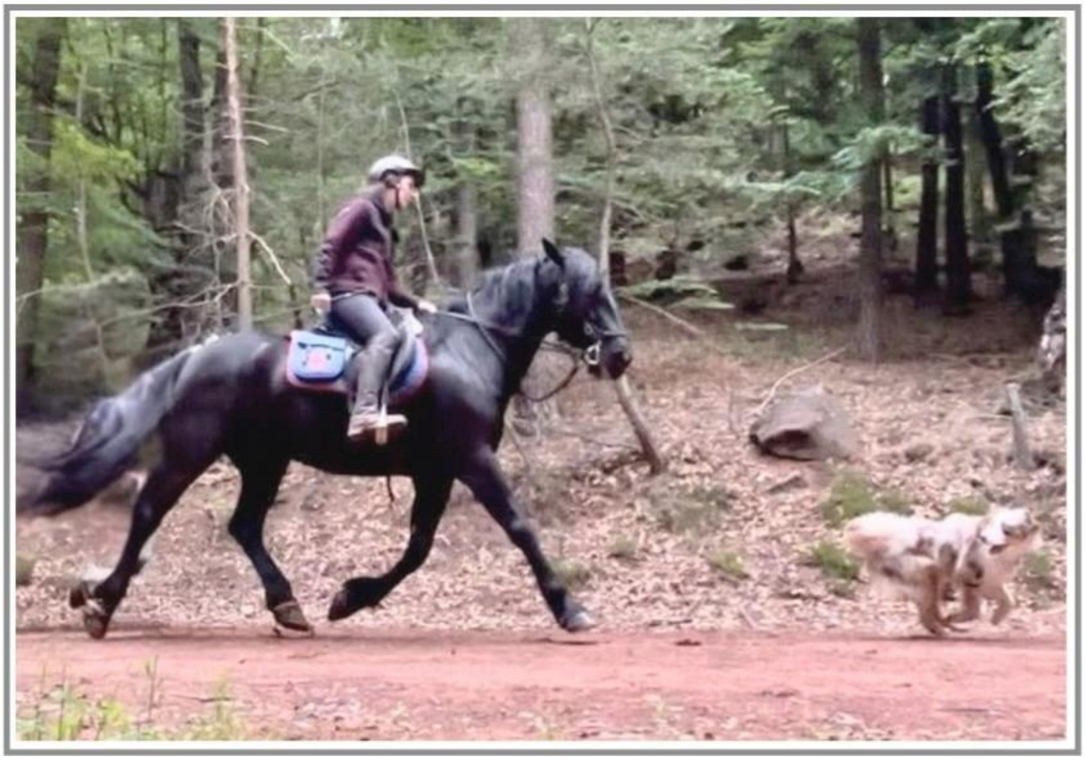

Supplementary Figure 48. *Case 10 in trot with a reverse (non-pathologic) phase-shift, probably due to the soft slope downhill and the very energetic "flying" forehand.*

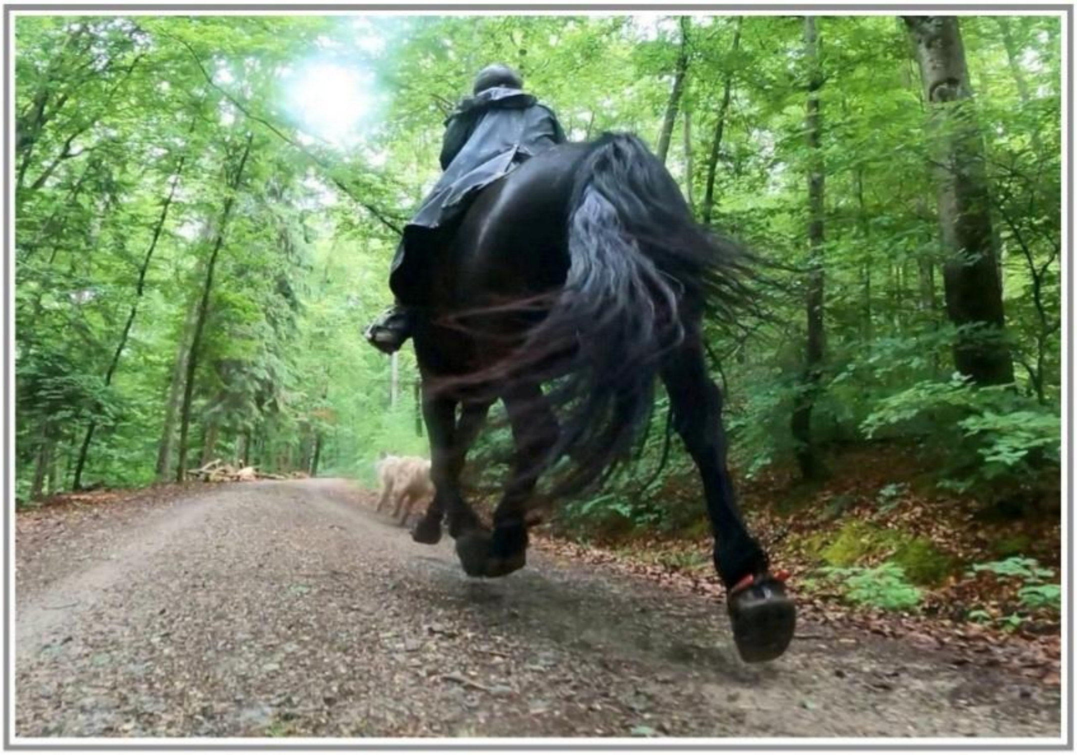

Supplementary Figure 49. *The suspension phase from a different perspective, remarkable the full stretch moment of the right lateral.*

4 Author Biographies

Maren Diehl has more than 30 years of practical experience with horses. Her work focuses on functional anatomy and the organization of movement in horses and riders, informed by theoretical models of self-organization and biotensegrity. Since 2020, she has conducted three-month online training programs that generated practice-based evidence through structured participant documentation and follow-up evaluation. Her approach combines empirical field observation with conceptual development aimed at improving the understanding of the equine body as a self-organizing system capable of structural and functional adaptation.

Katharina Bader holds a doctorate in Physical Chemistry and works as an ISTQB-certified software test engineer in the automotive sector. Her expertise centers on the structured analysis and interpretation of complex datasets, which she also applies in interdisciplinary research contexts. As a lifelong equestrian, she integrates practical experience with system-analytic thinking in her contributions to equine research.

The authors conducted this work independently and outside their primary employment. No financial support, remuneration, or dedicated project funding was provided.
