# Supplementary figures and images for: Progressive structural and functional change in horses: a conceptual framework for systemic equine (patho-)physiology
Source: Front Vet Sci. 2026 Apr 7;13:1767386. doi: 10.3389/fvets.2026.1767386 (PMC13095508; doi:10.3389/fvets.2026.1767386)

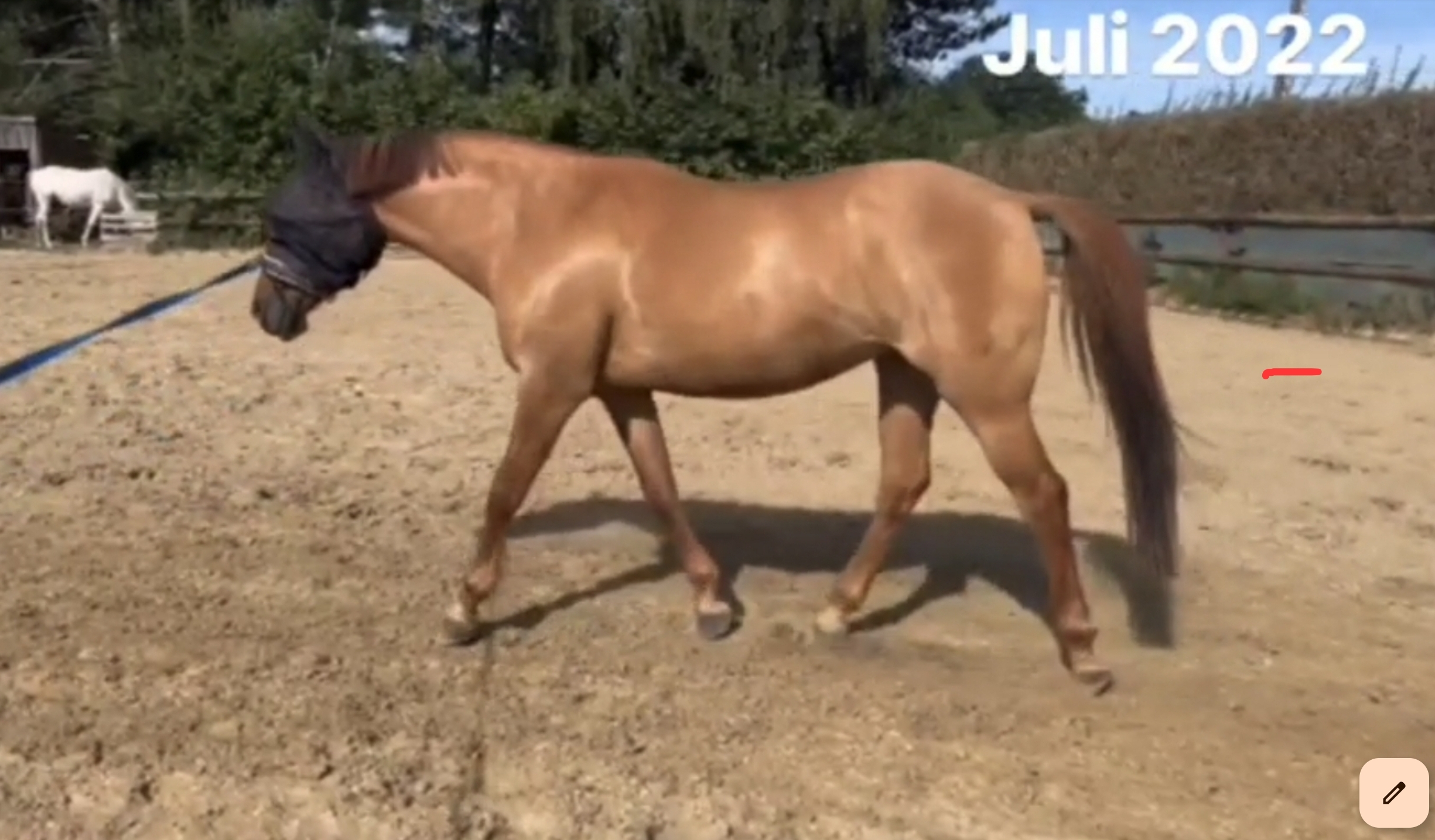

Supplement: Supplementary file 2 [file Data_Sheet_2.zip › Raw Data Main Text/Fig_10_1_case_5.jpg]

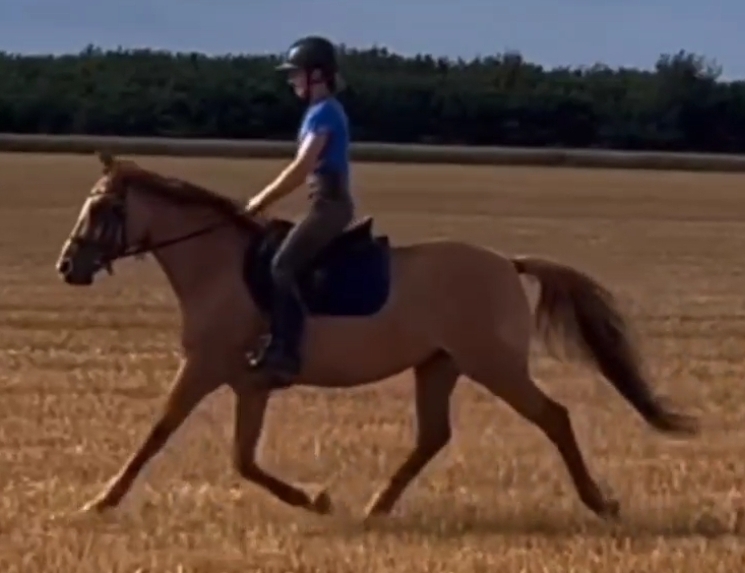

Supplement: Supplementary file 2 [file Data_Sheet_2.zip › Raw Data Main Text/Fig_10_2_case_5.jpg]

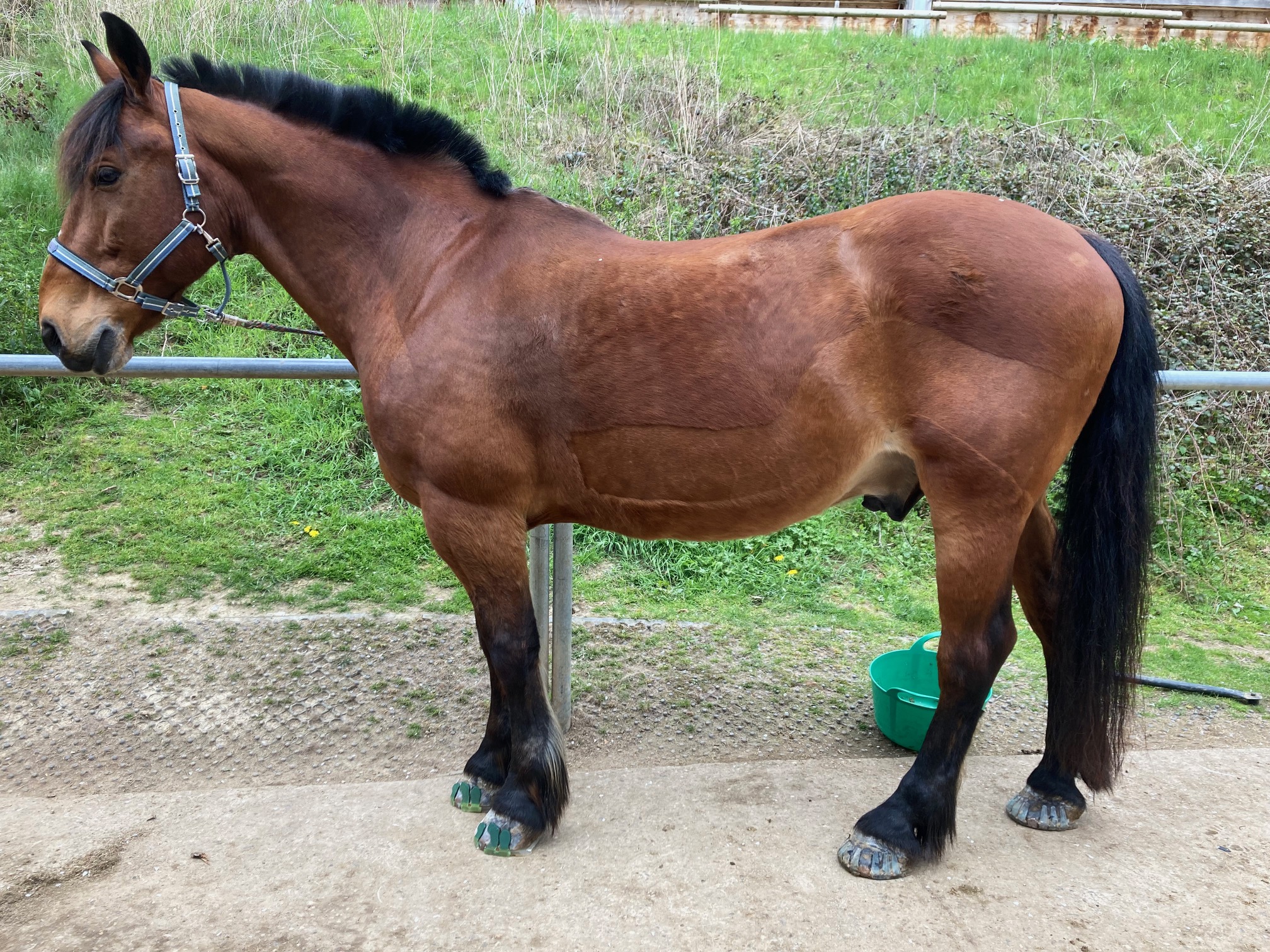

Supplement: Supplementary file 2 [file Data_Sheet_2.zip › Raw Data Main Text/Fig_1_1_no_case.jpg]

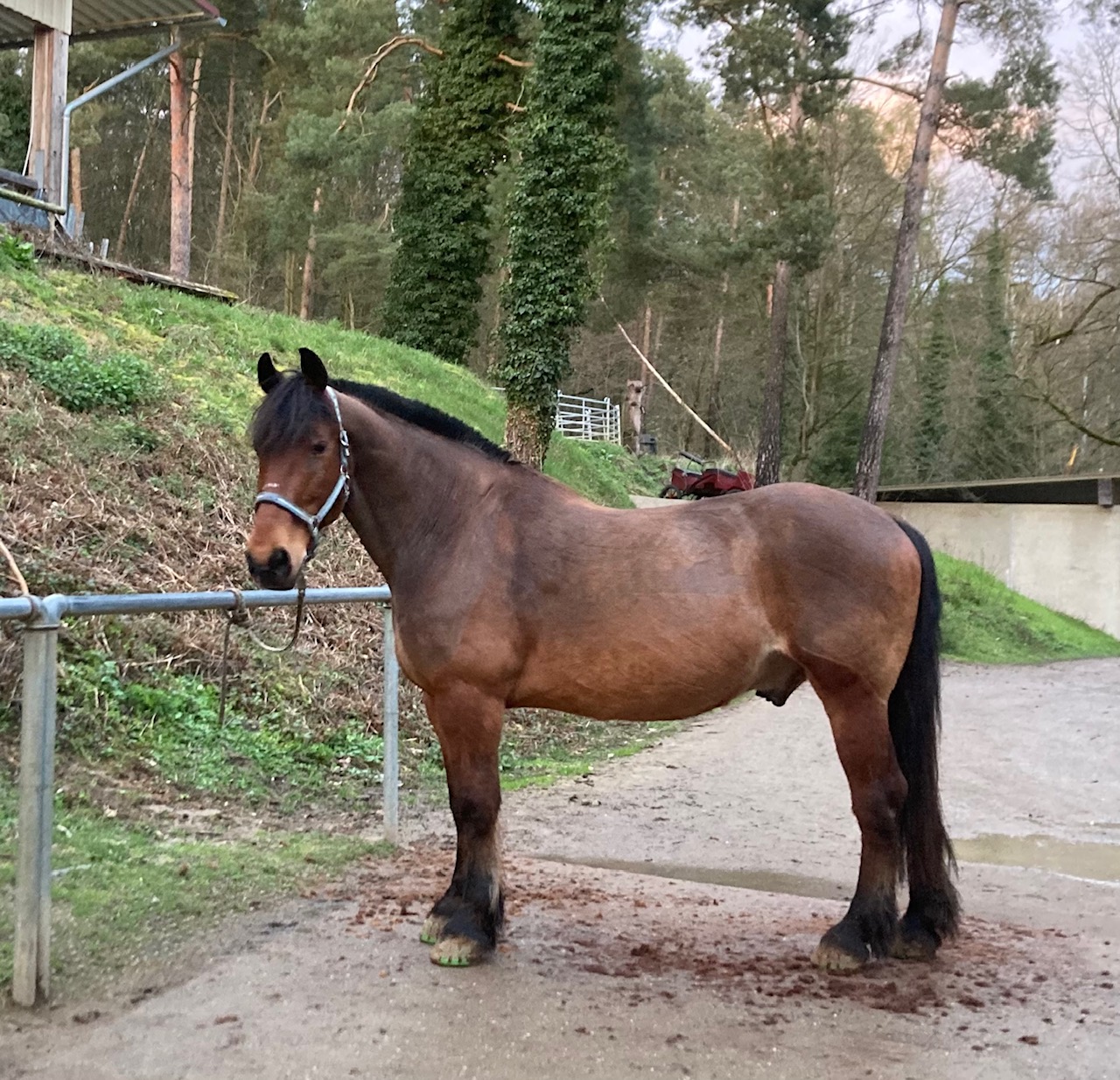

Supplement: Supplementary file 2 [file Data_Sheet_2.zip › Raw Data Main Text/Fig_1_2_no_case.jpg]

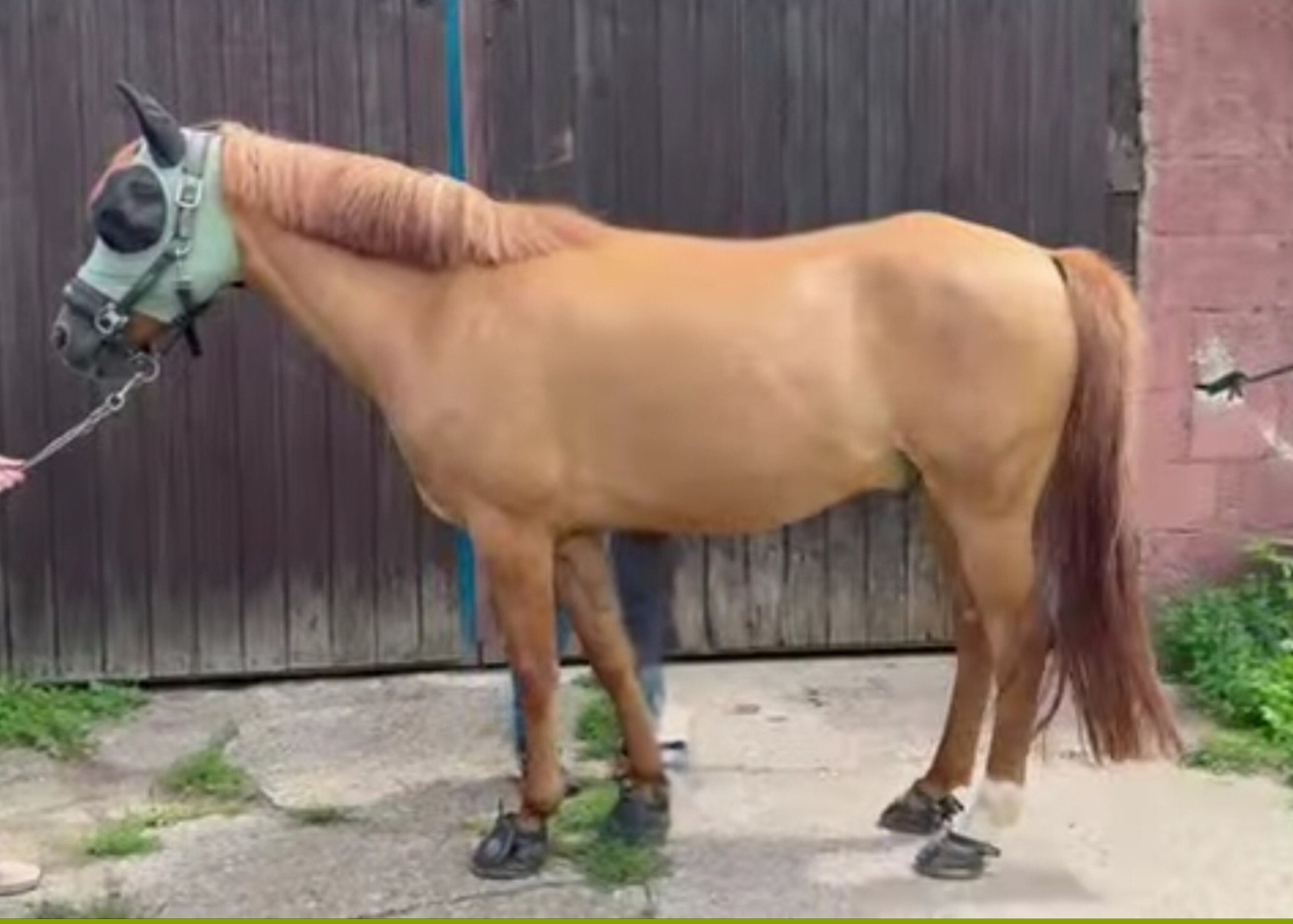

Supplement: Supplementary file 2 [file Data_Sheet_2.zip › Raw Data Main Text/Fig_2_Case_1_1.jpg]

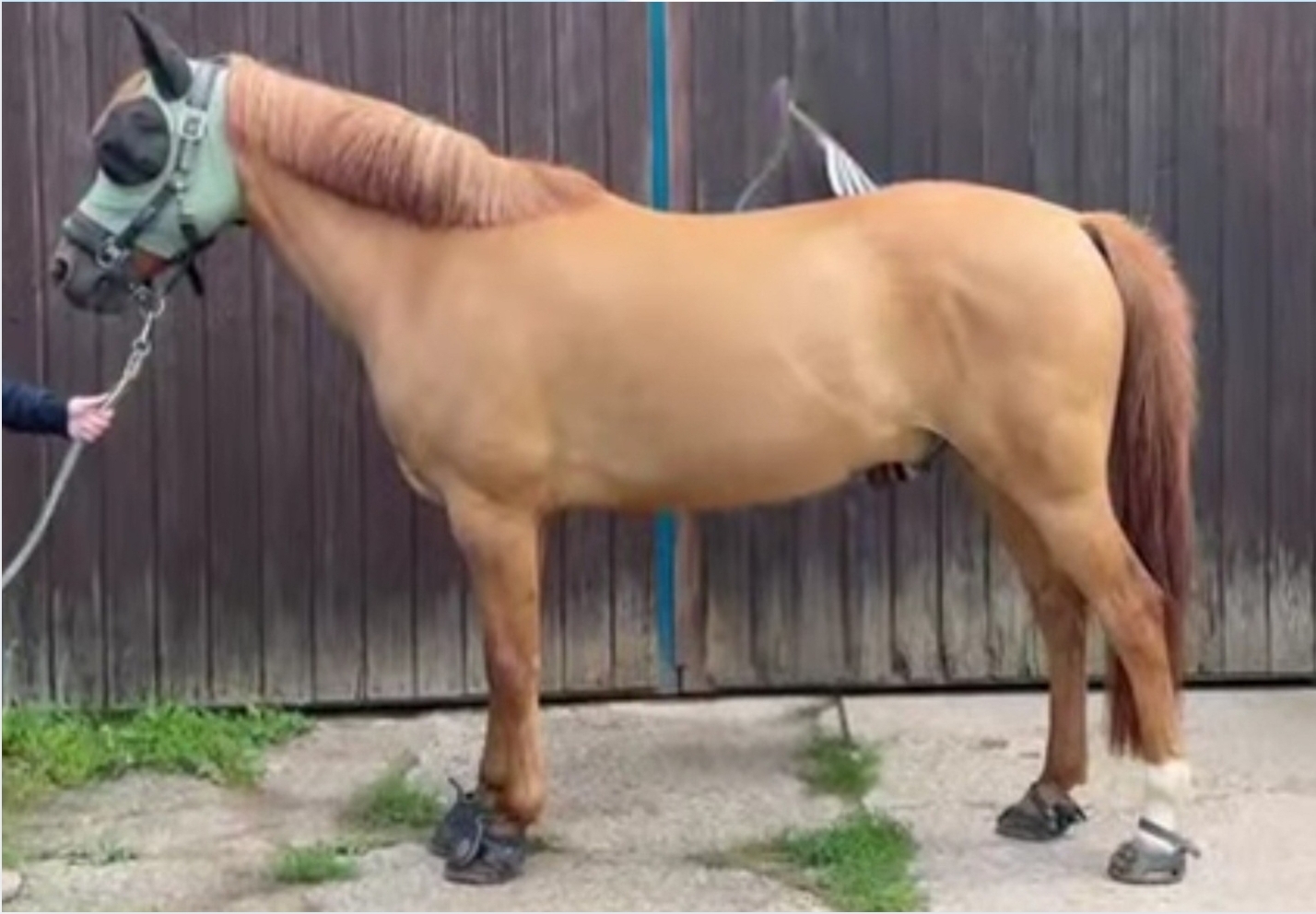

Supplement: Supplementary file 2 [file Data_Sheet_2.zip › Raw Data Main Text/Fig_2_Case_1_2.jpg]

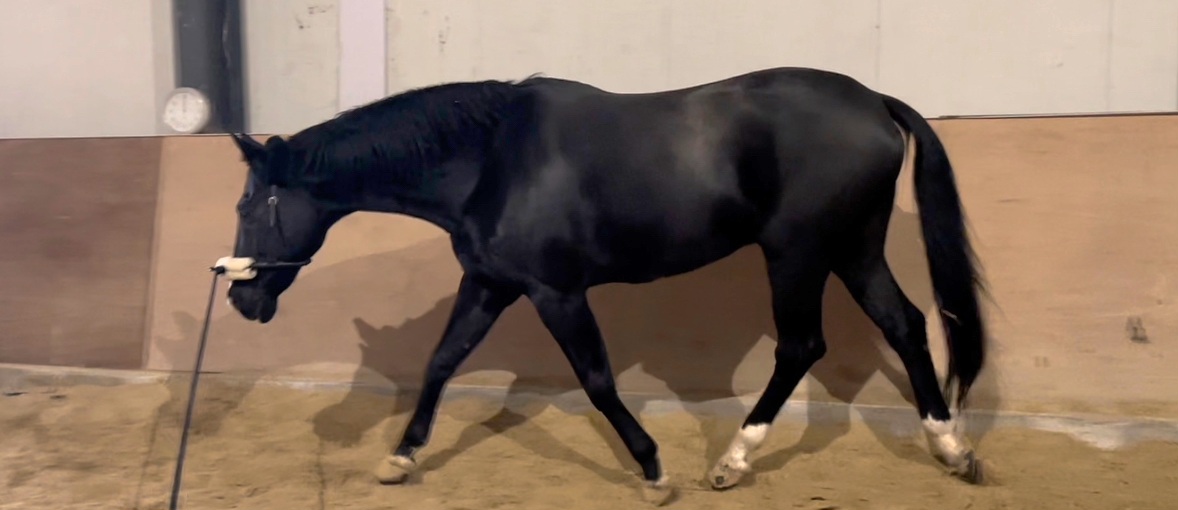

Supplement: Supplementary file 2 [file Data_Sheet_2.zip › Raw Data Main Text/Fig_3_1_no_case.jpg]

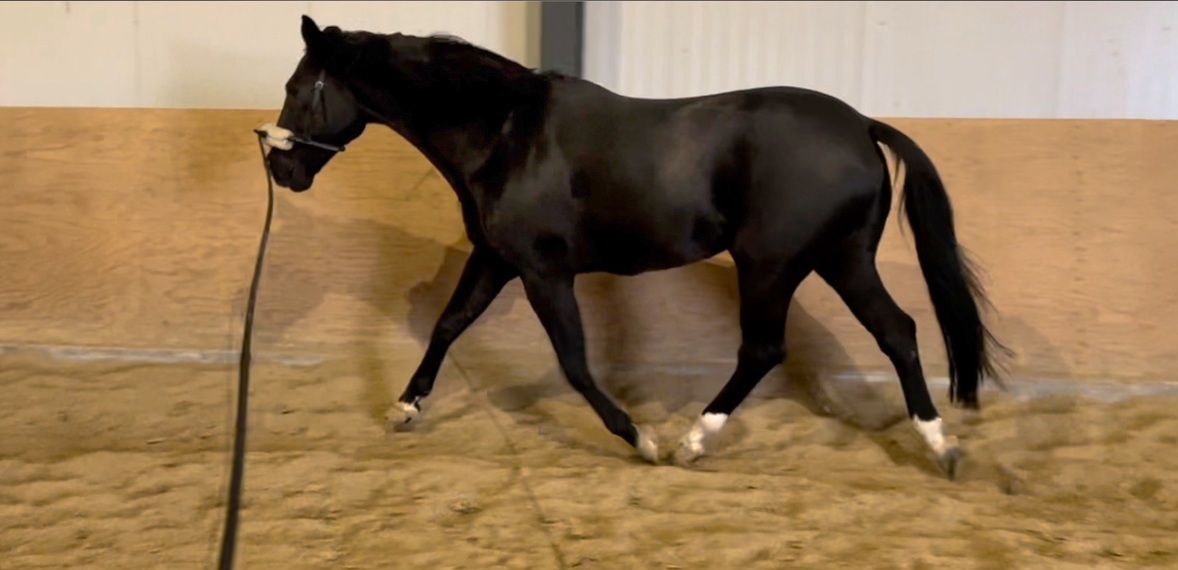

Supplement: Supplementary file 2 [file Data_Sheet_2.zip › Raw Data Main Text/Fig_3_2_no_case.jpg]

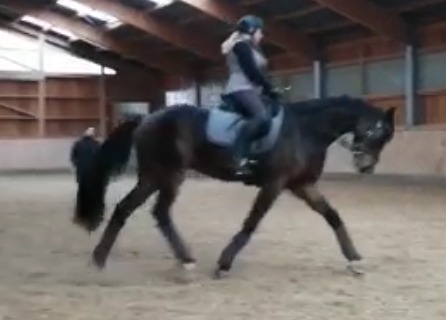

Supplement: Supplementary file 2 [file Data_Sheet_2.zip › Raw Data Main Text/Fig_4_1_case_2.jpg]

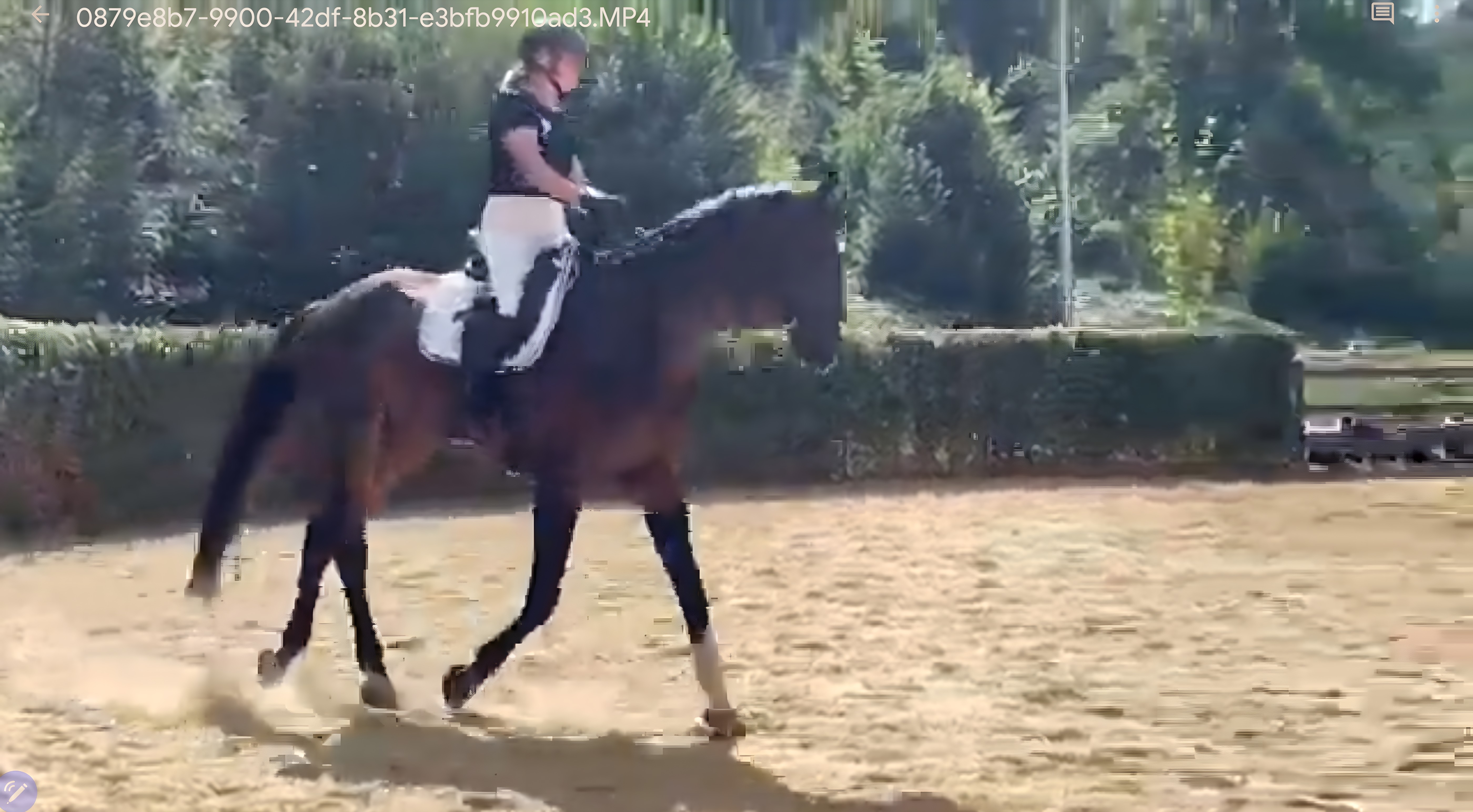

Supplement: Supplementary file 2 [file Data_Sheet_2.zip › Raw Data Main Text/Fig_4_2_case_2.jpg]

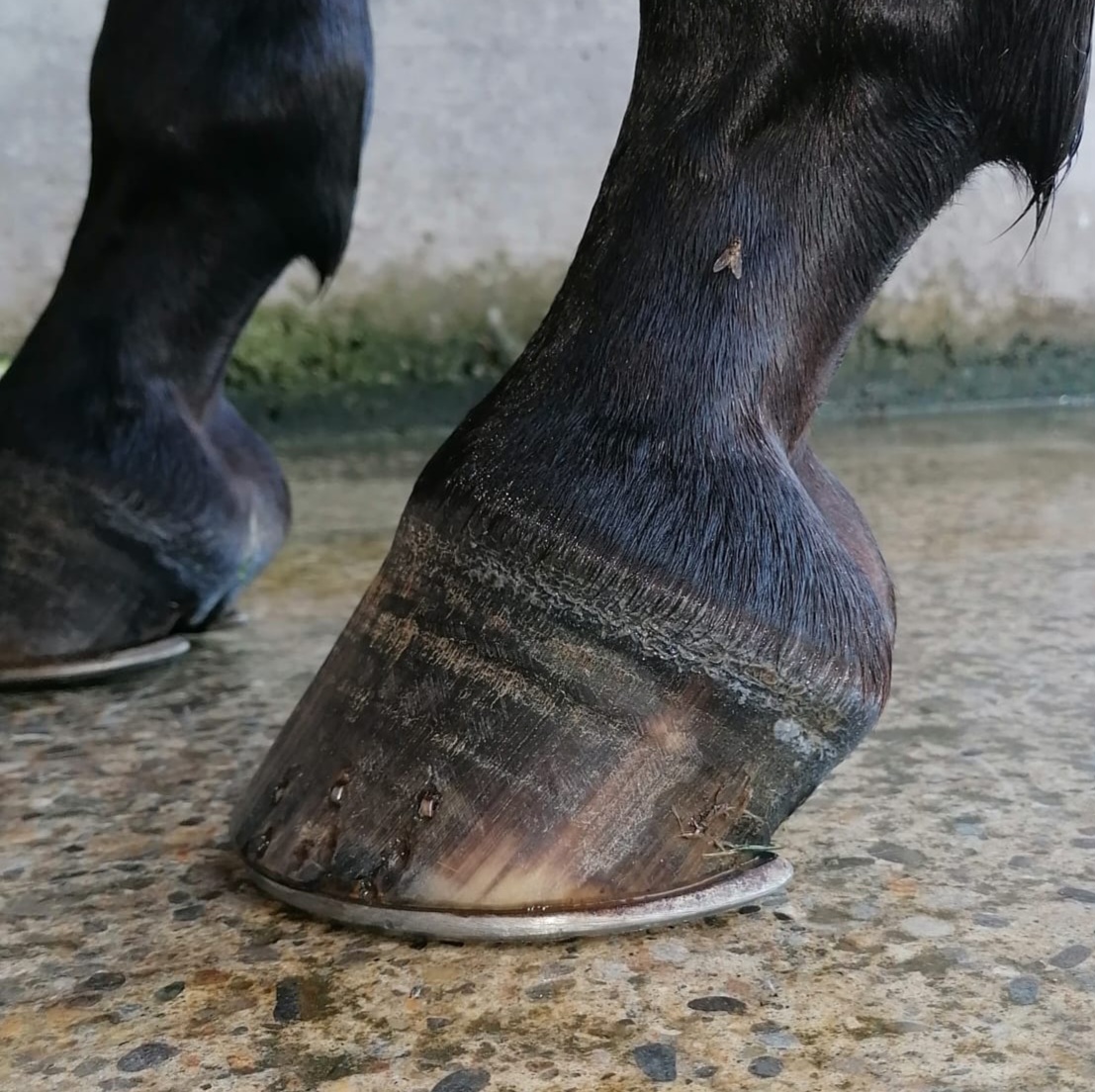

Supplement: Supplementary file 2 [file Data_Sheet_2.zip › Raw Data Main Text/Fig_5_1_case_7.jpg]

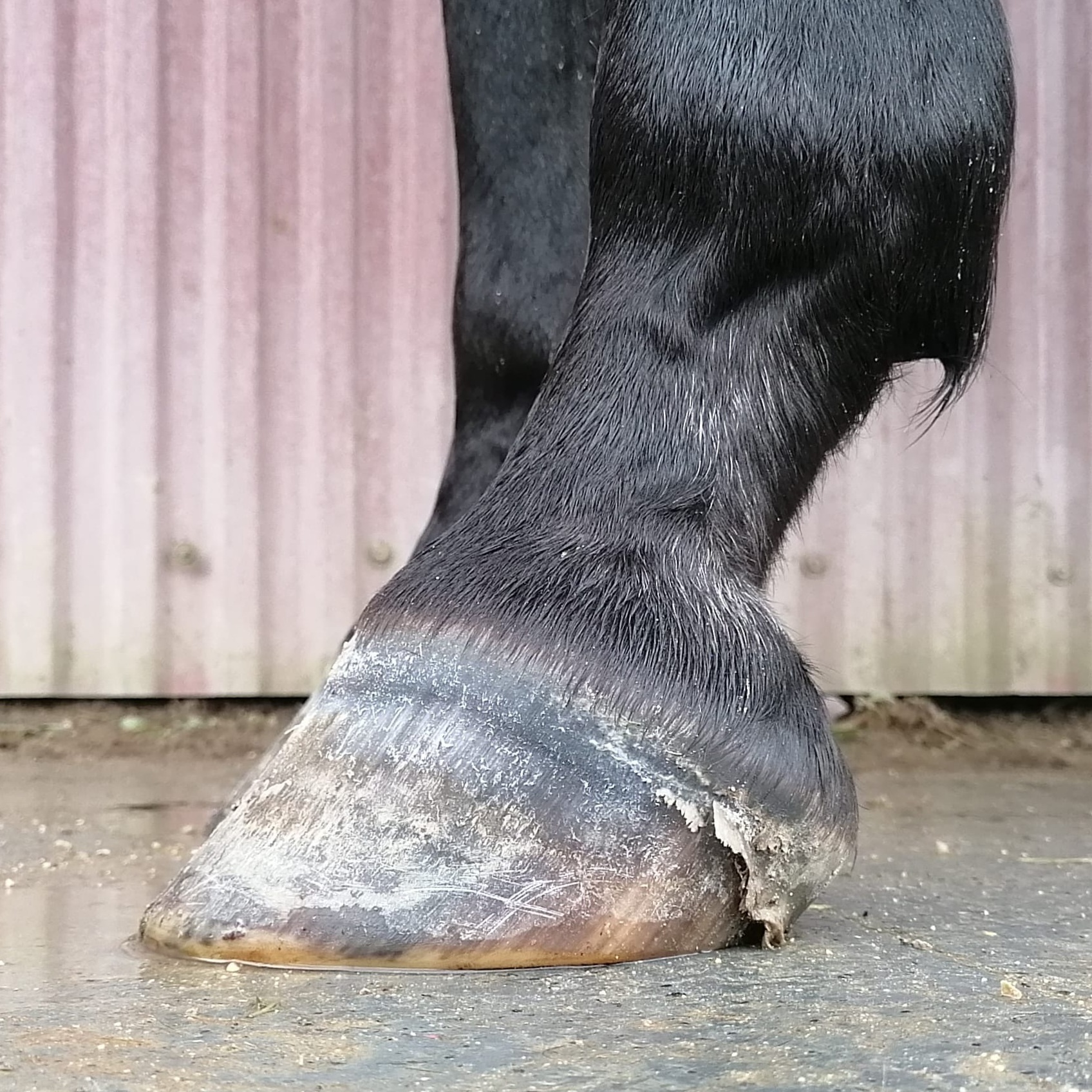

Supplement: Supplementary file 2 [file Data_Sheet_2.zip › Raw Data Main Text/Fig_5_2_case_7.jpg]

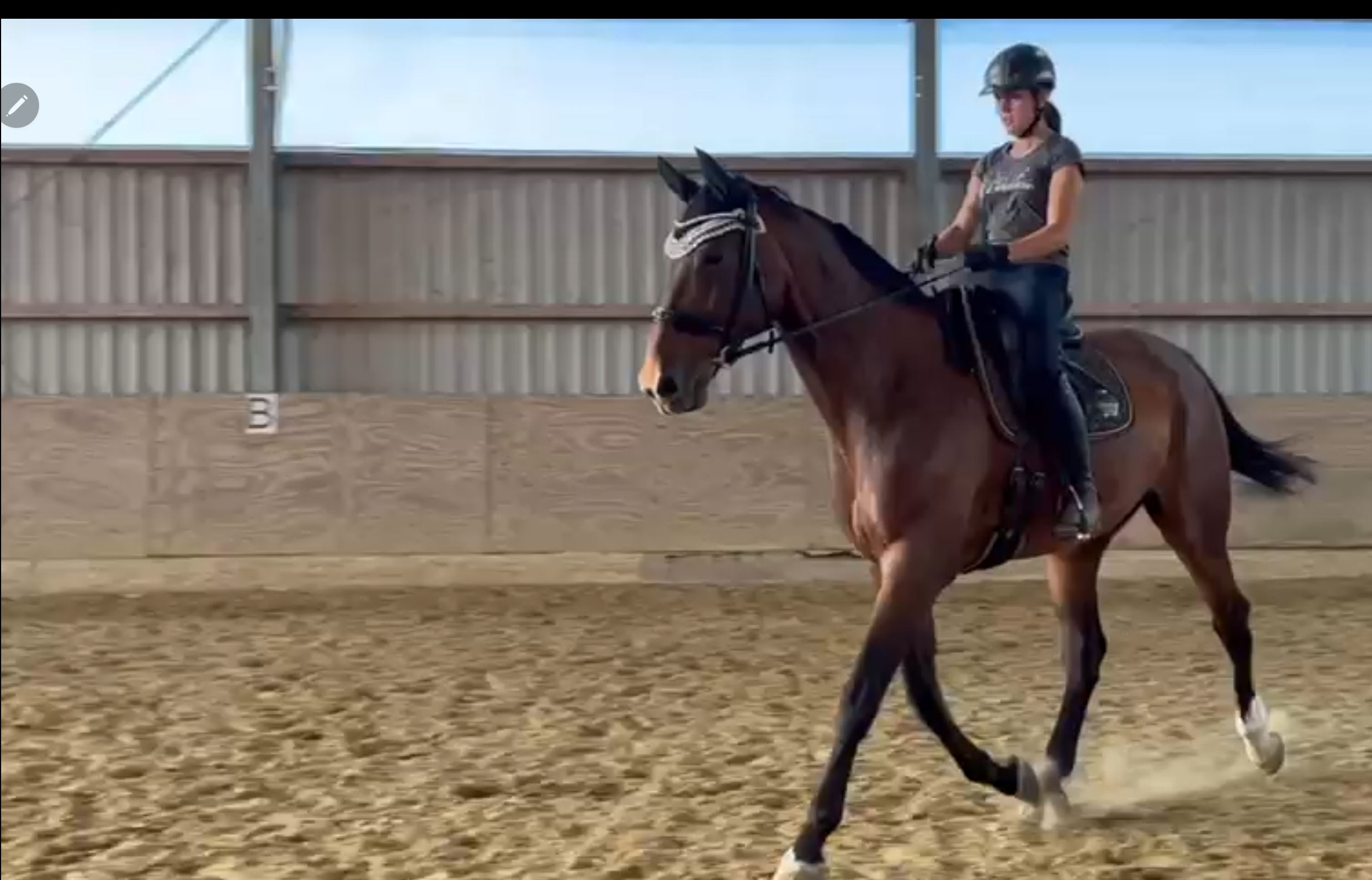

Supplement: Supplementary file 2 [file Data_Sheet_2.zip › Raw Data Main Text/Fig_6_1_case_3.jpg]

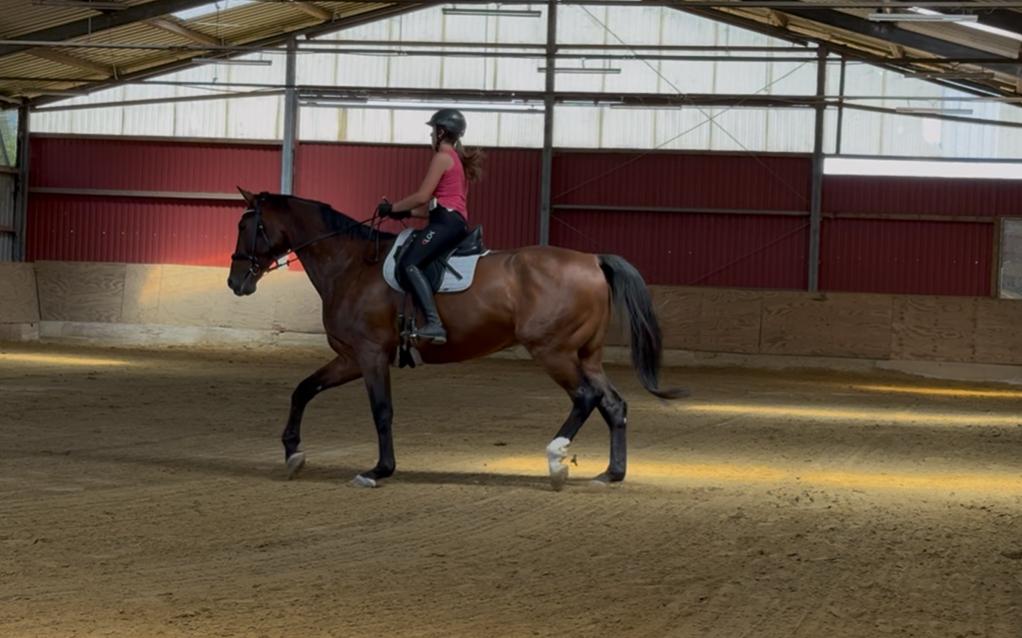

Supplement: Supplementary file 2 [file Data_Sheet_2.zip › Raw Data Main Text/Fig_6_2_case_3.jpg]

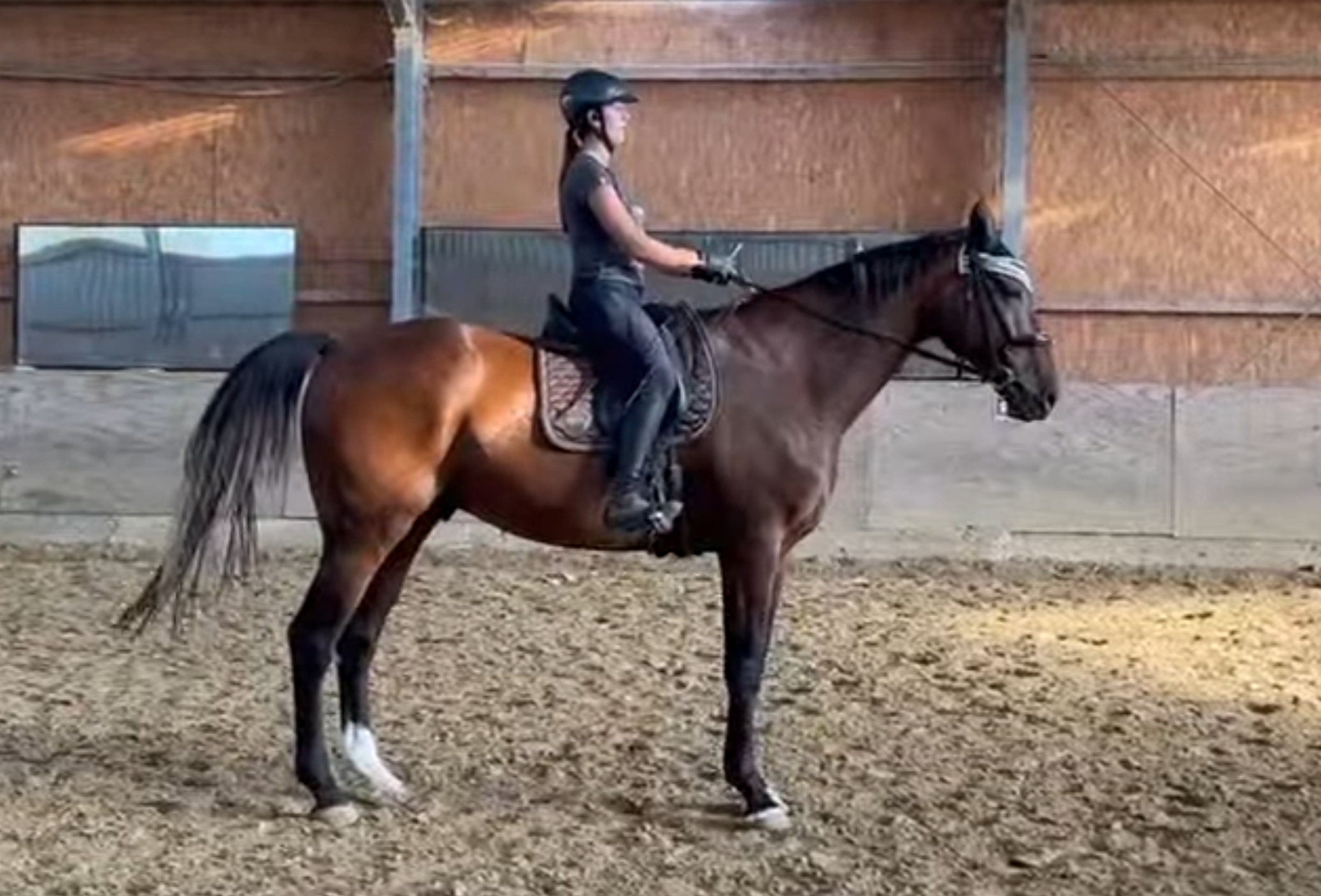

Supplement: Supplementary file 2 [file Data_Sheet_2.zip › Raw Data Main Text/Fig_8_1_case_3.jpg]

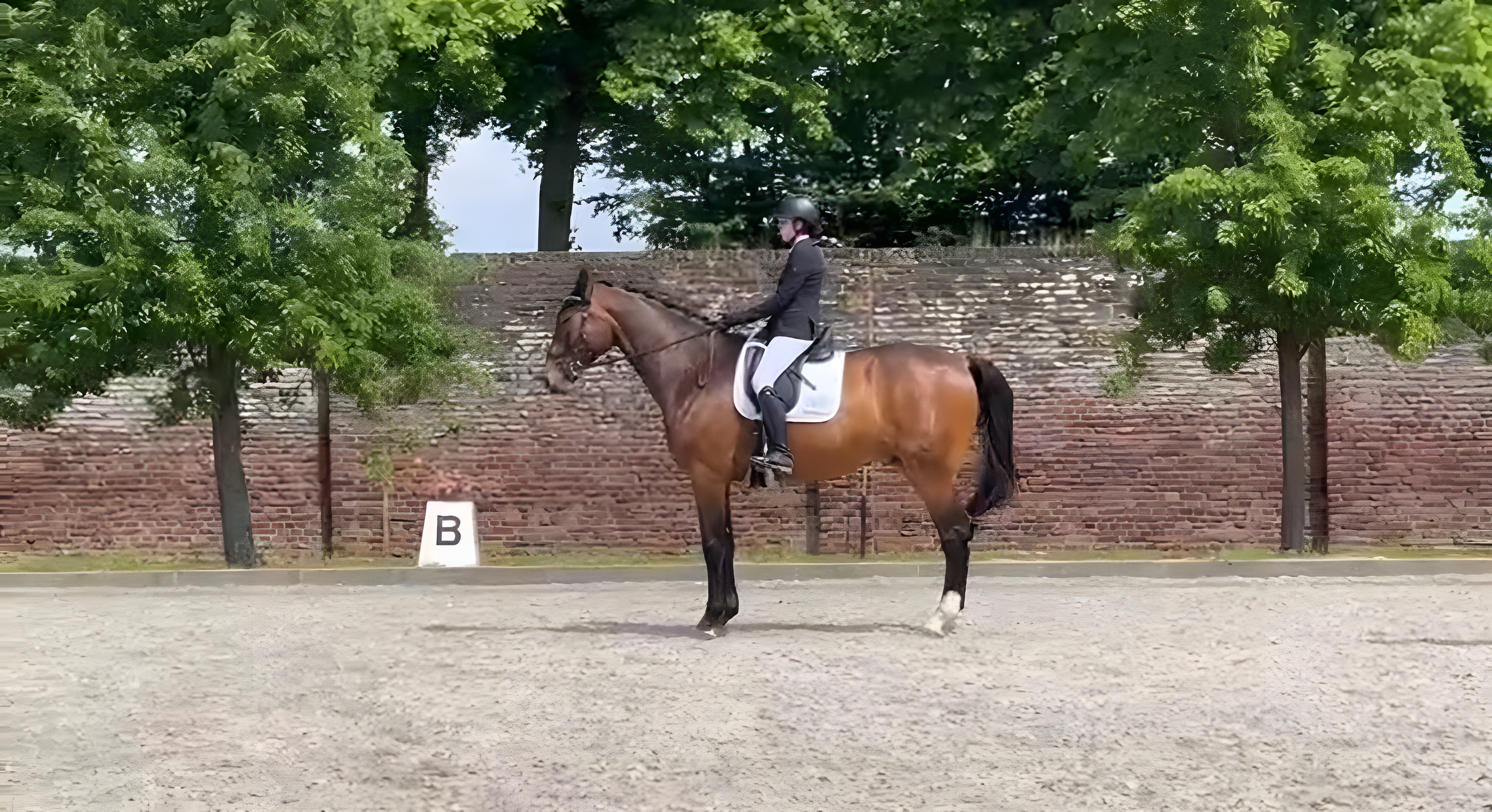

Supplement: Supplementary file 2 [file Data_Sheet_2.zip › Raw Data Main Text/Fig_8_2_case_3.png]

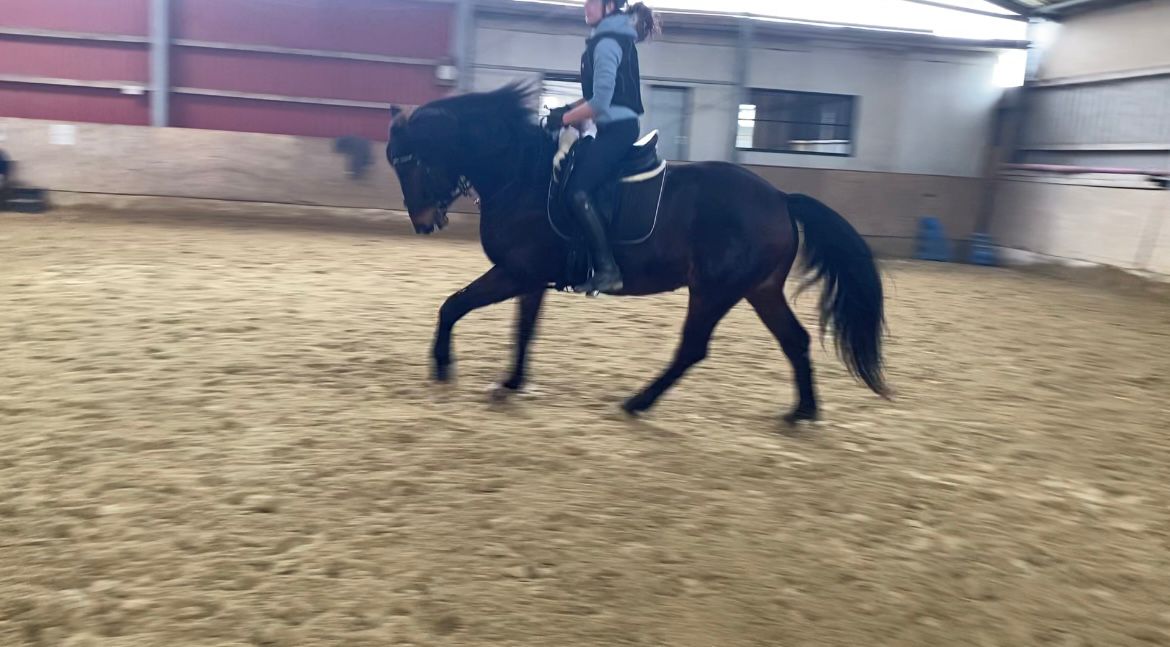

Supplement: Supplementary file 2 [file Data_Sheet_2.zip › Raw Data Main Text/Fig_9_1_no_case.jpg]

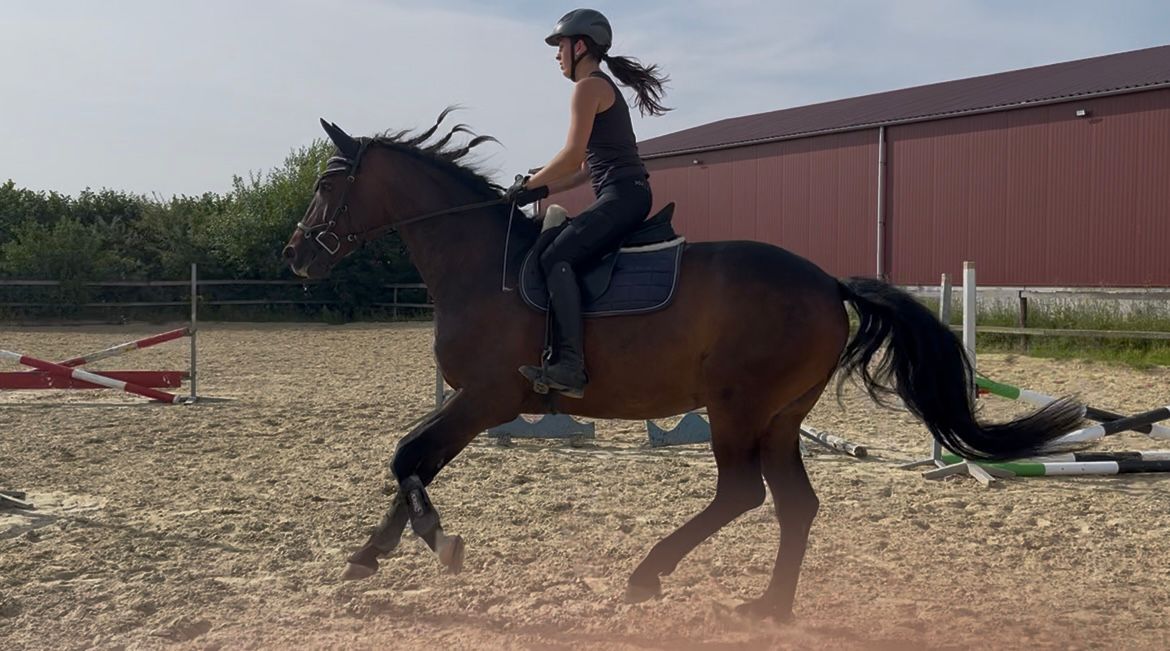

Supplement: Supplementary file 2 [file Data_Sheet_2.zip › Raw Data Main Text/Fig_9_2_no_case.jpg]
